# A Whole Leaf Comparative Study of Stomatal Conductance Models

**DOI:** 10.3389/fpls.2022.766975

**Published:** 2022-04-11

**Authors:** Gen Sakurai, Stanley J. Miklavcic

**Affiliations:** ^1^Institute for Agro-Environmental Sciences, National Agriculture and Food Research Organization, Tsukuba, Japan; ^2^Phenomics and Bioinformatics Research Centre, University of South Australia, Mawson Lakes, SA, Australia

**Keywords:** mathematical model, leaf model, photosynthesis rate, stomatal conductance, transpiration rate

## Abstract

We employed a detailed whole leaf hydraulic model to study the local operation of three stomatal conductance models distributed on the scale of a whole leaf. We quantified the behavior of these models by examining the leaf-area distributions of photosynthesis, transpiration, stomatal conductance, and guard cell turgor pressure. We gauged the models' local responses to changes in environmental conditions of carbon dioxide concentration, relative humidity, and light irradiance. We found that a stomatal conductance model that includes mechanical processes dependent on local variables predicts a spatial variation of physiological activity across the leaf: the leaf functions of photosynthesis and transpiration are not uniformly operative even when external conditions are uniform. The gradient pattern of hydraulic pressure which is needed to produce transpiration from the whole leaf is derived from the gradient patterns of turgor pressures of guard cells and epidermal cells and consequently leads to nonuniform spatial distribution patterns of transpiration and photosynthesis *via* the mechanical stomatal model. Our simulation experiments, comparing the predictions of two versions of a mechanical stomatal conductance model, suggest that leaves exhibit a more complex spatial distribution pattern of both photosynthesis and transpiration rate and more complex dependencies on environmental conditions when a non-linear relationship between the stomatal aperture and guard cell and epidermal cell turgor pressures is implemented. Our model studies offer a deeper understanding of the mechanism of stomatal conductance and point to possible future experimental measurements seeking to quantify the spatial distributions of several physiological activities taking place over a whole leaf.

## 1. Introduction

Over many years, several models of the interrelated concepts of stomatal conductance, leaf transpiration, and photosynthesis have been put forward. These have been discussed and analyzed at a localized level in many individual works. In a review of the very many different models available, Damour et al. (2010) categorized the models into distinct groups according to whether they were empirical or physical based, as well as according to their dependencies on environmental conditions. Although these models have been analyzed at a local level—i.e., limited to the immediate physical region associated with a single stoma—their respective impacts on long-ranged (non-local) phenomena, particularly the long-range responses to local changes in condition, has not previously been considered. It is by incorporating these models in an extended and interdependent setting, such as in a system of an interconnected network of nodes, that one can begin to evaluate the possible interaction of plant physiological responses taking place at distant points in a whole plant or a specific organ such as a leaf. Such a network should comprise a sufficient number of nodes and, thus, be of sufficient spatial extent, to ensure a significant physical (indeed hydraulic) separation between cause and effect.

Generally, the leaf hydraulic conductance is experimentally measured and discussed on a whole leaf scale (Prado and Maurel, 2013). However, even within a single leaf, the anatomical pathways from the petiole to local points in the leaf are different, as are the hydraulic conductances along those pathways. These differences create a gradation of hydraulic pressure in the leaf (Cochard et al., 2004), which affects the stomatal behavior at each local point in the leaf since the hydraulic conditions around guard cells affect the stomatal aperture different at those points (Buckley et al., 2003). This generates complex interactions between factors relating to the stomatal aperture. Therefore, to elicit quantitative information about any transport-related phenomena taking place at the cell, tissue, or indeed at the leaf level, it is arguably necessary to complement the whole leaf experimental studies with whole leaf theoretical modeling.

To derive information of a more specific and detailed nature *via* a comparison of theory with experiment, a dynamic (at least a steady state) model must be based on a reliable physiological understanding of leaf hydraulics. Indeed, as close a resemblance of a theoretical representation of all contributors to leaf conductance to the experimental definition as possible is critical. Many review articles (Sack and Holbrook, 2006; Damour et al., 2010; Prado and Maurel, 2013; Sack and Scoffoni, 2013; Stroock et al., 2014) identify and analyze the factors contributing to leaf conductance, starting with the hydraulic conductances of the xylem and of the phloem, and addressing the transport of water through the extravascular bundle tissue, the bundle sheath, and the mesophyll, then modeling the movement of liquid water into the epidermal layers, and then the movement of water vapor into the air spaces within the leaf (areoles). The essential next step is to construct a locally detailed but large scale integrated leaf model to study the effects of individual contributing factors and so bridge the divide between the whole leaf experimental studies and any local models.

In a recent article (Sakurai and Miklavcic, 2021), we introduced a hydraulic vasculature model of a dicot leaf which featured a 2×2D, coupled phloem-xylem networks of veins. The arrays span a hierarchical system of veins of different orders and, therefore, different hydraulic conductances through which water flowed. In the present study, we report on an advancement of this previous model to that of an extended 4×2D leaf hydraulic system (phloem-xylem-mesophyll-epidermis networks) which incorporates a stomatal conductance model. In this paper, we apply this model in a theoretical study of leaf hydrology, addressing the question of the influence of a local stomatal conductance model on overall leaf water transport and associated photosynthetic activity.

It is not expected to be useful to consider in an extended context, all local stomatal conductance models that have been proposed. Fortunately, one can take advantage of the categorization performed by Damour et al. (2010) and consider only representatives from each category. With this strategy, we consider here the implementation of three specific models of stomatal conductance. First, we consider the phenomenological (empirical) model of Leuning (1995), which incorporates, at some level, interaction with environmental humidity. However, because it is empirical, this model does not include the effects of turgor pressure of the cells around the stomatal aperture. Second, we implement the more detailed hydromechanical model of Buckley et al. (2003). This model is based around the intuitive assumption that the stomatal aperture, *a* (and hence stomatal conductance, *g*^*s*^), is linearly dependent on the difference in guard cell turgor pressure, *p*^*gu*^ and epidermal turgor pressure, *p*^*ep*^. Third, we apply a modified version of the Buckley et al. model in which the linear dependence is replaced by a non-linear relation between *a*, *p*^*gu*^, and *p*^*ep*^, established experimentally by Franks et al. (1998). In the present study, we refer to these last two models as the linear and non-linear Buckley et al. model, respectively. The characters of these three representative stomatal conductance models are analyzed within our whole leaf hydraulic model. We admit that the earlier model of Leuning (1995) has largely been superseded (a case in point is the refined hydromechanical model of Tuzet et al. (2003). Nevertheless, it is instructive to determine the extent to which, and under what conditions, the physically-based models predict different distribution patterns of the plant physiological responses to those predicted by an empirical model. We mention that this model is often applied to explain experimental results, though not necessarily in related contexts.

At this point it is important to mention the intricate model of stomatal regulation and control called OnGuard by its originators (Chen et al., 2012; Hills et al., 2012). The model, which featured in a recent review (Jezek and Blatt, 2017), is undoubtedly the most sophisticated model available describing stomatal function and its dependence on environmental conditions. This model was not available at the time of the Damour et al. (2010) review, but would nevertheless fall under the umbrella of a dynamical or mechanical model. For the purposes of the present study, we have opted to implement the Buckley et al. (2003) model (linear and nonlinear versions) for reasons of computational convenience. However, we anticipate that similar results would have been found with the OnGuard model of Hills et al. (2012) and Chen et al. (2012). Moreover, we envisage OnGuard being the more appropriate model to incorporate in a comprehensive, whole leaf transport model, when active transport mechanisms have been added.

The remainder of this article is dedicated to first describing our extended spatial steady-state leaf hydraulic model, and subsequently to describing the model's prediction of leaf transpiration and photosynthetic activity subject to various external conditions of humidity, CO_2_ concentration, leaf irradiance and temperature, and internal conditions of leaf shape and carboxylation rate. We compare the quantitative spatial distributions of transpiration and photosynthesis prescribed by the three models of stomatal conductance and correlate these with the spatial distributions of guard cell and epidermal cell turgor pressures. The article concludes with a few consequential comments and suggestions of possible future work.

## 2. Method

### 2.1. General Description of the Physical Model

**Figure 1:**
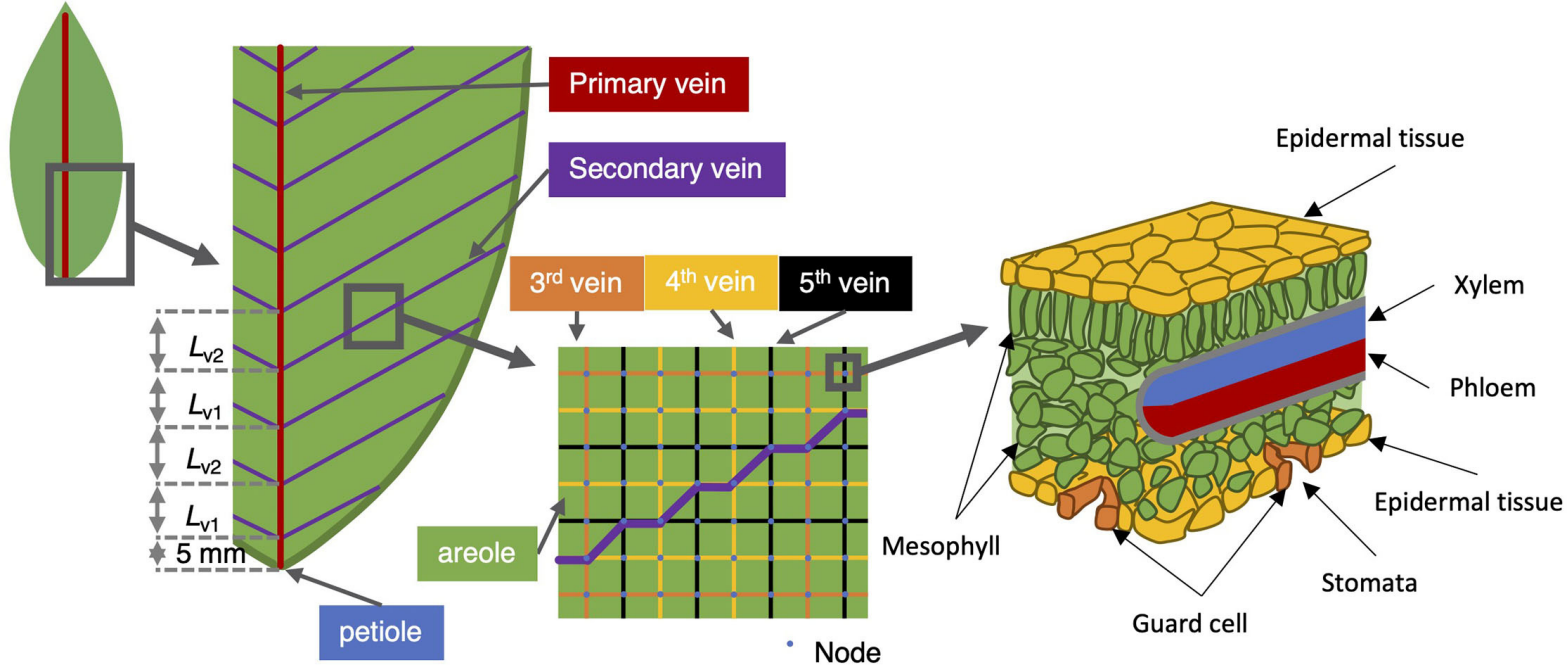
Vein architecture of the model leaf. The angle of the second-order vein to the main vein is kept fixed at 45°, the higher order veins are laid out as a rectangular grid (middle sub-panel). The right-most sub-panel shows a schematic of the tissue structure presumed at each node.

Overall, we have based our model on the physical principles of diffusion and convection, in direct correspondence with the model published in Sakurai and Miklavcic (2021). This is analogous to the passive model one of us developed to describe water and solute transport in plant roots (Foster and Miklavcic, 2013, 2014, 2016). Fluid flow is driven by pressure differences whether hydraulic (water potential) or osmotic (turgor). The physical model assumes water transport through the xylem is ultimately driven by hydraulic pressure differences between the petiole (the source) and transpiration points (sinks). Our revised model includes a more detailed representation of the pathways that water may travel outside the vascular bundle. We maintain the direct link between xylem and phloem that was featured in Sakurai and Miklavcic (2021), but at every node of the 2D xylem network in this extended model, the pathway of fluid flow through the leaf includes movement through other tissue regions in addition to the phloem: specifically the mesophyll and the epidermis (Figure 1). At termini of pathways through the tissue regions, which is assumed the case at all nodes, we impose a model of stomatal conductance and its respective dependence on external conditions. We set the latter boundary (i.e., external) conditions as fixed relative humidity, fixed CO_2_ concentration, constant leaf irradiance, and constant leaf temperature; the standard condition is 50%, 400 ppm, 500 μmol m^−2^ s^−1^, and 25°C (298.15 K), respectively. It is important to note that these conditions are prescribed uniformly over the entire leaf. That is, there is no variation of these conditions over the leaf, which would, thus, be consistent with an ideal physical setting (although we acknowledge that variation across a leaf, especially of illumination, is possible in a non-laboratory situation).

At the petiole, we assume a fixed hydraulic pressure in the xylem. As for the phloem, with the model it is possible to consider conditions of (a) a severed leaf in which case we assume the phloem at the petiole becomes blocked through the coagulation of macromolecules resulting in the condition of zero sucrose and sap flux through the phloem, or (b) an intact leaf in which case there is assumed a continuity of passage of both sucrose and sap through the phloem at the petiole from the leaf to its connective plant stem—the appropriate conditions are then those of constant solute and sap fluxes.

**Figure 2:**
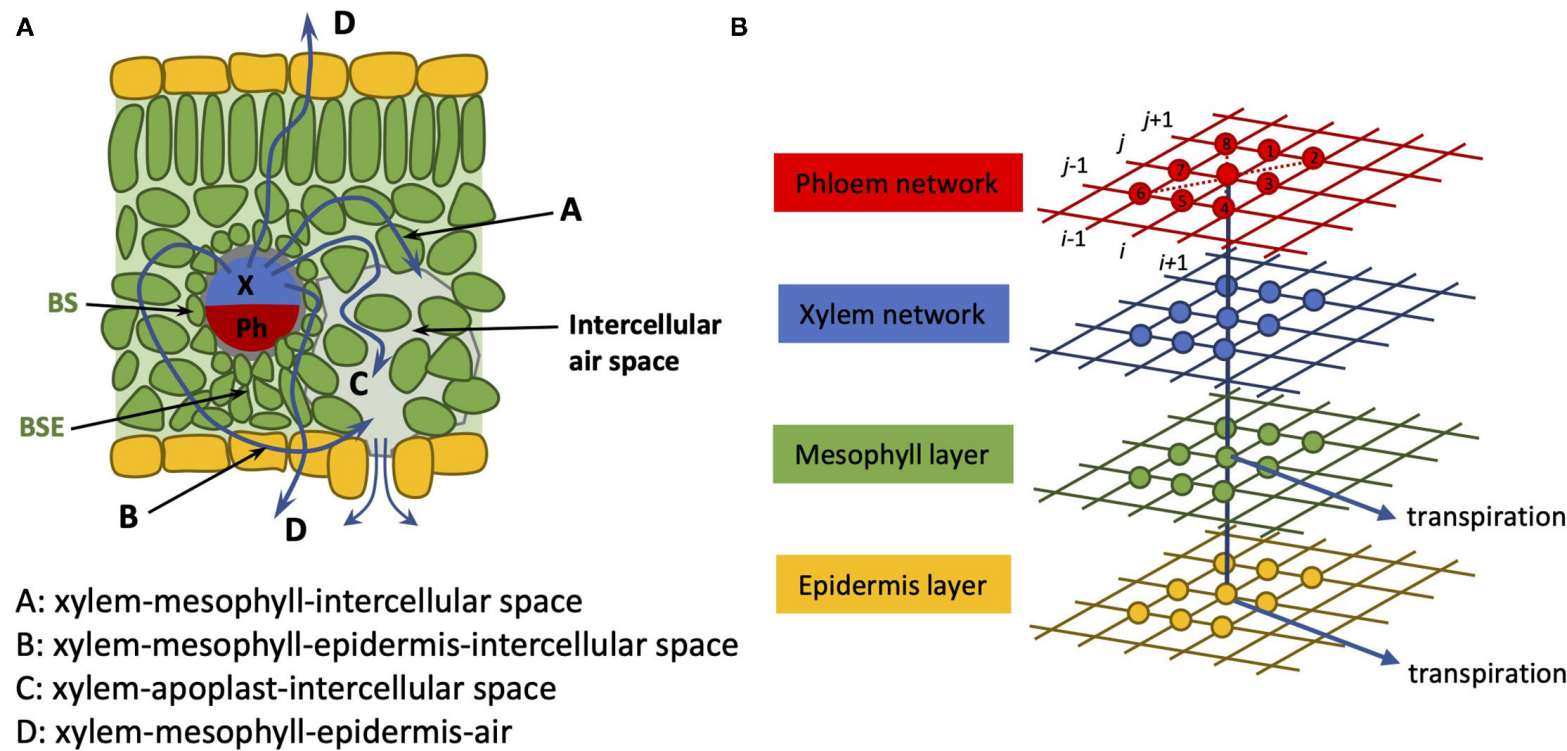
**(A)** Cartoon of leaf anatomy in cross-section; **(B)** Coupled 2D networks of tissue pathways. The xylem network (blue grid) is connected on the one hand to a phloem network (red grid) and on the other hand to an epidermis network (yellow grid) through a (mesophyll network) (green grid). Transpiration (blue arrows) is assumed to occur through both the epidermis network and through the mesophyll network. As in our earlier article, the nodes are numbered in a grid-like fashion: (*i, j*) = (1, 1)…(*N, M*), as indicated by the black numbering. The identities of nearest and next-nearest neighbor nodes to node (*i, j*) are identified at the leaf creation stage and recorded for later recall. The flow occurs between nearest and next-nearest neighbor nodes.

In incorporating the mesophyll in this extended model, we collect the mesophyll cells corresponding to a given node into one entity and treat that part of the pathway as an amalgamation of both apoplastic and symplastic pathways. This is not unlike the treatment followed in Foster and Miklavcic (2013, 2014) for transport in a plant root. The physical pathway between a xylem node and an epidermis node is, thus, here assumed to be *via* a single mesophyll node. As we treat the connection with the mesophyll as a concatenation of apoplastic and symplastic flows, it is appropriate to consider this xylem-to-epidermis pathway as being in series with the mesophyll node (refer to Figure 2). This amalgamation should include not only the water phase pathway but also the contribution of vapor phase transport to leaf hydraulic conductance (Buckley, 2015, 2017; Buckley et al., 2015). We note, however, that a time-dependent analysis would require the separation of these pathways to properly consider the capacitance effect of the mesophyll.

According to Esau (1953), heterobaric leaves possess significant vascular bundle sheath extensions (BSEs) interposed between the xylem-phloem vascular bundles and the epidermis. These may be large for the low order veins and may be nonexistent in small vascular bundles. In all cases, we have taken the step to simply incorporate these into the definition of the mesophyll, although for future reference, these may be discriminated against as they feature properties other than simply lengthening the pathway between the xylem and the epidermis. For example, through the lignification of the surface exposed to internal air spaces in the spongy mesophyll (areoles), they may modify the ability of water to evaporate inside the leaf or otherwise limit the transverse movement of water vapor.

In Sakurai and Miklavcic (2021), the reticulate vein system lacked the realism of tapered veins. However, in a true leaf of (at least) some angiosperms, the vascular bundle entering the leaf through the petiole comprises a bundle of veinlets whose number progressively diminishes as the groups diverge at regular intervals to become the next order vein. A vein under consideration, thus, diminishes in transverse diameter in discrete steps until the vein terminates or conjoins with higher order veins terminating at stomata or the leaf blade edge. In the present revised model, the first and second order veins are here subjected to tapering. We imagine that higher order veins exceed the limit of veinlet divergence and so possess uniform (low) conductivity until their termini at which point their hydraulic resistance becomes infinite. A summary of results and a few brief comments on the comparison between tapered and non-tapered leaves are provided in Supplementary Figures SI.1–SI.3.

### 2.2. Details of the Mathematical Model

#### 2.2.1. Tissue Structure

In our previous model (Sakurai and Miklavcic, 2021), the complex leaf vein architecture of a leaf is presented as two layers of two-dimensional networks of xylem and phloem, in which each layer is structured with multiple nodes. The phloem network connects to the two-dimensional xylem network at corresponding nodes. In the present model, two additional layers are added: the mesophyll layer and the epidermis layer. The mesophyll layer connects to the xylem network, and the epidermis layer connects to the mesophyll layer at corresponding nodes. In this model, these four layers represent the structure of a whole leaf (Figure 2). We represent the whole leaf shape within a rectangular area defined by *N* × *M* = 65 × 130 = 8, 450 nodes. The number of nodes where the leaf exists in this 2D area is 3,138. Therefore, the total number of leaf nodes in all four layers is 3, 138 × 4 = 12, 552.

#### 2.2.2. Governing Equations—Hydraulic Transport

Here, we describe the essential equations of the present model. A more detailed explanation of the model is described in the file Supplementary Information 1. Note that, in the following equations, the superscripts with roman symbols do not indicate exponentiation, instead they are employed to represent the position in the leaf (e.g., mesophyll layer or guard cells). At the (*i, j*)th node of the xylem, phloem, mesophyll, or epidermis (hereafter abbreviated to *ij*, for *i* = 1, …, *N, j* = 1, …, *M*), we apply the conservation constraint of zero net water flux out of the node. The conservation constraint asserts that the sum of the fluxes in the eight horizontal directions ($\overset{8}{\underset{d=1}{\sum }}F_{ij-d}$) (refer to Table 1), plus the sum of the flux(es) to the node(s) of other layer(s) ($\overset{2}{\underset{v=1}{\sum }}F_{ij-v}^{\text{layer}:k}$) and transpiration ($F_{ij-\text{T}}^{\text{layer}:k}$), is equal to zero. Therefore, we can describe it as follows.


(1) $$\begin{array}{l} \overset{8}{\underset{d=1}{\sum }}F_{ij-d}^{\text{layer}:k}+\overset{2}{\underset{v=1}{\sum }}F_{ij-v}^{\text{layer}:k}+F_{ij-\text{T}}^{\text{layer}:k}=0,\\ \;\left(i,j,k\right)=\left(1,1,1\right),\ldots ,\left(N,M,4\right). \end{array}$$

A list of dependent variables critical to the numerical model is given in Table 2. In the above equation, the superscript *k* (*k* = 1, …, 4) indicates the position of the layer (phloem: 1, xylem: 2, mesophyll: 3, and epidermis: 4). The subscript *v* represents the direction of the flow between the layers (*v* = 1 represents the flow from *k* to *k* − 1 and *v* = 2 represents the flow from *k* to *k* + 1). For example, $\overset{2}{\underset{v=1}{\sum }}F_{ij-v}^{\text{layer}:2}$ represents the sum of the fluxes from the xylem to the phloem and from the xylem to the mesophyll. From the structural definition of the model, $F_{ij-1}^{\text{layer}:1}$ and $F_{ij-2}^{\text{layer}:4}$ equal to zero (Figure 2). Moreover, we assumed $F_{ij-\text{T}}^{\text{layer}:1}=0$ and $F_{ij-\text{T}}^{\text{layer}:2}=0$ for simplicity. Note that, we define water flow out of a node as negative.

**Table 1:** Xylem flux directions (d).

**Number (d) **	**Direction **	**Variable name**
(1)	North	$F_{ij-1}^{xyl}$
(2)	North-east	$F_{ij-2}^{xyl}$
(3)	East	$F_{ij-3}^{xyl}$
(4)	South-east	$F_{ij-4}^{xyl}$
(5)	South	$F_{ij-5}^{xyl}$
(6)	South-west	$F_{ij-6}^{xyl}$
(7)	West	$F_{ij-7}^{xyl}$
(8)	North-west	$F_{ij-8}^{xyl}$

**Table 2:** Summary of function variables, at node *ij* (subscripts are not shown).

**Symbol**	**Description**	**Units**
*F* ^layer:*k*^	Xylem water flux at layer *k*	mmol s^−1^
*K* ^layer:*k*^	Conductance at layer *k*	mmol s^−1^ MPa^−1^
*p* ^layer:*k*^	Hydraulic or turgor pressure at layer *k*	MPa
*T*	Temperature of the leaf	K
*C* ^layer:*k*^	Sucrose concentration at layer *k*	mol m^−3^
*g*_W_	Stomatal conductance (water vapor)	mol s^−1^ m^−2^
*D* ^s^	Difference in water vapor mole fraction between the intercellular spaces and the air	mmolmol^−1^
*S*	Sucrose flux	mol *s*^−1^
*P*	Photosynthesis rate	μmol s^−1^ m^−2^
Γ	Photorespiratory compensation point (Pa)	Pa
$\theta_{c}^{\text{i}}$	Intercellular CO_2_ partial pressure	Pa
*W*_c_	Carboxylation rate limited by CO_2_ and Rubisco, but not by RuBP	μmol s^−1^ m^−2^
*W*_*j*_	Carboxylation rate limited by RuBP and CO_2_, but not by Rubisco	μmol s^−1^ m^−2^
*g*_c_	Stomatal conductance (CO_2_)	mol s^−1^ m^−2^
γ^s^	CO_2_ concentration at the leaf surface	ppm
Γ′	Photorespiratory compensation point (ppm)	ppm
*D* ^Pa^	Vapor pressure difference (Pa)	Pa
*p* ^gu^	Turgor pressure at guard cells	MPa

*The subscripts d (horizontal direction), v (between layers), and T (transpiration) are also not shown*.

The fluid motion in each layer is driven by hydraulic pressure, founded on Darcy's law of plug flow (Batchelor, 1967): μ*u* = −ι∇*p*, expressing the fact that in the conduit between two consecutive nodes the fluid velocity, *u*, is proportional to the pressure gradient across the conduit joining those nodes (∇*p*), with the vein conductance (ι/μ) being the coefficient of proportionality; here ι is a fluid permeability (m^2^) and μ is the fluid viscosity (Pa.s). In the phloem, mesophyll, and epidermis, fluid motion is driven by not only hydraulic but also by osmotic pressure. Darcy's law must then be modified to include an osmotic pressure contribution resulting from a concentration difference: Π = −α*RTΔC*, with *R* as the universal gas constant, *T* as temperature, and *C* as the local concentration of solute at the node. The parameter α is a proportionality constant. In discrete form,


(2) $$\begin{array}{l} F_{ij-d}^{\text{layer}:k}=\\ K_{ij-d}^{\text{layer}:k}\left(\left(p_{ij}^{\text{layer}:k}-p_{d}^{\text{layer}:k}\right)-\sigma_{ij-d}^{\text{layer}:k}RT\left(C_{ij}^{\text{layer}:k}-C_{d}^{\text{layer}:k}\right)\right),\\ \;\left(i,j,k\right)=\left(1,1,1\right),\ldots ,\left(N,M,4\right), \end{array}$$

where $F_{ij-d}^{\text{layer}:k}$ is the (signed) fluid volume flux *from* a node *ij* *to* the node connected to it in the direction *d* (one of eight neighbors) at layer *k*. The position dependent parameter $\sigma_{ij-d}^{\text{layer}:k}$ is called the reflection coefficient (Katchalsky and Curran, 1965; Foster and Miklavcic, 2014, 2016; Sakurai and Miklavcic, 2021). In this equation, $\sigma_{ij-d}^{\text{layer}:1}$ (phloem) and $\sigma_{ij-d}^{\text{layer}:2}$ (xylem) are equal to zero since the solute movement is assumed not to be impeded (Kramer and Boyer, 1995). $K_{ij-d}^{\text{layer}:k}$ is the fluid conductance between the *ij*th node and its neighbor in the direction *d* (refer to Table 3 for the xylem and the phloem).

**Table 3:** Xylem vein conductivities; phloem conductivities are a factor of 3/100 lower.

**Vein order **	**Conductance **	**Vein diameter**
** **	**(m mmol s^**−1**^ MPa^**−1**^) **	**(μm)**
1	1.00×10^−2^	15.50
2	5.00×10^−4^	11.30
3	6.00×10^−5^	7.50
4	4.00×10^−5^	7.20
5	4.00×10^−6^	4.34

The (signed) volume flux of water from a node of one layer to the corresponding node of another layer is expressed by the relation:


(3) $$\begin{array}{l} F_{ij-1}^{\text{layer}:k}=-F_{ij-2}^{\text{layer}:k-1}\\ \text{ =}K_{ij-\text{c}}^{k:k-1}(\left(p_{ij}^{\text{layer}:k}-p_{ij}^{\text{layer}:k-1}\right)-\sigma_{ij-\text{c}}^{k:k-1}RT(C_{ij}^{\text{layer}:k}\\ \;-C_{ij}^{\text{layer}:k-1})),\\ \;(i,j,k)=(1,1,1),\ldots ,(N,M,4). \end{array}$$

where $K_{ij-\text{c}}^{k:k-1}$ is the conductance of the route between the two nodes, and $\sigma_{ij-\text{c}}^{k:k-1}$ is a reflection coefficient for this pathway, which is here set to unity (i.e., $\sigma_{ij-\text{c}}^{k:k-1}=1$).

For simplicity, the fluxes of water as the transpiration process from the mesophyll (*k* = 3) and the epidermis (*k* = 4), $F_{ij-\text{T}}^{\text{layer}:3}$ and $F_{ij-\text{T}}^{\text{layer}:4}$, are determined as being proportional to the total transpiration rate from the stomata in the node *ij* ($F_{ij-\text{T}}^{\text{s}}$). For each node, the total transpiration rate is represented as the product of conductance ($g_{ij-\text{w}}^{\text{all}}$) and leaf-to-boundary layer H_2_O mole fraction gradient ($D_{ij}^{\text{s}}$). Therefore, $F_{ij-\text{T}}^{\text{layer}:3}$ and $F_{ij-\text{T}}^{\text{layer}:4}$ are:


(4) $$\begin{array}{l} F_{ij-\text{T}}^{\text{layer}:k}=e^{\text{layer}:k}F_{ij-\text{T}}^{\text{s}}\\ =-e^{\text{layer}:k}\left(a_{ij}g_{ij-\text{w}}^{\text{all}}D_{ij}^{\text{s}}\right)\\ \;\left(i,j,k\right)=\left(1,1,3\right),\ldots ,\left(N,M,4\right), \end{array}$$

where *a*_*ij*_ is the 2D grid area assigned to that node *ij* and *e*^layer:*k*^ is proportional constants (*e*^layer:3^+*e*^layer:4^ = 1). Note again that the transpiration rate is defined here to be negative since we define water flow out of a node as negative.

#### 2.2.3. Governing Equations—Solute Transport

In direct analogy with the water fluxes, we assume conservation of sucrose fluxes. Namely, we specify that the sum of all sucrose fluxes into and out from a given node in the eight lateral directions (*S*_*ij*−1_, …, *S*_*ij*−8_), plus a contribution from sucrose loading into the sieve tube (*S*_*ij*−L_) should be equal to zero:


(5) $$\begin{array}{l} \overset{8}{\underset{d=1}{\sum }}S_{ij-d}+S_{ij-\text{L}}=0. \end{array}$$

In the above equation, the sucrose loading into the sieve tube (*S*_*ij*−L_) is calculated as *S*_*ij*−L_ = Λ_*ij*_*a*_*ij*_, where Λ_*ij*_ is the local sucrose loading rate per unit area, and we assume that this rate is related to the photosynthesis rate *P*_*ij*_.

#### 2.2.4. Governing Equations—Stomatal Conductance

As the mathematical model of photosynthesis, the model by Farquhar et al. (1980) is applied. In the model, the net photosynthesis rate *P*_*ij*_ is determined by the Rubisco-limited carboxylation rate, *W*_c_, and the RuBP-limited (Ribulose-1,5-bisphosphate-limited) carboxylation rate, *W*_j_:


(6) $$\begin{array}{l} P_{ij}=\left(1-\frac{\Gamma_{ij}}{\theta_{ij-\text{c}}^{\text{i}}}\right)\cdot \min\left\{W_{ij-\text{c}},W_{ij-\text{j}}\right\}-H_{ij},\\ \;\left(i,j\right)=\left(1,1\right),\ldots ,\left(N,M\right). \end{array}$$

where Γ_*ij*_ is the photorespiratory compensation point, $\theta_{ij-\text{c}}^{\text{i}}$ is the intercellular CO_2_ partial pressure, and *H*_*ij*_ is the respiration rate (Farquhar et al., 1980; Buckley et al., 2003).

For the description of stomatal conductance, one of three models is used: the stomatal conductance model proposed by Leuning (1995), or one of two versions of the stomatal conductance model proposed by Buckley et al. (2003).

In the model proposed by Leuning (1995), the relationship between stomatal conductance, photosynthesis rate, relative humidity, and CO_2_ concentration is described by:


(7) $$\begin{array}{l} g_{ij-\text{c}}^{\text{s}}g_{0}^{\text{s}}+b_{\text{l}}\frac{P_{ij}}{\left(\gamma^{\text{s}}-\Gamma_{ij}^{\prime }\right)\left(1+D_{ij}^{\text{Pa}}/D_{0}\right)},\\ \;\left(i,j\right)=\left(1,1\right),\ldots ,\left(N,M\right), \end{array}$$

where *D*_0_ and *b*_l_ are empirical coefficients, γ^s^ is the CO_2_ concentration at the leaf surface, $\Gamma_{ij}^{\prime }$ is the photorespiratory compensation point, $D_{ij}^{\text{Pa}}$ is the humidity deficit at the leaf surface, *P*_*ij*_ is the photosynthesis rate, and $g_{0}^{\text{s}}$ is the stomatal conductance when *P*_*ij*_ is zero. In this model, the variables also interact with each other: the stomatal conductance is affected by the photosynthesis rate, and photosynthesis itself is dependent on stomatal conductance.

The model of Buckley et al. (2003) is one of the more complicated models devised, in which mechanical processes including the stomatal opening *via* the increase of the turgor pressure of the guard cells is considered. In their model, they assumed that stomatal conductance (*g*^s^) is proportional to the stomatal aperture which, in turn, is determined by the turgor pressures of the guard cells (*p*^gu^) and epidermal cells (*p*^layer:4^):


(8) $$\begin{array}{l} g_{ij}^{\text{s}}=\chi \left(p_{ij}^{\text{gu}}-\hat{m}p_{ij}^{\text{layer}:4}\right), \end{array}$$

where χ is a proportionality constant, and $\hat{m}$ is the mechanical advantage of the epidermis (Buckley et al., 2003), whose value is usually greater than 1, expressing the effect of a difference between the turgor pressure of epidermal cells and those of the guard cells (Franks et al., 1998). In their model, the authors assume that the turgor pressure of a guard cell $p_{ij}^{\text{gu}}$, *via* the guard cell osmotic pressure, is controlled by the ATP supply rate, as the driving force of proton pumping of potassium. Moreover, they assume that $p_{ij}^{\text{gu}}$ is controlled by the turgor pressure of the epidermal cells around the guard cell. Therefore, in this model, the stomatal conductance, the photosynthesis rate, and the turgor pressure interact in a complex way. The supply rate of ATP is related to the rate of photosynthesis production, and (Buckley et al., 2003) associate the ATP concentration in the guard cells with the rate of RuBP carboxylation. In turn, the rate of carboxylation that can be sustained is determined by the current rate of electron transport, as based on the model by Farquhar and Wong (1984) (refer to Supplementary Information I for details).

In the nonlinear version of Buckley et al.'s stomatal conductance model, Equation (8) is replaced by


(9) $$\begin{array}{l} g_{ij}^{\text{s}}=\frac{\chi }{c_{\text{F}}}a=\chi \left[f_{1}\left(p_{ij}^{\text{gu}}\right)-\frac{p_{ij}^{\text{layer}:4}}{p_{\text{F},\text{max}}^{\text{ep}}}\left(f_{1}\left(p_{ij}^{\text{gu}}\right)-f_{2}\left(p_{ij}^{\text{gu}}\right)\right)\right], \end{array}$$

where *f*_1_ and *f*_2_ are two nonlinear functions of guard cell turgor pressure. *c*_F_ and $p_{\text{F},\text{max}}^{\text{ep}}$ are parameters. These have been established, experimentally, by Franks et al. (1998) to have forms resembling the following functions:


(10) $$\begin{array}{l} f_{1}\left(p^{\text{gu}}\right)f_{1}^{\infty }\left(1-e^{-p^{\text{gu}}/p_{1,0}^{\text{gu}}}\right);\\ f_{2}\left(p^{\text{gu}}\right)\frac{f_{2}^{\infty }}{2}\left(\tanh\left(p^{\text{gu}}-p_{2,0}^{\text{gu}}\right)+1\right), \end{array}$$

where $f_{1}^{\infty }$ and $f_{2}^{\infty }$ are some constants representing limiting values of *f*_1_ and *f*_2_ at high turgor pressures, and the two $p_{1,0}^{\text{gu}}$ and $p_{2,0}^{\text{gu}}$, parameters are constant turgor pressure scalings. In the present study, however, we have chosen to follow the lead of Franks et al. (1998) and use the so-called sigmoidal functional form.


(11) $$\begin{array}{l} f_{1\left(2\right)}\left(p^{\text{gu}}\right)=\frac{f_{1\left(2\right)}^{\infty }}{1+e^{-\phi_{1\left(2\right),1}^{\text{gu}}\cdot p^{\text{gu}}+\phi_{1\left(2\right),2}^{\text{gu}}}}, \end{array}$$

for both *f*_1_ and *f*_2_, but with different fitting parameters $f_{1}^{\infty }$, $f_{2}^{\infty }$, $\phi_{1,1}^{\text{gu}}$, $\phi_{2,1}^{\text{gu}}$, $\phi_{1,2}^{\text{gu}}$, and $\phi_{2,2}^{\text{gu}}$. These constants are determined by fitting such mathematical functions to measured data sets (refer to **Figure 4** in Franks et al. 1998).

The subsequent details of the Buckley et al. (2003) model remain the same in this nonlinear version as in the original version. Note that, also in the Leuning model, we calculated guard cell turgor pressure following the Buckley et al. model even though solute concentration in the guard cells is not defined in the Leuning model. To be precise, in our whole leaf Leuning model, only the equation for calculating stomatal conductance derives from the Leuning stomatal conductance model. Therefore, the results of the guard cell turgor pressure in our Leuning implementation are actually a mixture of Leuning and Buckley et al. models, referred to simply as Leuning model results.

#### 2.2.5. Parameter Settings

It bears noting that since the stomatal conductance models being examined here are based on somewhat different premises, the parameters on which they rely are correspondingly different. In order to pursue a meaningful comparison, we have taken the following steps: we have retained many of the original parameter values as presented in the original studies, with the exception of $g_{0}^{\text{s}}$ and *D*_0_ appearing in the model by Leuning (1995) and *c*_F_ appearing in the model by Buckley et al. (2003). These values were modified in order to produce leaf-average transpiration rates that agreed with all three models, under the specific conditions of medium levels of CO_2_, light intensity, and relative humidity.

It may be argued that (in lieu of any nonlinear feedback mechanisms affecting these parameters) the resulting, and respective, parameter sets are not expected to change in response to a change in environmental conditions. Hence, it may be said that the choices of the values of these parameter sets correspond to the same plant species. This assumption underpins the model comparison that we make under a range of external conditions.

The complete set of model parameters called upon in our calculations is listed in Supplementary Tables 1–4. The chosen parameter values are collected and presented in Supplementary Tables 5–8.

#### 2.2.6. Environmental Settings

As a reasonable baseline scenario, we set CO_2_ concentration as 400 ppm, relative humidity as 50%, and irradiance as 500 μmol m^−2^ s^−1^. We set this as the medium environmental setting. The temperature was set as 25°C (298.15 K) everywhere (at the leaf surface and inside the leaf) for simplicity. The other scenarios considered in our simulations are summarized in Supplementary Information I. In our simulation study of humidity, we considered three cases of relative humidity: 10, 50, and 90%. In the simulation study of CO_2_ concentration, we considered the concentrations: 100, 400, and 800 ppm. Next, we have considered three values of light intensity: 200 μmol m^−2^ s^−1^, 500 μmol m^−2^ s^−1^, and 1,000 μmol m^−2^ s^−1^. Finally, some of our simulations, varying the above three external conditions, were repeated at the lower temperature of 10°C (283.15 K).

In this study, the equations were implemented using MATLAB 2021a. The approximate solutions of the simultaneous equations were calculated using the fsolve optimization function of the Optimization Toolbox of MATLAB. The target variables solved for using the optimization process were xylem hydraulic pressure (*p*^xyl^), phloem turgor pressure (*p*^ph^), mesophyll turgor pressure (*p*^mes^), epidermal turgor pressure (*p*^epi^), phloem sucrose concentration (*C*^ph^), and intracellular CO_2_ concentration (γ^i^). We numerically optimized the values by assuming concentration conditions where the sum of the fluxes of water, sucrose, or CO_2_ at each node of xylem, phloem, mesophyll, or epidermis equals zero. The figures were generated using R.

## 3. Results

### 3.1. General Overview of Model Results

**Figure 3:**
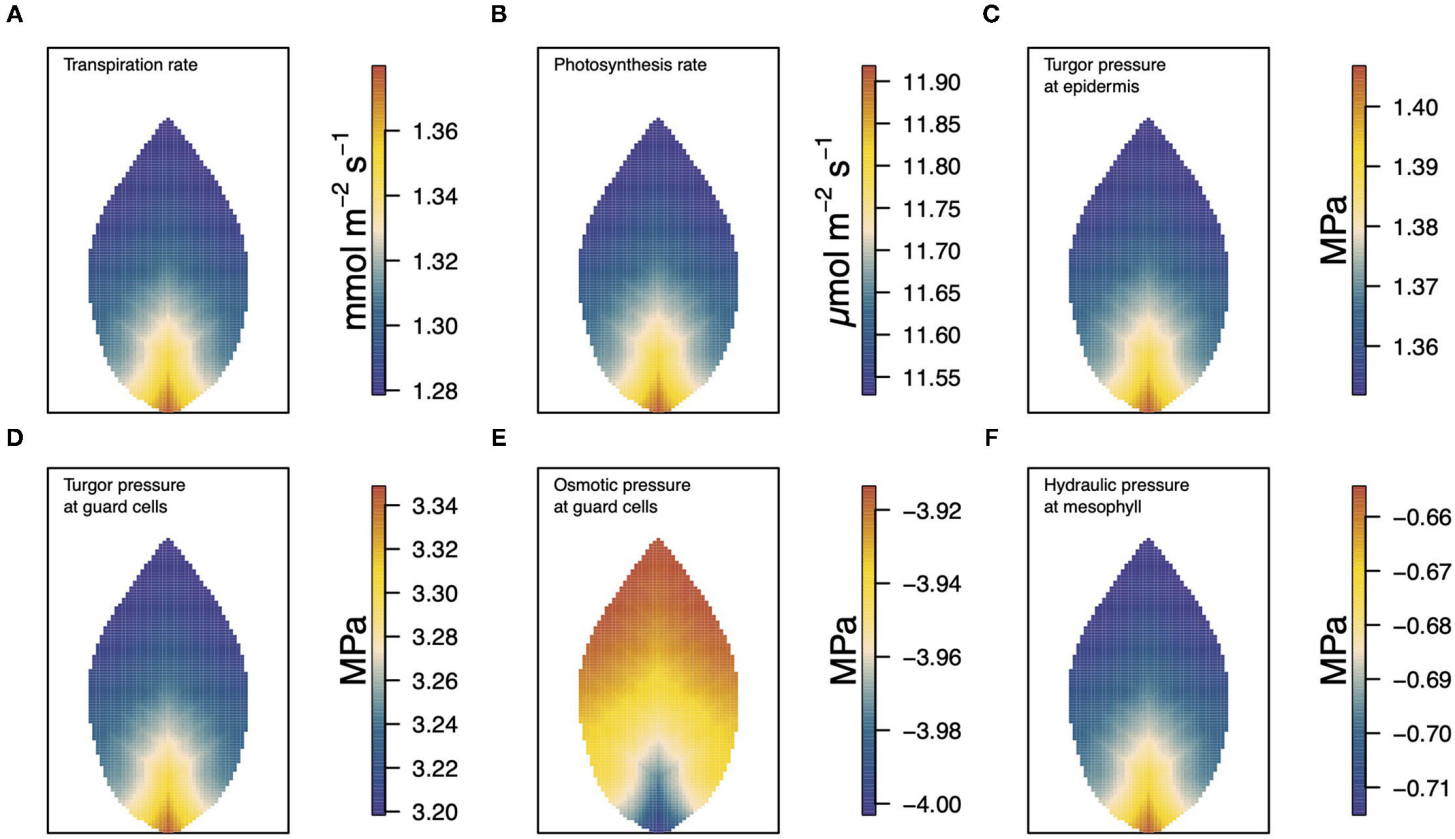
A summary figure showing the relationships between **(A)** transpiration, **(B)** photosynthesis, **(C)** epidermal turgor pressure, **(D)** guard cell turgor pressure, **(E)** guard cell osmotic pressure, and **(F)** water potential in the mesophyll. The results, based on the linear (Buckley et al., 2003) model of stomatal conductance were generated under medium level conditions of 50% humidity, 500 μmol m^−2^ s^−1^ irradiance, and 400 ppm CO_2_ concentration.

In Figure 3, we show a collective summary of the results of the model for a single scenario calculation. Results of other cases and scenarios, and under other conditions, can be found in subsequent figures. The six panels show, from (a)−(f), the distributions of transpiration, photosynthesis, epidermal turgor pressure, guard cell turgor pressure, guard cell osmotic pressure, and mesophyll water potential. These steady-state distributions were determined using the linear Buckley et al. model of stomatal conductance, assuming vein tapering, and under the medium-level, environmental conditions of 50% humidity, 500 μmol m^−2^ s^−1^ irradiance, and 400 ppm CO_2_ concentration. Through these figures, we see a strong positive correlation between many of the quantities shown but also a direct negative correlation between others.

The turgor pressure of the guard cell, *p*^gu^ in panel (d), can be partitioned into its contributing parts, the osmotic pressure (e) and the hydraulic pressure. Here, the sum of these is dominated by the osmotic component which, under these favorable conditions, is due to a high sucrose concentration (the guard cell hydraulic pressure values are similar to those of the mesophyll (panel (f)) and, hence, are not shown here). According to the linear (Buckley et al., 2003) model, the stomatal conductivity is dependent on the difference between the turgor pressures of the guard cell and surrounding epidermal cells. As such it is essentially the difference between panels (c) and (d) that is the basis for the level of transpiration shown in panel (a). Panel (b) shows the distributed production of photosynthetic products over the leaf, which is governed in turn by the local intracellular concentration of CO_2_ (in our model, this is confined to the general mesophyll-epidermis region). As with other variables, these distributions are nonuniform despite uniform external conditions of temperature, humidity, CO_2_ concentration, and light intensity.

### 3.2. Comparison of Stomatal Models

#### 3.2.1. Dependence on CO_2_ Concentration

**Figure 4:**
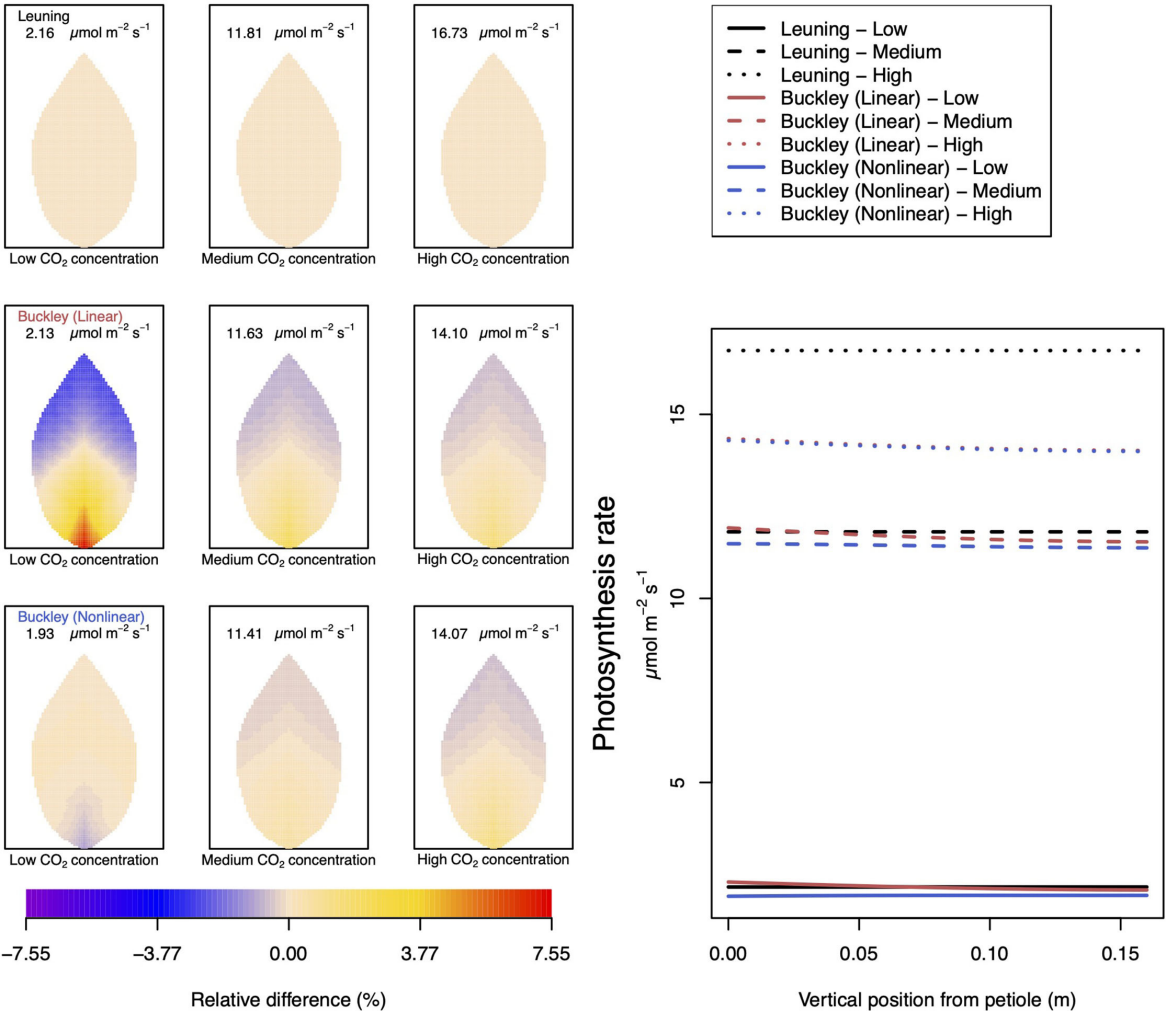
A comparison of predictions of photosynthetic activity distributed over the leaf according to the three stomatal conductance models of Leuning (1995) (top row of panels and black lines in right hand figure), linear (Buckley et al., 2003) (middle row of panels and red lines in the right hand figure), and nonlinear (Buckley et al., 2003) (bottom row of panels and blue lines in right hand figure). The 2D maps shown are deviations from leaf-area averages. The leaf-area average values of photosynthesis rates are given at the top of each panel. The three columns (left-to-right) and line styles (solid, dashed, dotted) show the respective dependencies on the **external gas concentration of CO**_**2**_: low (100 ppm), medium (400 ppm), and high concentration (800 ppm). Other model parameter values can be found in Supplementary Information I.

**Figure 5:**
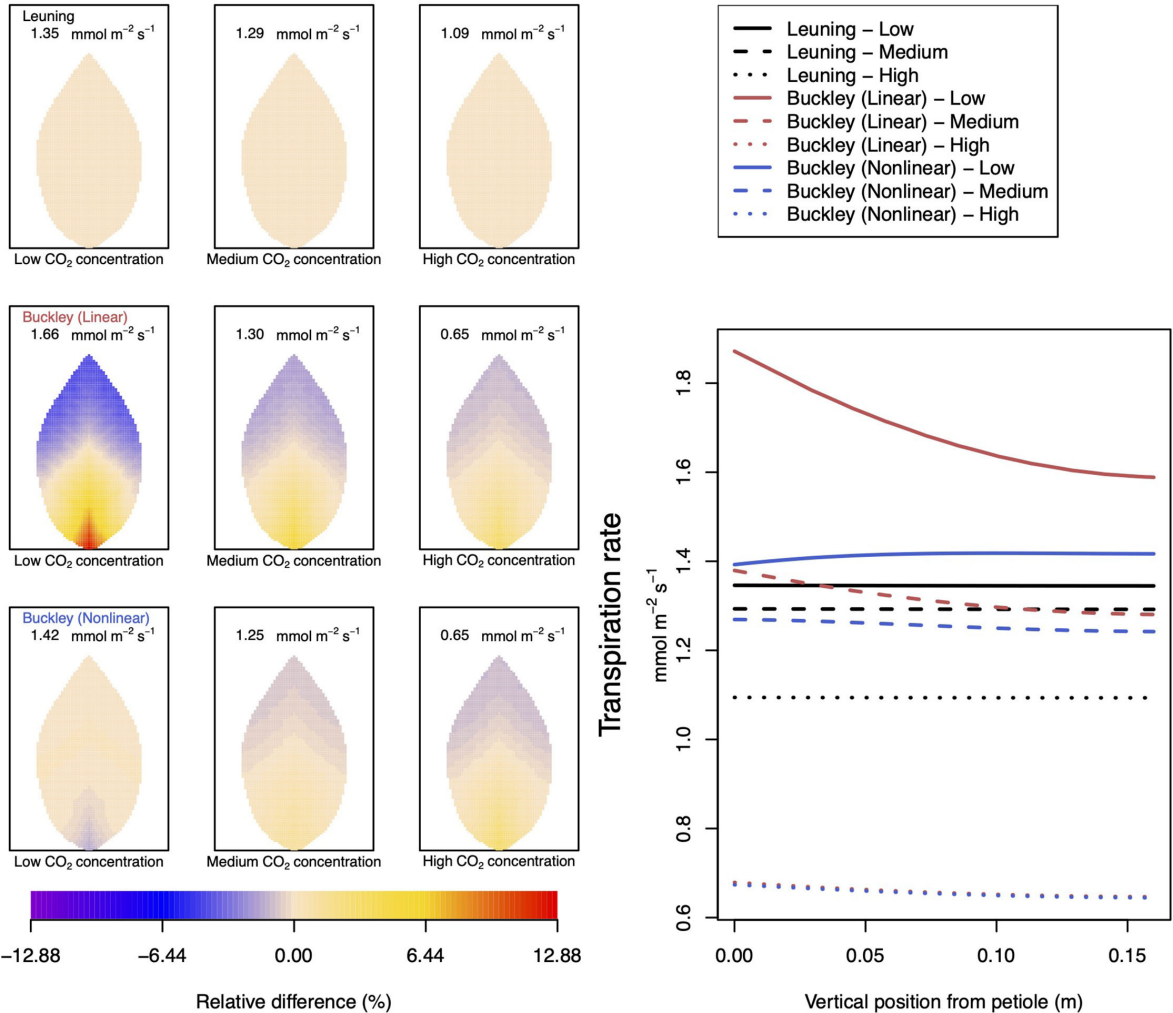
A comparison of predictions of transpiration (−*F*_*T*_) distributed over the leaf according to the three stomatal conductance models of Leuning (1995) (top row of panels and black lines in right hand figure), linear (Buckley et al., 2003) (middle row of panels and red lines in the right hand figure), and nonlinear (Buckley et al., 2003) (bottom row of panels and blue lines in right hand figure). The 2D maps shown are deviations from leaf-area averages. The leaf-area average values of transpiration rates are given at the top of each panel. The three columns (left-to-right) and line styles (solid, dashed, dotted) show the respective dependencies on the **external gas concentration of CO**_**2**_: low (100 ppm), medium (400 ppm), and high concentration (800 ppm). Other model parameter values can be found in Supplementary Information I. Note that the transpiration rates are represented as −*F*_*T*_ for easy reading.

**Figure 6:**
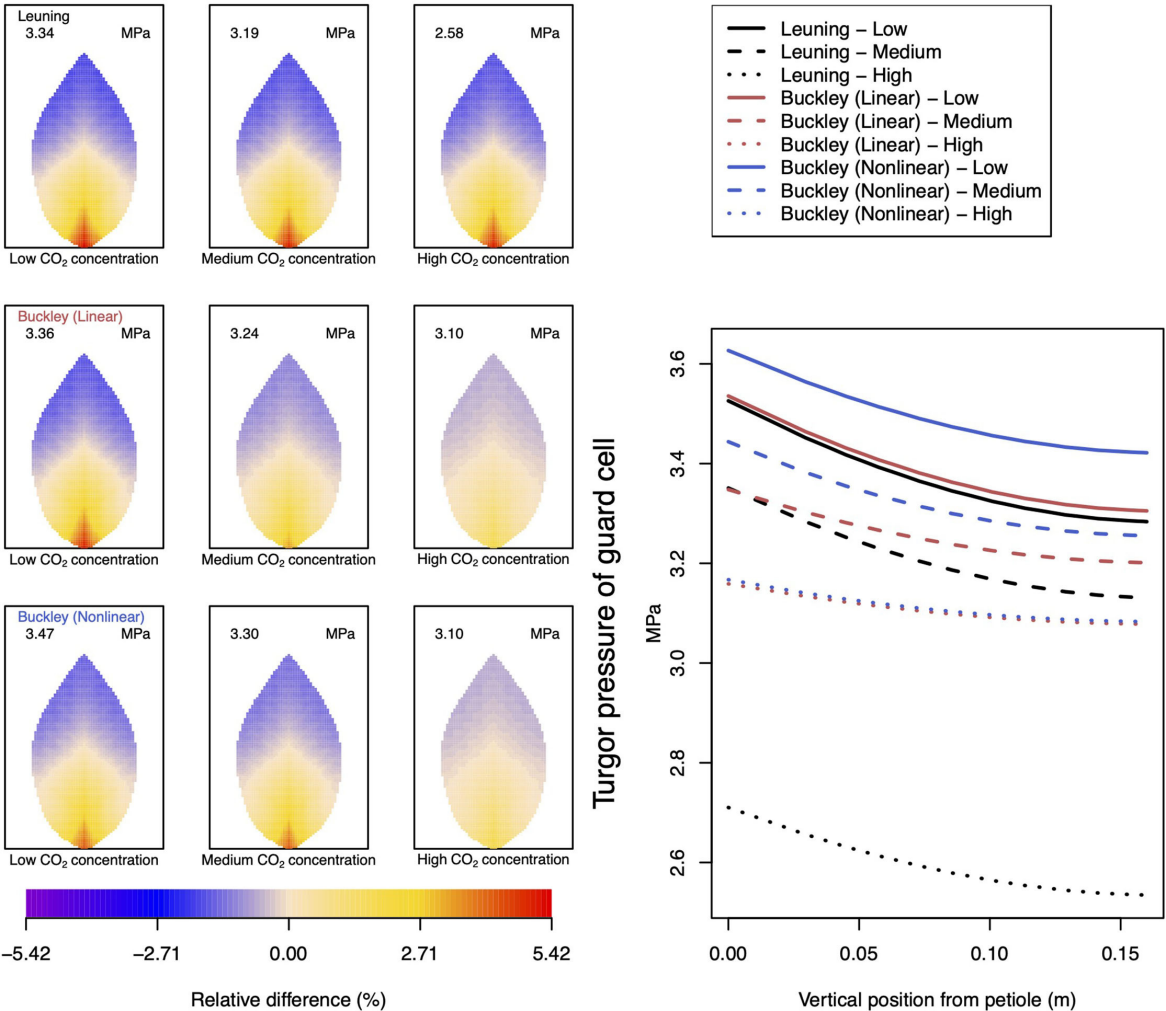
A comparison of predictions of guard cell turgor pressure distributed over the leaf according to the three stomatal conductance models of Leuning (1995) (top row of panels and black lines in right hand figure), linear (Buckley et al., 2003) (middle row of panels and red lines in the right hand figure), and nonlinear (Buckley et al., 2003) (bottom row of panels and blue lines in right hand figure). The 2D maps shown are deviations from leaf-area averages. The leaf-area average values of transpiration rates are given at the top of each panel. The three columns (left-to-right) and line styles (solid, dashed, dotted) show the respective dependencies on the **external gas concentration of CO**_**2**_: low (100 ppm), medium (400 ppm), and high concentration (800 ppm). Other model parameter values can be found in Supplementary Information I.

In Figures 4–**6**, we begin to address the principal task of investigating the difference in predictions of photosynthesis and transpiration by the three chosen models of stomatal conductance. In the present calculations, we have kept fixed the leaf size and shape as well as the vein architecture. In these calculations, we have also incorporated tapering of first and second order veins.

To be clear, in the matrix of nine panel figures of Figure 4, we show the deviation from the leaf-area average, steady-state photosynthesis production. The deviations are quoted as relative differences


$$\begin{array}{l} \frac{P-P_{av}}{P_{av}}\times 100\%. \end{array}$$

The leaf-area average values themselves are provided at the top of each panel in the figures. The right-most line graphs show the actual numerical rates of photosynthesis associated locally for points along the central, main (mid-rib) vein.

Analogous related results of leaf transpiration shown in Figure 5 are given in precisely the same format as are those of guard cell turgor pressure in Figure 6. This is repeated in all later figures.

Figure 5 documents the dependence of transpiration on environmental CO_2_ concentration. The panel figures of both Figures 4, 5 convey first the clear message that the hydromechanical model of Buckley et al. (2003), whether linear or nonlinear, depicts a spatial variation across the leaf. Through the connection between photosynthesis and transpiration rate, the transpiration results shown in Figure 5 show the same patterns and trends as those of photosynthesis activity (the qualitative appearance of the nine leaf area distributions in Figure 5 is virtually identical to the corresponding panels in Figure 4).

At medium to high levels of CO_2_, the difference between the predictions of photosynthesis using the original and modified models is one of degree only. At low CO_2_, however, the modified, nonlinear (Buckley et al., 2003) model, based on the empirical relation Equation (9), predicts a distribution of photosynthesis rate that is the converse of the original model's prediction. In the latter case, the photosynthesis rate is higher in the petiole region and lower in the outer leaf region, while with the nonlinear functional dependence we find that the photosynthesis production is less near the petiole. In absolute terms, however, the difference between the two photosynthesis rate predictions is not large and diminishes as the level of external CO_2_ rises. The transpiration rates compare similarly: the two predictions of transpiration close to and far from the petiole at low CO_2_ are transposed but are quantitatively similar at medium and high CO_2_. Indeed, the two models converge as CO_2_ rises. The actual values found along the main vein (bottom right hand panels of Figures 4, 5) confirm the qualitative differences at low CO_2_ and quantitative agreement as CO_2_ rises.

In stark contrast to these two versions of the Buckley et al. (2003) model, the model of Leuning (1995) does not exhibit any significant spatial variation. On the other hand, the leaf-area average values of photosynthesis are comparable, and only slightly less comparable are the leaf-area average predictions of transpiration. More significantly, the model's predictions show that both properties respond consistently to CO_2_ changes.

Not surprisingly, of the three models, only the linear (Buckley et al., 2003) model's prediction of the spatial distribution of both photosynthesis and transpiration mimics the spatial pattern of guard cell turgor pressure (Figure 6).

#### 3.2.2. Dependence on Relative Humidity

**Figure 7:**
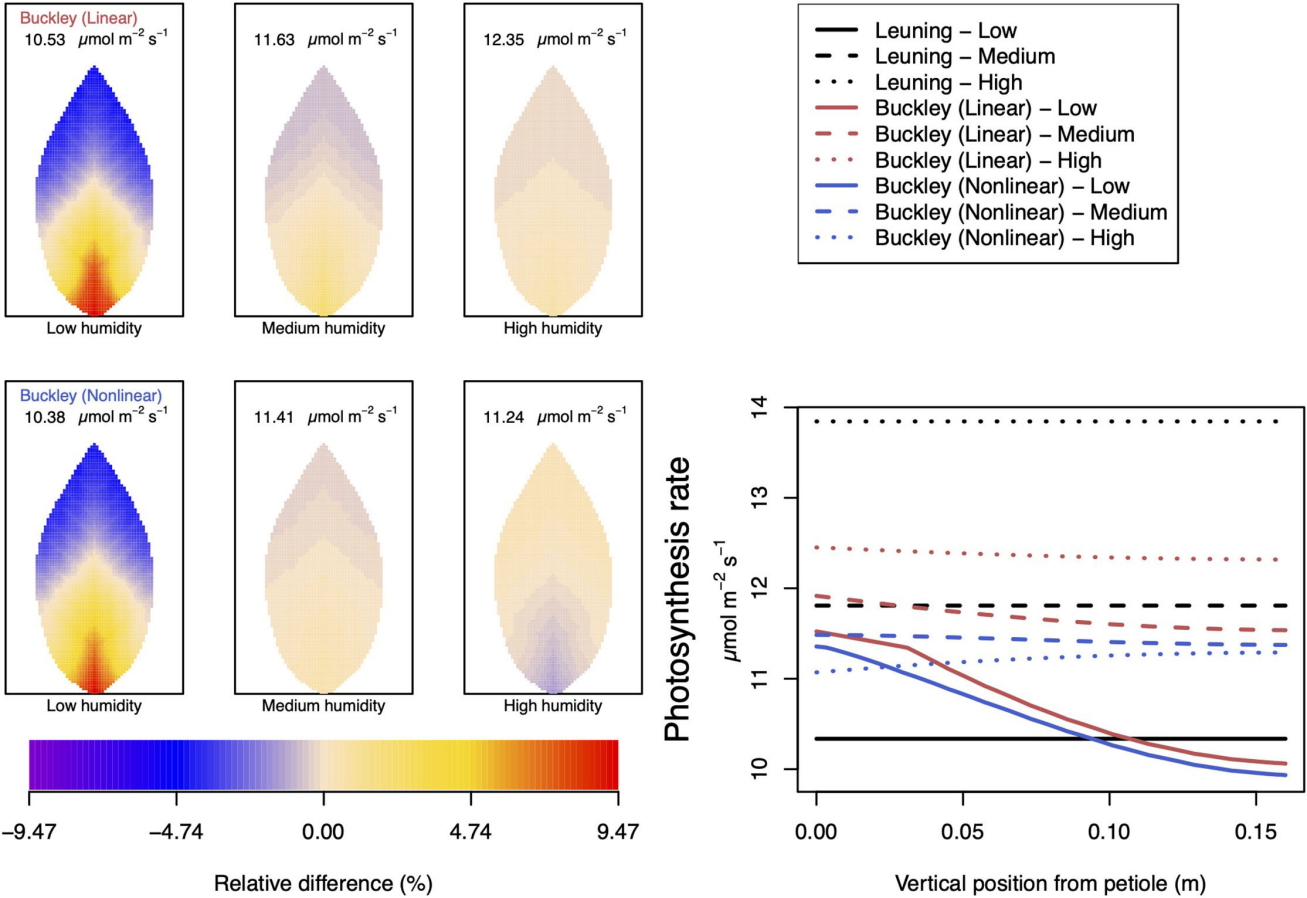
A comparison of predictions of photosynthetic activity distributed over the leaf according to the three stomatal conductance models of Leuning (1995) (black lines in right hand figure), linear (Buckley et al., 2003) (top row of panels and red lines in the right hand figure), and nonlinear (Buckley et al., 2003) (bottom row of panels and blue lines in right hand figure). The 2D maps shown are deviations from leaf-area averages. The leaf-area average values of photosynthesis rates are given at the top of each panel. The three columns (left-to-right) and line styles (solid, dashed, dotted) show the respective dependencies on the **atmospheric relative humidity**: low (10%), medium (50%), and high humidity (90%). For the Leuning (1995) model, the uniform predictions equal the leaf-area photosynthesis averages of 10.34, 11.81, and 13.84 μmol m^−2^ s^−1^ under low, medium, and high humidity, respectively (2D heat maps are not shown). Other model parameter values can be found in Supplementary Information I.

**Figure 8:**
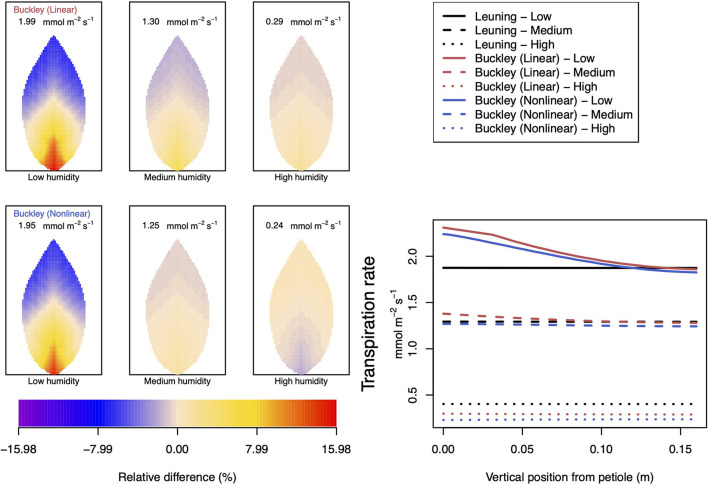
A comparison of predictions of transpiration rate (−*F*_*T*_) distributed over the leaf according to the three stomatal conductance models of Leuning (1995) (black lines in right hand figure), linear (Buckley et al., 2003) (top row of panels and red lines in the right hand figure), and nonlinear (Buckley et al., 2003) (bottom row of panels and blue lines in right hand figure). The 2D maps shown are deviations from leaf-area averages. The leaf-area average values of transpiration rates are given at the top of each panel. The three columns (left-to-right) and line styles (solid, dashed, and dotted) show the respective dependencies on the **atmospheric relative humidity**: low (10%), medium (50%), and high humidity (90%). For the Leuning (1995) model, the uniform predictions equal the leaf-area transpiration averages of 1.87, 1.29, and 0.4 mmol m^−2^ s^−1^ under low, medium, and high humidity, respectively (2D heat maps are not shown). Other model parameter values can be found in Supplementary Information I. Note that the transpiration rates are represented as −*F*_*T*_ for easy reading.

Somewhat different to their dependence on CO_2_, the two (Buckley et al., 2003) stomatal conductance models, incorporated in our leaf model, show quite similar patterns at low humidity and trend similarly in response to increasing environmental relative humidity (Figures 7, 8; corresponding turgor pressure plots can be found in Supplementary Figure SI.4). In general, both photosynthetic production and transpiration distributions over the leaf predicted by the empirical model of Leuning (1995) again show little or no spatial variation under any of the three humidity conditions tested (and hence are not shown), while leaf area averages remain consistent with those of the other models (values are given in the figure caption). As in the case of CO_2_, we see that the linear (Buckley et al., 2003) model predicts a spatial variation that is most pronounced at low humidity (dry) conditions, and whose amplitude progressively wanes as atmospheric humidity increases; the spatial variation all but vanishes at saturation. Here, we find converse spatial patterns of both photosynthesis and transpiration predicted by the two (Buckley et al., 2003) models, but this time at high humidity. On a leaf-area average basis, under low to medium humidity conditions the quantitative trends of all three models are similar: photosynthesis increases with increased humidity, while transpiration decreases, and the spatial variations of both diminish as the atmosphere becomes more humid. At the high humidity end, the nonlinear (Buckley et al., 2003) model's photosynthesis response is to decrease, cumulating overall in a nonmonotonic dependence on humidity. In comparison, the linear (Buckley et al., 2003) model responds monotonically in terms of both photosynthesis and transpiration.

#### 3.2.3. Dependence on Leaf Irradiance

**Figure 9:**
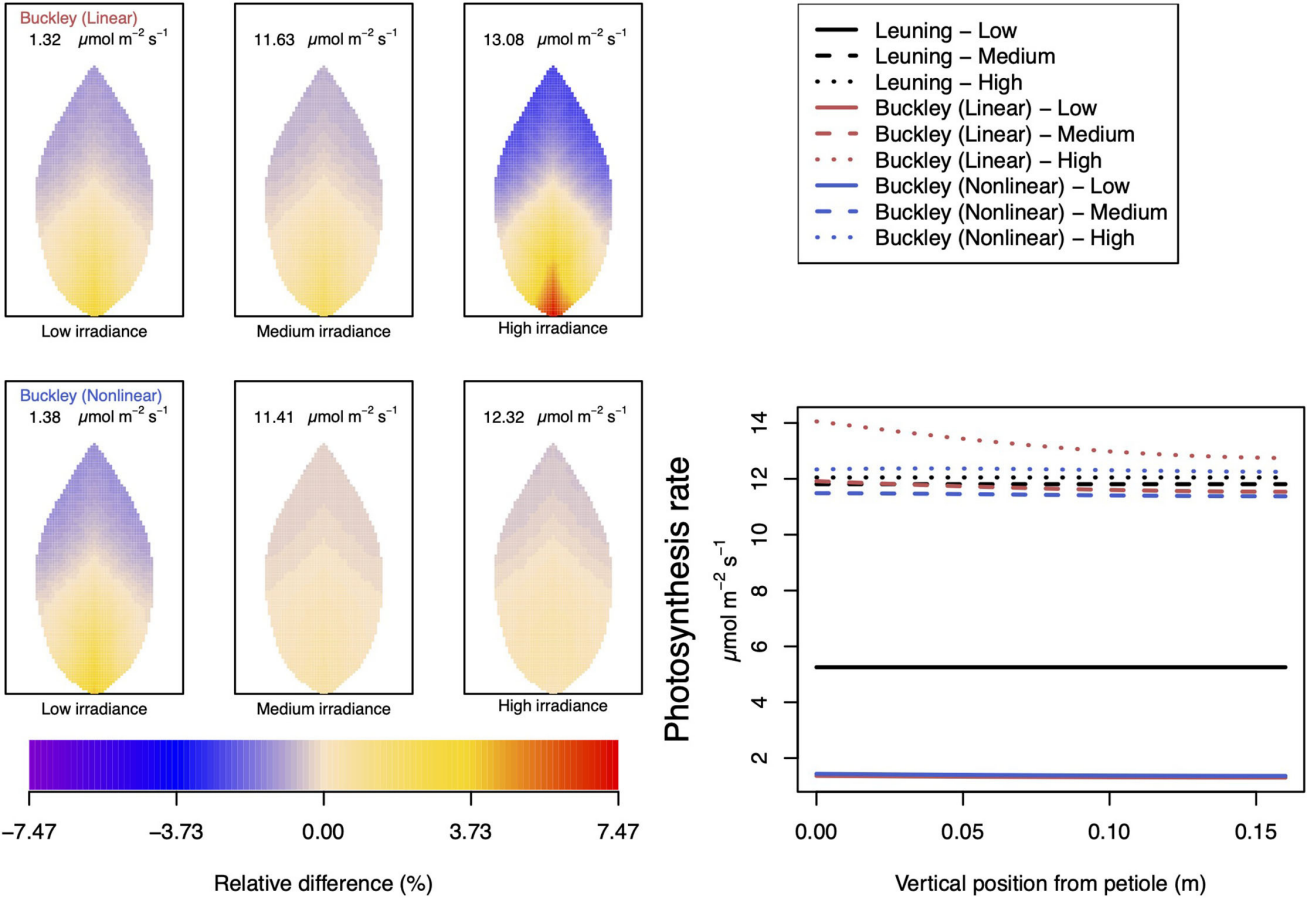
A comparison of predictions of photosynthetic activity distributed over the leaf according to the three stomatal conductance models of Leuning (1995) (black lines in right hand figure), linear (Buckley et al., 2003) (top row of panels and red lines in the right hand figure), and nonlinear (Buckley et al., 2003) (bottom row of panels and blue lines in right hand figure). The 2D maps shown are deviations from leaf-area averages. The leaf-area average values of photosynthesis rates are given at the top of each panel. The three columns (left-to-right) and line styles (solid, dashed, dotted) show the respective dependencies on **light intensity**: low (200 μmol m^−2^ s^−1^); medium (500 μmol m^−2^ s^−1^); and high irradiance (1,000 μmol m^−2^ s^−1^). For the Leuning (1995) model, the uniform predictions equal the leaf-area photosynthesis averages of 5.25, 11.81, and 12.05 μmol m^−2^ s^−1^ under low, medium, and high intensity, respectively (2D heat maps are not shown). Other model parameter values can be found in Supplementary Information I.

**Figure 10:**
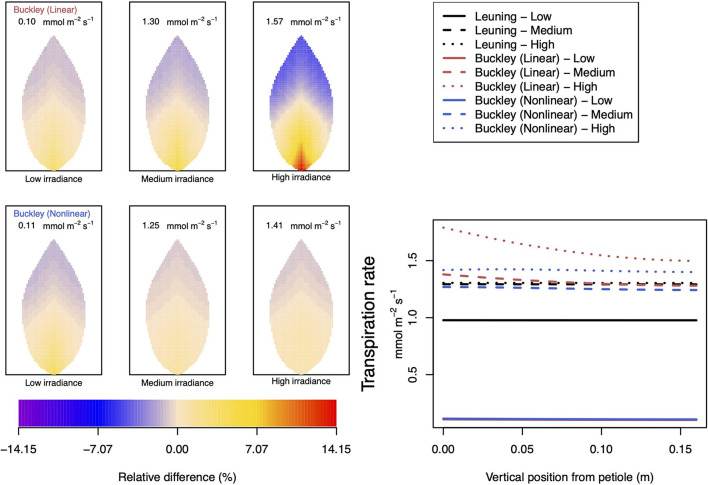
A comparison of predictions of transpiration rate (−*F*_*T*_) distributed over the leaf according to the three stomatal conductance models of Leuning (1995) (black lines in right hand figure), linear (Buckley et al., 2003) (top row of panels and red lines in the right hand figure), and nonlinear (Buckley et al., 2003) (bottom row of panels and blue lines in right hand figure). The 2D maps shown are deviations from leaf-area averages. The leaf-area average values of transpiration rates are given at the top of each panel. The three columns (left-to-right) and line styles (solid, dashed, and dotted) show the respective dependencies on the **light intensity**: low (200 μmol m^−2^ s^−1^); medium (500 μmol m^−2^ s^−1^); and high irradiance (1,000 μmol m^−2^ s^−1^). For the Leuning (1995) model, the uniform predictions equal the leaf-area transpiration averages of 0.98, 1.29, and 1.30 mmol m^−2^ s^−1^ under low, medium, and high intensity, respectively (2D heat maps are not shown). Other model parameter values can be found in Supplementary Information I. Note that the transpiration rates are represented as −*F*_*T*_ for easy reading.

In our investigation into the system's dependence on the light intensity, we considered three different values: low (200 μmol m^−2^ s^−1^); medium (500 μmol m^−2^ s^−1^); and high irradiance (1,000 μmol m^−2^ s^−1^). The model predictions of photosynthesis and transpiration are shown in Figures 9, 10 (the corresponding panel of guard cell turgor pressure can be found in Supplementary Figure SI.5). The models' trends of both leaf properties contrast yet again compared with their respective responses to CO_2_ and humidity. To begin with, both (Buckley et al., 2003) models actually predict consistent 2D patterns under all light intensity conditions: higher activity near the petiole, and lower activity near the leaf tip, and their leaf area average values are also quite similar. Second, the linear (Buckley et al., 2003) model exhibits the greatest spatial variation at high irradiance which, contrastingly, is present under low CO_2_ and low humidity conditions. On the other hand, the nonlinear model displays the greatest spatial variation under low irradiance, although this is relative to a low area-averaged value. From the perspective of the quantitative values along the main vein, presented in the graphs in the panel figure on the right hand side of Figures 9, 10, the linear and nonlinear models predict similar magnitudes at low intensity, but progressively diverge as light intensity increases, while remaining qualitatively consistent overall.

#### 3.2.4. Dependence on Temperature

**Figure 11:**
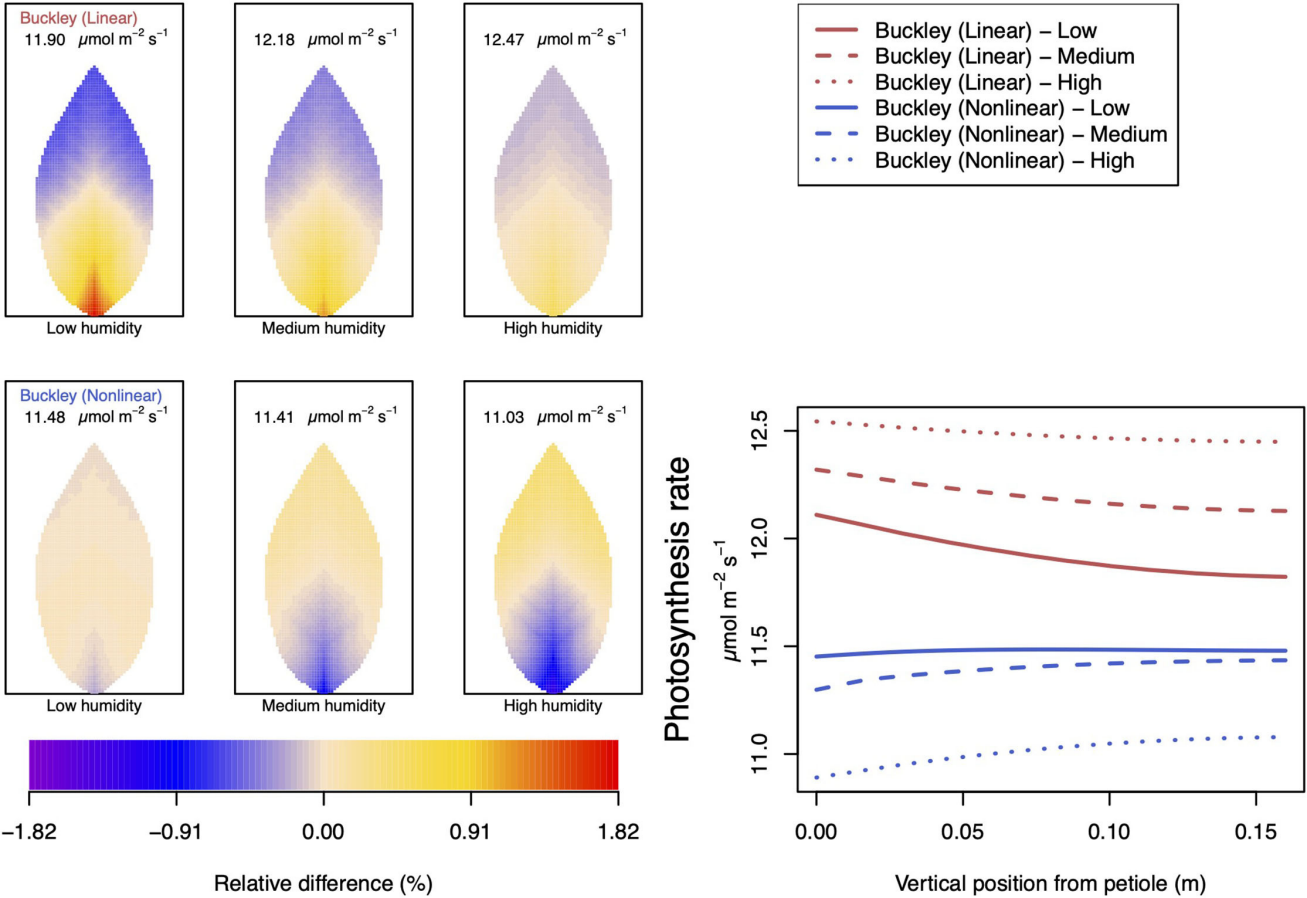
A comparison of model predictions of photosynthetic activity distributed over the leaf for different values of **relative humidity**. Details as in Figure 7, except **for a temperature of 10°C (283.15 K)**. In this case, the uniform/leaf-area average predictions using the Leuning (1995) stomatal model are 12.47, 13.29, and 14.23 μmol m^−2^ s^−1^ under low, medium, and high humidity, respectively (2D heat maps are not shown).

**Figure 12:**
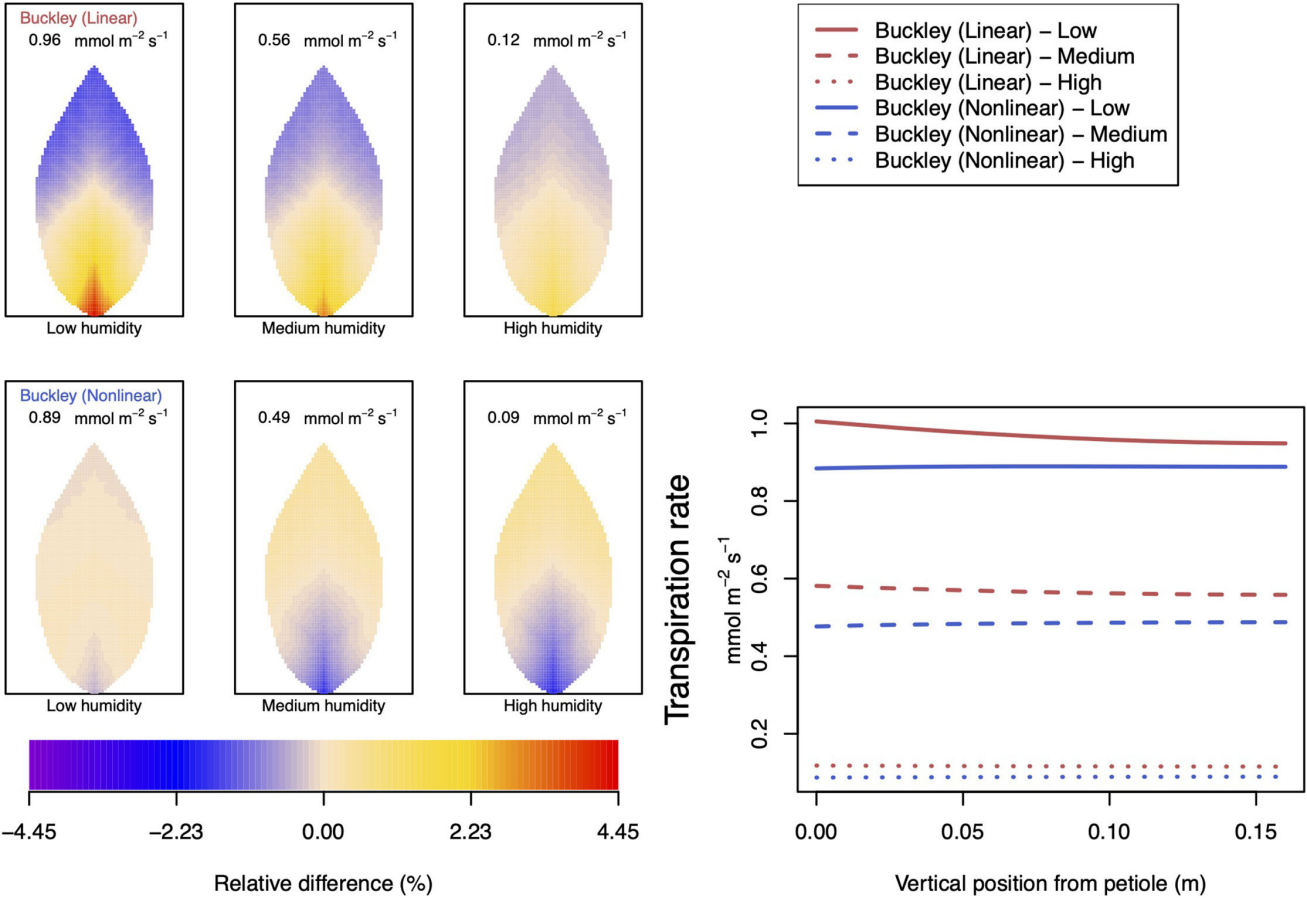
A comparison of model predictions of transpiration rates (−*F*_*T*_) distributed over the leaf for different values of **relative humidity**. Details as in Figure 8, except **for a temperature of 10°C (283.15 K)**. In this case, the uniform/leaf-area average predictions using the Leuning (1995) stomatal model are 1.03, 0.69, and 0.18 mmol m^−2^ s^−1^ under low, medium, and high humidity, respectively (2D heat maps are not shown). Note that the transpiration rates are represented as −*F*_*T*_ for easy reading.

The final external condition we consider is that of temperature. Our simulations, varying CO_2_ concentration, humidity, and irradiance, were repeated but this time under the condition of lower temperature (down from 25 to 10°C). The results of these repeat calculations are shown, respectively, in Figures 11, 12 and Supplementary Figures SI.6–SI.12. Compared with the corresponding results in Figures 4, 5, 7–10, the most striking difference found at a lower temperature, highlighted here in Figures 11, 12, occurs with the dependence on humidity. This is discussed further below and in Section 4. The temperature has a larger quantitative influence on transpiration, with the leaf-area averages reduced by slightly more than a factor of 2. The magnitude of photosynthesis is only slightly affected. Whether this is a second order effect of a reduced water flow (i.e., reduced transpiration), or whether the small magnitude change is a direct effect of a lower temperature is not clear.

In the case of the dependence on CO_2_ (Supplementary Figures SI.6–SI.8), the interesting feature arises with the nonlinear (Buckley et al., 2003) stomatal conductance model for which a reduced temperature *qualitatively* alters both photosynthesis and transpiration when the availability of CO_2_ is low to medium. Activity in the region near the petiole is much reduced compared with the activities at the higher temperature. The linear model does not show any qualitative difference, and both models more or less agree at high CO_2_ (as they did at 25°C).

As mentioned, quantitative and qualitative differences in the predictions of transpiration and photosynthesis ensue between the two temperature cases under different conditions of humidity: Figures 11, 12. The most striking qualitative difference, in both photosynthesis and transpiration, appears again with the nonlinear (Buckley et al., 2003) model: at low humidity (and relative to the leaf average) the petiole region changes from being more active at 25°C to being less active at 10°C than the leaf average and vice versa for the outer leaf region. While the average activity decreases as humidity increases, the spatial gradient becomes steeper. It is also interesting that although the linear and nonlinear model predictions are arguably similar at 25°C, there are clear divergences at the lower temperature under all three humidity conditions. A more marked effect is that although the linear (Buckley et al., 2003) model continues to predict a monotonic *increase* in the photosynthesis rate with humidity, the nonlinear model now predicts a monotonic *decrease* in activity with increased humidity. Both models continue to predict a monotonic decrease in the transpiration rate as the humidity of the atmosphere rises.

Finally, as we vary light intensity at 10°C (Supplementary Figures SI.10–SI.12), the behavior of the two models, i.e., relative to their respective behavior at 25°C, as well as relative to each other at 10°C, is different yet again. With the linear (Buckley et al., 2003) stomatal conductance model the qualitative response at each level of irradiance remains the same at 10°C as at the higher temperature, while with the nonlinear stomatal conductance model the qualitative behavior is opposite to its predictions at 25°C and converse also to the predictions of the linear model (at either temperature). This time the greatest deviation occurs at medium and high light intensity. At low levels of light, the predictions of the two models are similar (as they were at 25°C).

### 3.3. Dependence on Leaf Shape

Different shapes of leaves, longer/narrower leaves (aspect ratio *l*:*w* = 1:0.28 in Supplementary Figure SI.21 and shorter/wider leaves (aspect ratio *l*:*w* = 1:0.63 in Supplementary Figure SI.22), of the *same total area*, do not lead to dramatically altered values or responses to changes in external conditions, as measured either on the basis of leaf-area average or according to values along the main vein (quoted in the figures). Nevertheless, from Supplementary Figures SI.19, SI.24, we see the remarkable consequence that is induced by nonuniform intrinsic properties *on* the spatial patterns of extrinsic (or acquired) properties (such as photosynthesis) in leaves of different aspect ratios. The distinctly different spatial patterns exhibited in Supplementary Figures SI.19, SI.24 are representatives of the patterns found with other distributed properties (such as transpiration and guard cell turgor pressure) under these or other external conditions. Refer to Supplementary Figures SI.13–SI.30.

## 4. Discussion

### 4.1. General Review of Simulation Results

The very first and overarching feature of note is that although the external conditions are uniform over the spatial extent of the leaf, the leaf response is spatially nonuniform. We found this to be the case in Sakurai and Miklavcic (2021) for the xylem hydraulic pressure, which reported results of a simpler model. However, the feature remains true with our extended and more detailed model even for other physiological activities in the leaf such as photosynthesis and transpiration, assuming the validity of either (Buckley et al., 2003) stomatal conductance model.

One of the largest differences between the Leuning and the Buckley models is that the former does not include a dependence on the turgor pressures of guard cells and epidermal cells in the function of stomatal conductance. The non-uniform distribution patterns predicted by the Buckley model, on the other hand, are a direct result of the latter's consideration of turgor pressure in the operation of stomatal aperture. The gradients of guard cell turgor pressure and those of epidermal cell turgor pressures are in turn derived from gradients of the hydraulic pressure existing across the leaf, which are needed to provide sufficient transpiration even at the top of the leaf. Hence, because the model of Leuning (1995) does not include these factors it produces a uniform distribution pattern of photosynthesis and transpiration irrespective of the existence of turgor pressure gradients.

In the above figures, we have covered a wide range of environmental conditions, which added considerable depth and dimension to the set of leaf activities portrayed in Figure 3. Many of the findings, based on either (Buckley et al., 2003) model, are physically reasonable: high humidity results in low transpiration, high CO_2_, or high irradiance gives rise to higher photosynthetic production (and consequential increased water movement). In the majority of cases, but not always, the difference between the linear and nonlinear (Buckley et al., 2003) models is simply one of degree (i.e., magnitude). To be precise, at 25°C, we have seen that when activity is low (e.g., low transpiration under high humidity) there appear a few qualitative differences. On the other hand, at the lower temperature of 10°C, the two representations of stomatal aperture on the turgor pressures of the epidermis and guard cells, predict results that differ more often both quantitatively and qualitatively. Unfortunately, it is not always easy to understand the reasons for such differences. Linear models, such as the original, linear version of the Buckley et al. (2003) model of stomatal aperture, being proportional to the difference in the turgor pressures of guard cell and subsidiary epidermal cells, are intuitively appealing as well as being easier to understand, and their predictions easier to anticipate. As a general observation, linear models predict monotonic responses to changes in external stimuli. This has been our experience with the linear (Buckley et al., 2003) model. With nonlinear functions, on the other hand, it is possible for non-monotonic dependencies to arise. In this prosaic respect, it is not surprising that our calculations based on the nonlinear version of the Buckley et al. (2003) model produce non-monotonic responses. At 25°C, only two instances of non-monotonic responses arise epidermal turgor pressure with increasing CO_2_ concentration (refer to Supplementary Figure SI.36), and photosynthesis with increasing humidity. In both instances, there were no corresponding, non-monotonic trends in the other variables. It may be an important fact that in both instances, the external conditions were such as to generate a low level of activity. For the non-monotonic responses for photosynthesis with increasing humidity, one possible mechanism would be high humidity due to the epidermal back pressure effect. Because of the nonlinear relationship between stomatal aperture and the turgor pressure, the effect of humidity on guard cell turgor saturates before the effect on epidermal turgor if the guard cell turgor pressure is too high. A possible result is a stomatal closure with increasing guard cell turgor pressure (Supplementary Figures SI.34, SI.35). On the other hand, at 10°C more instances of nonlinear and occasionally unexpected behavior arise, such as the opposite trend of photosynthetic response to increased humidity in Figure 11 (also refer to Supplementary Figures SI.32, SI.33 for an alternative comparison of the effect of temperature). These cases do not always correspond to cases of low activity, such as photosynthesis (and transpiration) under high light irradiance (Supplementary Figures SI.10, SI.11).

The sum total of examples of contrary behavior perhaps indicates a fundamental limitation of the physical assumptions adopted in the model(s). Unfortunately, without further theoretical analysis, we cannot address this issue. At the very least, these examples highlight the importance of giving proper consideration to an accurate description of the mechanisms controlling stomatal conductance. Whether or not one adopts the view that stomatal conductance should be included in the definition of leaf conductance, or considered a separate property in order to distinguish liquid movement from gaseous movement, it is clear that stomatal control is an important mechanism that steers the total movement of water (in any phase) from the petiole to the atmosphere. Optimistically, the cases for which the linear and nonlinear models deviate qualitatively may suggest environmental conditions under which experiments can be performed to shed further light on the question.

In terms of influencing the leaf's response to different environmental states, we found that the shape of a leaf does not play a significant role. This, true for any given stomatal conductance model, is readily confirmed by appeal to either the leaf-area averaged values of quantities (mean values quoted in the matrices of panels of 2D distributions) or the actual values along the main vein. The same, however, cannot be said of the impact a nonuniform response has on leaves of different shapes. In the long/narrow leaf (aspect ratio of *l*:*w* = 19.2:5.39 = 1:0.28) as with our standard leaf (*l*:*w* = 16:6.47 = 1:0.40), the nonuniform activity divides the leaf effectively longitudinally, with the upper half (containing the leaf tip) generally being the region of lower activity and the lower half (including the petiole) being the region of higher activity. This was also found in our earlier model (Sakurai and Miklavcic, 2021). However, in contrast to our earlier study with the simpler model, in short/wide leaves (*l*:*w* = 12.8:8.09 = 1:63), the nonuniform activity divides the leaf laterally, with two lower activity lobes on the outer edges, on either side of higher activity, the central region encompassing the main vein and petiole. The different pattern is no doubt due to the different hydraulic distances from the petiole to those respective regions. Although we have not modeled the case, it is not difficult to imagine the patterns of activity that would be found in each blade of a palmately compound leaf (such as a clover leaf (Trifolium *repens*)), as a smaller scale reproduction, per leaf blade, of what is shown here.

### 4.2. Dependence on CO_2_ Concentration

It is noteworthy and perhaps surprising that using any of the three stomatal conductance models, our leaf hydraulic model predicts a comparable degree of spatial variability in guard cell turgor pressure (e.g., top row to bottom row in Figure 6). However, there is a distinct difference in the responses to increases in CO_2_. While it is clear from the color maps (and values along the main vein) that the stomatal conductance model of Leuning (1995) shows a consistent negative gradient across the leaf (measured radially from the petiole to the leaf perimeter, the turgor pressure becomes less positive), the linear stomatal conductance model of Buckley et al. (2003) predicts a somewhat shallower (negative) gradient depending on CO_2_. That is, in the latter model, the turgor pressure appears to become more homogeneous over the leaf under increasing CO_2_ concentration. This is reproduced when the linear stomatal aperture model is replaced by the non-linear, empirical model proposed by Franks et al. (1998). The results are shown in the nine panels on the left of Figure 6. It is perhaps more significant, however, to note that the turgor pressure distribution predicted by the linear (Buckley et al., 2003) model is consistent with the corresponding predictive trends in photosynthesis and (particularly) transpiration. In contrast, the turgor pressure predictions found using the Leuning (1995) model are *at odds* with the respective photosynthesis and transpiration predictions. This internal inconsistency of the Leuning (1995) model demonstrates the necessity of including some explicit dependence of stomatal conductance on guard cell turgor pressure. On the other hand, we have demonstrated the precise relationship between stomatal conductance (stomatal aperture) and guard cell turgor pressure—more specifically the guard cell and epidermal cell turgor pressures—is also important. The two versions of the Buckley et al. (2003) model can sometimes show qualitative differences, as well as quantitative differences in some leaf properties (e.g., photosynthesis and transpiration under low CO_2_ or high humidity). However, we point out that at least at 25°C a low level of activity may cast a shadow of doubt on the validity of specific differences.

In terms of photosynthesis itself, with a low level of raw photosynthetic material (i.e., with a low level of externally available CO_2_), the rate of production is greatly reduced in both linear and nonlinear versions of the Buckley et al. (2003) models, but in the former case, it is more highly discriminated across the leaf. This spatial discrimination shows a slightly elevated rate of production near the petiole, where the rate of transpiration is also greatest. In contrast, the empirical model of Leuning (1995), while similarly showing an overall low level of photosynthetic activity under low CO_2_ conditions, does not exhibit any significant spatial variation over the leaf. This is quantified by the actual photosynthetic production rates predicted along the main vein (refer to the right hand panel of Figure 4) where clearly no variation is evident as predicted by the model.

As the level of available CO_2_ rises, the photosynthetic activity increases consistently. It is particularly significant that, according to both versions of the Buckley et al. (2003) model, in parallel with an overall increased level of photosynthesis production with available CO_2_, the predicted degree of variation over the leaf *diminishes*. This trend continues until the external environment becomes saturated with CO_2_, which results in a high level of photosynthetic activity accompanied by a low level of spatial variation. Based on this trend, one may argue that under highly saturated conditions all three models become comparable in the sense that they each point to a homogeneous response over the leaf. However, quantitatively, both versions of the Buckley et al. (2003) model diverge significantly from the Leuning (1995) model in this high CO_2_ limit.

### 4.3. Dependence on Relative Humidity

We remark that according to Equation (6) of the Buckley et al. (2003) model, the photosynthesis rate shifts from a dependence on a Rubisco-limited carboxylation rate, *W*_*c*_ to a RuBP-limited carboxylation rate, *W*_*j*_. Under low humidity conditions (refer to Supplementary Figure SI.31), this has the effect of inducing gradient discontinuities in photosynthetic activity, transpiration and guard cell turgor pressure along the main vein (indeed along all radial lines emerging from the petiole), as demonstrated by the line graphs of Figures 8–10. This tendency is a consequence of the characteristics of the original model by Farquhar et al. (1980). However, it is unknown how sharp the transition actually is, even in a single chloroplast, but this discontinuity would be smoothed when the whole leaf average is calculated under the transition under different humidity, CO_2_, or irradiance conditions. This may be a point of interest when discussing the experimental data about the response to changes in humidity or CO_2_.

The linear hydraulic model of Buckley et al. (2003) qualitatively predicts turgor pressures that show similar qualitative responses to humidity: there is the greatest spatial variation at low humidity and least at high humidity. However, in contrast with this qualitative perspective, the whole leaf hydraulic model predicts a different quantitative response in guard cell turgor pressure to increasing relative humidity, depending on the choice of the stomatal conductance model. With the Buckley et al. (2003) and Leuning (1995) models, the turgor pressure increases monotonically with increasing humidity, although to different degrees. The three responses are reflected in the curves in the right hand panel of Supplementary Figure SI.4.

Under changes in relative humidity, from low to high and specifically at 25°C, we first observe that both linear and nonlinear (Buckley et al., 2003) models predict increased turgor pressures of both guard and epidermis cells as humidity is increased. (In both the CO_2_ and irradiance dependencies these turgor pressures change in opposite directions.) Nevertheless, the net effect is an overall monotonic decrease in transpiration. The latter property shows close agreement between the linear and nonlinear conductance models. The only residual disparity is a reversed spatial distribution of transpiration under high humidity conditions; the linear model has higher transpiration near the petiole and lower transpiration in the outer region, while the nonlinear model predicts the converse. However, at high humidity, the absolute levels of transpiration are quite low, as is the degree of variation over the leaf.

As usual, the qualitative character of the photosynthesis rate distributions follows closely the spatial patterns of transpiration. On a quantitative level, however, photosynthesis rates predicted by the two models at 25°C are in reasonable agreement only at low humidity and deviate increasingly as humidity increases. Moreover, the nonlinear model again shows a non-monotonic response to increases in atmospheric H_2_O. At 10°C, this non-monotonic response becomes a fully monotonic response in the opposite direction to that predicted by the linear model.

### 4.4. Dependence on Irradiation

Photosynthesis production, percent-wise, is dramatically increased from a level at low irradiation to one at medium irradiation, with the linear (Buckley et al., 2003) model predicting slightly lower production at low irradiation, but slightly higher production at medium to high irradiation, than is predicted with the nonlinear (Buckley et al., 2003) model. At least both show the same increasing trend. The linear model shows the greatest spatial variation at high CO_2_, while the nonlinear model shows none. It is unclear why the spatial variation is so high. Needless to say, the transpiration patterns display identical behavior.

At variance to the response to CO_2_ changes, both the guard cell and epidermal cell turgor pressures show greater spatial variation at high irradiance than at low irradiance. Moreover, the quantitative dependence is reversed: epidermal turgor pressure *decreases*, while guard cell turgor pressure *increases* leading to larger stomatal apertures and increased transpiration. Another contrasting element (compared with the dependence with CO_2_), is that the two models are in general agreement at low irradiance and less in agreement at high irradiance.

### 4.5. Dependence on Temperature—Additional Comments

In our model, we assume thermal equilibration of leaf tissues with the external environment. Although the many chemical reactions taking place in the leaf tissue are possibly and probably temperature dependent, as are also some physical and biological properties (e.g., cell membrane elastic moduli, vein conductivities, and V_cmax_), the temperature dependencies of the parameters representing these have not been quantified. As such we have limited ability to examine properly the temperature dependence of the model. Currently, temperature enters explicitly (and linearly) through the osmotic pressure terms (and related factors) and the difference of the evaporative gradient (when the relative humidity is the same, the evaporative gradient under low temperature is largely high relative to that under normal temperature). These account for the significant changes to the magnitudes of transpiration and only minor magnitude effects on, say, photosynthesis. Nevertheless, the non-linear (Buckley et al., 2003) model is more influenced qualitatively than quantitatively, while in the case of the linear (Buckley et al., 2003) model it is the converse. These features reflect the properties of the linear vs. nonlinear models.

## 5. Conclusion

The original stomatal conductance model of Buckley et al. (2003) is based on a coherent interplay of physical processes that take their cues from the local conditions of atmospheric humidity, the local concentration of CO_2_, and the degree of irradiation. More specifically, the model explicitly involves the local guard cell turgor pressure in its predictions of transpiration and photosynthesis activity. While refinements of this model have been developed over the years, it still represents a standard model of local stomatal behavior. Nevertheless, as is true of any model, certain basic and intuitive assumptions may undergo improvement as our knowledge of the biophysics of stoma increases. An example of this is the experimental finding of Franks et al. (1998) that the stomatal aperture's dependence on guard cell and epidermal cell turgor pressures is more complex than simply a proportionality to the difference of these pressures. All the same, the model is useful when implemented in an extended 2D hydraulic model of a leaf. Its full significance is revealed. Since CO_2_, humidity, and light intensity are arguably uniform as measured on the scale of a leaf, the key physical differential is the local value of turgor pressure of the guard cell, which ultimately controls the stomatal pore opening and hence determines stomatal conductance (and hence leaf transpiration). With regard to the linear vs. nonlinear dependence of stomatal aperture on guard cell and epidermal cell turgor pressures, both generally predict the same behavior in terms of the response to changes in external conditions and predict very similar 2D distribution patterns of many properties. There are, however, exceptions which, at 25°C, are predominantly associated with low levels of activity (e.g., photosynthesis at low CO_2_, and transpiration at high humidity). If one overlooks these few exceptions, it would appear that the linear (Buckley et al., 2003) model—with stomatal aperture dependent explicitly on the difference in cell turgor pressures—provides an adequate representation of stomatal control (at least from a quantitative modeling perspective). At the lower temperature of 10°C, there are distinct prediction differences, a feature which may be a focal point for future experiments to gain a greater understanding of stomatal behavior and its dependence on guard cell and epidermal cell turgor pressures.

The picture emerging from our extended leaf hydraulic model, incorporating the Buckley et al. (2003) stomatal conductance model, is that the leaf functions of transpiration and photosynthesis are not uniformly operative even when the external conditions are. This is the case even in a completely healthy leaf. While the purpose of our study was not to compare the respective predictive capabilities of the three stomatal models, the differences, as well as the similarities, in the predictions based on the models do give greater insight into their respective properties and inferences into leaf behavior. Our simulations suggest that the gradient pattern of the hydraulic pressure needed to maintain transpiration from the whole leaf is derived from the gradient patterns of guard cell and epidermal cell turgor pressures, which leads to complex spatial distribution patterns of transpiration and photosynthesis as determined by the mechanical stomatal model. Within our knowledge, there has not been an experimental study addressing the relationship between the distribution pattern of transpiration rate (or photosynthesis rate) across a leaf and the environmental conditions to complement the present theoretical study. However, such an experimental study could contribute to a deeper understanding of the mechanisms of stomatal control in plants, particularly in collaboration with the simulation investigation presented here.

Although our model findings and conclusions are valid within the limits set by a passive transport model—active cell membrane transport mechanisms (such as those orchestrated by aquaporin transporters) are still to be added—the results do have implications for a leaf's sensitivity to damage, or other interruption of flow. Near the symmetry centre of the petiole, a leaf is more sensitive to a local interruption but is less sensitive in the outer regions of the leaf. According to either Figures 5, 8, or 10, the low transpiration region extends over a larger area of the leaf compared with the high transpiration region near the petiole. Consequently, arguing purely from an area perspective, a disruption confined to a small element of the area in the outer region of the leaf will not greatly alter the total transpiration (or photosynthesis production), compared with the level of overall disruption that would result from a localized disruption confined to an equally-sized area of the high transpiration (or photosynthesis) region near the symmetry centre of the petiole. For example, a leaf undergoing senescence on its perimeter will generally continue to transpire (or photosynthesize) to the same overall degree.

Our model represents an improvement or refinement of the model published earlier by us (Sakurai and Miklavcic, 2021). However, even this model—still within the confines of passive transport—is no more than an approximation to a real leaf's operation. In future study, we shall distinguish between apoplastic and symplastic pathways within the mesophyll, the bundle sheath, and the bundle sheath extension. As discussed earlier in this article, the parameters we have employed, here, are considered concatenations of the transport parameters that represent the apoplastic and symplastic pathways. Consequently, their magnitudes will not necessarily agree with any experimentally determined values of either apoplastic or symplastic pathways. In order to correspond with an experimental measurement, these pathways need to be distinguished. It is also important to distinguish these pathways in order to properly incorporate active membrane transport mechanisms in the symplastic pathway.

In future study, we shall also consider fully the role of the bundle sheath extension, which not only extends the path between the vascular bundle and the epidermis, but adds the physical feature of a lignified layer on the surface exposed to intercellular air spaces (aeroles)—the equivalent of the root's Casparian strip—preventing or at least limiting the loss of water molecules to the aeroles, and simultaneously the passage CO_2_ molecules into the mesophyll.

Finally, in this study, we have represented the phloem, the xylem, the mesophyll, and the epidermis as four 2D networks that are coupled in a linear chain, and we have focused on the movement of water from the stem (petiole) to the atmosphere *via* stomata under the control of one of three different stomatal conductance models. In future consideration, we shall pay equal attention to the movement of sucrose (the photosynthetic product) taking place within these tissue structures. However, this more complete treatment of transport of sucrose (as well as water) requires a modification of the linear coupled network structure to that of a nonlinear coupling of the networks, with the phloem also directly connected to the mesophyll. This will allow the phloem to explicitly receive (and transport) photosynthetic products out through the petiole to the stem. This latter process has only been implicitly assumed in the present study in order to simplify our study of stomatal conductance.

## Data Availability Statement

The original contributions presented in the study are included in the article/Supplementary Material, further inquiries can be directed to the corresponding author/s.

## Author Contributions

GS and SM were equal contributors to the model development and design of simulation experiments, analysis of results, and the drafting of the manuscript. GS wrote the numerical code, performed the simulations, and prepared final figures. Both authors contributed to the article and approved the submitted version.

## Funding

This project was supported by the Australian Research Council (Discovery Project grant DP200103168) and by JSPS KAKENHI grants (16H06296 and 19H03085).

## Conflict of Interest

The authors declare that the research was conducted in the absence of any commercial or financial relationships that could be construed as a potential conflict of interest.

## Publisher's Note

All claims expressed in this article are solely those of the authors and do not necessarily represent those of their affiliated organizations, or those of the publisher, the editors and the reviewers. Any product that may be evaluated in this article, or claim that may be made by its manufacturer, is not guaranteed or endorsed by the publisher.
