# Supplementary material for: A Whole Leaf Comparative Study of Stomatal Conductance Models
Source: Front Plant Sci. 2022 Apr 11;13:766975. doi: 10.3389/fpls.2022.766975 (PMC9036488; doi:10.3389/fpls.2022.766975)
Supplement: Supplementary file 1 [file Data_Sheet_1.PDF]

Supplementary Information I: A whole leaf comparative study of stomatal conductance models

1 MODEL DESCRIPTION

1.1 Water flow network

The model we propose here is an integrated model of the leaf model developed by our previous study (Sakurai and Miklavcic, 2021) and stomatal conductance models proposed by Leuning (1995), Buckley et al. (2003), or the modified version of Buckley et al. (2003) in which a non-linear function is used for stomatal aperture using the euqation in Franks et al. (1998). In our previous model, the complex leaf vein architecture of a leaf was presented as two layers of two-dimensional networks of xylem and phloem, in which each layer was structured with multiple nodes. In the present model, two additional layers are added: the mesophyll layer and the epidermis layer. The mesophyll layer connects to the xylem network and the epidermal layer connects to the mesophyll layer at the corresponding nodes. In the present model, these four layers represent the structure of a whole leaf. Note that, although the model has four layers, the model does not imitate the three-dimensional structure of the leaf. Therefore, the water status of the mesophyll and epidermal layers in the model represent the representative values of them at each nodes (Figure S1). In the following equations, the superscripts with roman symbols do not indicate exponentiating but represent the position in the leaf (for example, “mes” means mesophyll layer and “gu” means guard cells).

In the present paper, we used the same vein structure and leaf shape as in our previous work (Sakurai and Miklavcic, 2021) for the xylem and the phloem network. This originally corresponds to the Laurel leaf shape and vein structure adopted and studied in the work of Cochard et al. (2004). The first-order vein depicts the main vein connected directly to the petiole at the base of the leaf. The second-order veins, shown in purple in Figure S1, branch directly off from the first-order vein at regular intervals. Higher-order veins (third, fourth, and fifth-order veins) are arranged on and aligned with a rectangular lattice of nodal points (blue dots in Figure S1). The third-order veins are distributed, both vertically and horizontally, at a frequency of every six nodal points. The two narrowest veins occur at the same frequency, but with their respective networks displaced by one rectangular grid unit so as to appear alternately in a similar rectangular pattern (Figure S1). The angle of the second veins is $45°$. The intervals of the second-order veins alternate between 14.70 mm and 12.60 mm (see, Sakurai and Miklavcic (2021) for the explanation of how the intervals were decided) except for the interval between the petiole and the first second-order vein.

Figure S2 shows the leaf shape used in the present study. The shape is the same as that used in the previous study (Sakurai and Miklavcic, 2021). The 0.20 m $\times$ 0.10 m surface is constructed by 65 $\times$ 130 nodes and the vertical and horizontal distance between adjacent nodes is about 1.50 mm. Among the 65 $\times$ 130 nodes, 3138 nodes construct the shape of the leaf (green area in Figure S2). in order to avoid an excessive calculation time (the present model needs 10 to 20 times longer calculation period than the model of Sakurai and Miklavcic (2021)), the resolution is coarser than that adopted in the previous model. Despite this the results adopting similar conditions are almost the same among the models with the two different resolutions.

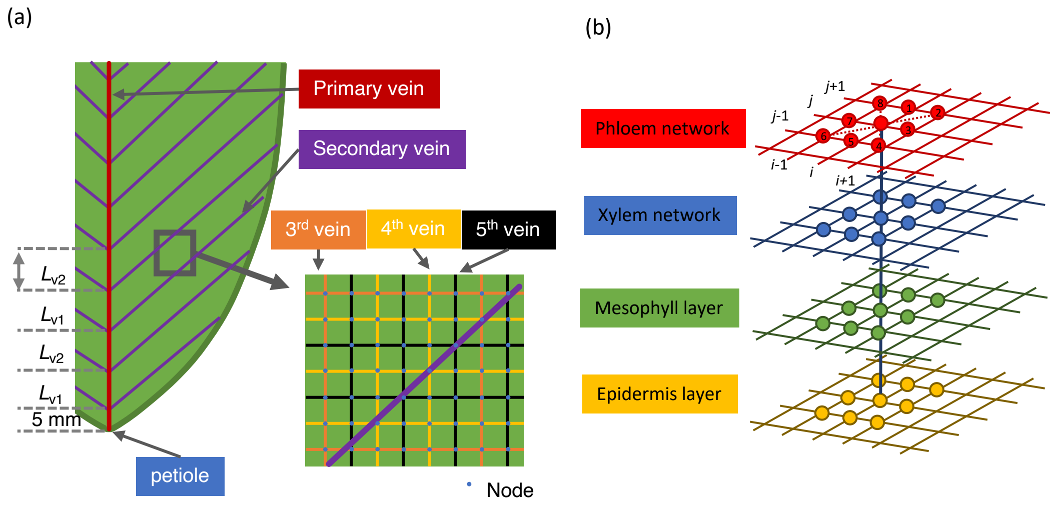

Figure S1. (a) Schematic diagram of the vein architecture of the model leaf. The vein structure in the model is represented by five types of veins. L_{v1} and L_{v2} are the intervals of the second veins (14.70 mm and 12.60 mm, respectively) and they aline alternately. (b) Schematic diagram of the network of the phloem, xylem, mesophyll, and epidermis. The water can flow from a single node (i, j) to the nearest and the next nearest neighbor nodes within each network (these are numbered d = 1 to d = 8). The black conduit connects a node in each layer to its corresponding partner node(s) in the neighbor layer(s). In our computations, the nodes are numbered in a grid-like fashion: $(i, j) = (1, 1) \ldots (N, M)$, as indicated by the numbering in the phloem network.

1.2 Conservation constraint of zero net water flux

In the main text, the layers were numbered $k = 1, \ldots, 4$ in order to present as concise a description. However, in this Supplementary Information, the description is written in full. We use the symbols “ph”, “xyl”, “mes”, and “epi”, for phloem, xylem, mesophyll, and epidermis, respectively, so as to improve readability of the model description.

The transport of fluid via leaf vein in the xylem and the phloem systems is modeled as a system of equations, founded on Darcy’s law of plug flow (Batchelor, 1967): $\mu \mathbf{u} = -\iota \nabla p$, expressing the fact that in the conduit between two consecutive nodes the fluid velocity, $\mathbf{u}$, is proportional to the pressure gradient across the conduit joining those nodes, with the vein conductance (ι/μ) being the coefficient of proportionality; here ι is a fluid permeability (m^2) and μ is the fluid viscosity (Pa.s), and the negative sign is consistent with flow from a point of high to a point of low hydraulic pressure.

At the (i, j)th xylem node (hereafter abbreviated to ij, for $i = 1, \ldots, N, j = 1, \ldots, M$), we apply the conservation constraint of zero net water flux out of the xylem node. The conservation constraint asserts that the sum of the fluxes in the eight xylem conduit directions ($\sum_{d=1}^{8} F_{ij-d}^{\mathrm{xyl}}$), plus the flux from the xylem node to its corresponding phloem node, $F_{ij-\mathrm{px}}^{\mathrm{xyl}}$ and mesophyll node, $F_{ij-\mathrm{xm}}^{\mathrm{xyl}}$, is equal to zero.

$$\sum_{d=1}^{8} F_{ij-d}^{\mathrm{xyl}} + F_{ij-\mathrm{px}}^{\mathrm{xyl}} + F_{ij-\mathrm{xm}}^{\mathrm{xyl}} = 0, \qquad \text{(S1)}$$

$$(i, j) = (1, 1), \ldots, (N, M).$$

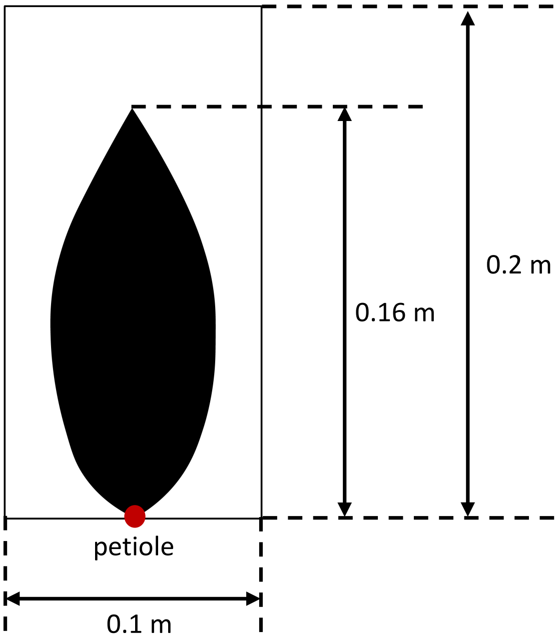

Figure S2. Leaf shape used for the present study.

In the same fashion, we have, at the ijth phloem node, the zero sum of water fluxes in the eight lateral directions, $\{F^{\text{ph}}_{ij-1}, \ldots, F^{\text{ph}}_{ij-8}\}$, as well as the cross flow from the phloem node to its corresponding xylem node, $F^{\text{ph}}_{ij-\text{px}}$ $(= -F^{\text{xyl}}_{ij-\text{px}})$:

$$\sum_{d=1}^{8} F^{\text{ph}}_{ij-d} + F^{\text{ph}}_{ij-\text{px}} = 0, \qquad (i,j) = (1,1), \ldots, (N,M). \tag{S2}$$

In the mesophyll layer, we assume water flow via plasmodesmata connecting a given mesophyll cell with neighbor cells in the lateral direction, $\{F^{\text{mes}}_{ij-1}, \ldots, F^{\text{mes}}_{ij-8}\}$. Therefore, at the ijth mesophyll node, the sum of the fluxes in the eight conduit directions ($\sum_{d=1}^{8} F^{\text{mes}}_{ij-d}$), plus transpiration ($F^{\text{mes}}_{ij-\text{T}}$) and the flux from the mesophyll node to its corresponding xylem node, $F^{\text{mes}}_{ij-\text{xm}}$ $(= -F^{\text{xyl}}_{ij-\text{xm}})$ and epidermal node, $F^{\text{mes}}_{ij-\text{me}}$, is equal to zero.

$$\sum_{d=1}^{8} F^{\text{mes}}_{ij-d} + F^{\text{mes}}_{ij-\text{xm}} + F^{\text{mes}}_{ij-\text{me}} + F^{\text{mes}}_{ij-\text{T}} = 0, \qquad (i,j) = (1,1), \ldots, (N,M). \tag{S3}$$

Similarly, we have, at the ijth epidermal node, the zero sum of water fluxes in the eight lateral directions, $\sum_{d=1}^{8} F^{\text{epi}}_{ij-d}$, as well as transpiration ($F^{\text{epi}}_{ij-\text{T}}$) and the cross flow from the epidermal node

to its corresponding mesophyll node, $F^{\text{epi}}_{ij-\text{me}}$ $(= -F^{\text{mes}}_{ij-\text{me}})$:

$$\sum_{d=1}^{8} F^{\text{epi}}_{ij-d} + F^{\text{epi}}_{ij-\text{me}} + F^{\text{epi}}_{ij-\text{T}} = 0, \qquad (i,j) = (1,1),\ldots,(N,M). \tag{S4}$$

1.3 Water flow in xylem and phloem

Invoking a discrete, cross-sectional area-integral version of Darcy's law, the flux between two consecutive xylem nodes is determined from the relation

$$F^{\text{xyl}}_{ij-d} = K^{\text{xyl}}_{ij-d}(p^{\text{xyl}}_{ij} - p^{\text{xyl}}_{d}), \qquad (i,j) = (1,1),\ldots,(N,M), \tag{S5}$$

where

$$K^{\text{xyl}}_{ij-d} = \frac{\iota_{ij-d} A^{\text{xyl}}_{ij-d}}{\mu\, l_{ij-d}} = \frac{\kappa^{\text{xyl}}_{ij-d}}{l_{ij-d}} \qquad (i,j) = (1,1),\ldots,(N,M), \tag{S6}$$

is the conductance of the xylem conduit between those nodes in terms of its cross-sectional area, A^{xly}_{ij-d}, length, l_{ij-d}, permeability ι_{ij-d}, and fluid viscosity, μ. $\kappa^{\text{xyl}}_{ij-d}$ is the conductance of the unit length xylem conduit.

In the phloem, fluid motion is driven by hydraulic and osmotic pressure influences. Darcy's law must then be modified to include an osmotic pressure contribution resulting from a concentration difference:

$$\Pi^{\text{ph}} = -\alpha^{\text{ph}} RT \Delta C^{\text{ph}}, \tag{S7}$$

where R is the universal gas constant ($R = 8.314 \times 10^{-6}$ MPa m^3 mol^{-1} K^{-1}), T is temperature (in degrees K), and C^{ph} is the local concentration of solute (only sucrose is considered in phloem) inside the sieve tube. The parameter α^{ph} is a proportionality constant.

In discrete form, the cross-sectional area-integral of the flux in the phloem becomes

$$F^{\text{ph}}_{ij-d} = K^{\text{ph}}_{ij-d}\left((p^{\text{ph}}_{ij} - p^{\text{ph}}_{d}) - \sigma^{\text{ph}}_{ij-d} RT (C^{\text{ph}}_{ij} - C^{\text{ph}}_{d})\right), \qquad (i,j) = (1,1),\ldots,(N,M), \tag{S8}$$

where, in direct analogy with the xylem case, F^{ph}_{ij-d} is the (signed) fluid volume flux *from* phloem node ij *to* the phloem node connected to it in the direction d (one of eight neighbors). The position dependent parameter $\sigma^{\text{ph}}_{ij-d}$ is called the reflection coefficient (Katchalsky and Curran, 1965; Foster and Miklavcic, 2014, 2016; Sakurai and Miklavcic, 2021). In Eq (S8), $\sigma^{\text{ph}}_{ij-d} = 0$ since the solute movement is assumed not to be impeded (Kramer and Boyer, 1995). K^{ph}_{ij-d} is the fluid conductance of the phloem conduit between

the ijth node and its neighbor in the direction d:

$$K^{\mathrm{ph}}_{ij-d} = \frac{\iota_{ij-d}A^{\mathrm{ph}}_{ij-d}}{\mu\, l_{ij-d}} = \frac{\kappa^{\mathrm{ph}}_{ij-d}}{l_{ij-d}} \qquad (i,j) = (1,1),\ldots,(N,M), \tag{S9}$$

where $\kappa^{\mathrm{ph}}_{ij-d}$ is the conductance of the unit length phloem conduit. When calculating the cross-sectional area, A^{xyl}_{ij-d}, we assumed a circular cylinder with a radius of r, and that the relationship between the cross-sectional areas of the xylem and the phloem were proportional for simplicity. Then,

$$A^{\mathrm{ph}}_{ij-d} = \zeta A^{\mathrm{xyl}}_{ij-d} = \zeta\pi r^2_{ij-d}, \qquad (i,j) = (1,1),\ldots,(N,M). \tag{S10}$$

Assuming that the distances between consecutive xylem nodes are the same as those between consecutive phloem nodes, the relationship between the xylem conductance and the phloem conductance is simply described as,

$$K^{\mathrm{ph}}_{ij-d} = \frac{\kappa^{\mathrm{ph}}_{ij-d}}{l_{ij-d}} = \zeta\frac{\kappa^{\mathrm{xyl}}_{ij-d}}{l_{ij-d}} = \zeta K^{\mathrm{xyl}}_{ij-d}. \qquad (i,j) = (1,1),\ldots,(N,M). \tag{S11}$$

The (signed) volume flux of water *from* the xylem *to* the phloem, $F^{\mathrm{xyl}}_{ij-\mathrm{px}}$, (or vice versa) is determined by the difference between the hydraulic pressure at the xylem node and the sum of hydraulic pressure and osmotic pressure at the corresponding phloem node, multiplied by the conductance of the conduit linking those nodes. This is expressed by the relation:

$$\begin{aligned} F^{\mathrm{xyl}}_{ij-\mathrm{px}} &= -F^{\mathrm{ph}}_{ij-\mathrm{px}} \\ &= K_{ij-\mathrm{px}}\left(p^{\mathrm{xyl}}_{ij} - (p^{\mathrm{ph}}_{ij} - \sigma_{ij-\mathrm{px}}RTC^{\mathrm{ph}}_{ij})\right), \\ &\qquad (i,j) = (1,1),\ldots,(N,M). \end{aligned} \tag{S12}$$

where $K_{ij-\mathrm{px}}$ is the conductance of the route between the two nodes, and $\sigma_{ij-\mathrm{px}}$ is the reflection coefficient for this pathway, which is here set to unity (*i.e.*, $\sigma_{ij-\mathrm{px}} = 1$). From this, we see that even if the hydraulic pressures in the xylem and phloem networks are equal, the presence of a solution in the phloem will drive fluid from the xylem to the phloem network.

1.4 Water flow in mesophyll and epidermis

In the mesophyll and epidermis, fluid motion is driven not only by hydraulic pressure but also by osmotic pressure:

$$\begin{aligned} F^{\mathrm{mes}}_{ij-d} &= K^{\mathrm{mes}}_{ij-d}\big((p^{\mathrm{mes}}_{ij} - p^{\mathrm{mes}}_{d}) \\ &\quad -\sigma^{\mathrm{mes}}_{ij-d}RT(C^{\mathrm{mes}}_{ij} - C^{\mathrm{mes}}_{d})\big), \\ &\qquad (i,j) = (1,1),\ldots,(N,M), \end{aligned} \tag{S13}$$

$$\begin{aligned} F_{ij-d}^{\text{epi}} &= K_{ij-d}^{\text{epi}} \Big((p_{ij}^{\text{epi}} - p_d^{\text{epi}}) \\ &\quad -\sigma_{ij-d}^{\text{epi}} RT (C_{ij}^{\text{epi}} - C_d^{\text{epi}}) \Big) , \qquad \text{(S14)} \\ &\qquad (i,j) = (1,1), \ldots, (N,M), \end{aligned}$$

where F_{ij-d}^{mes} and F_{ij-d}^{epi} are the (signed) fluid volume flux *from* mesophyll (or epidermal) node ij *to* the mesophyll (or epidermal) node connected to it in the direction d (one of eight neighbors). K_{ij-d}^{mes} and K_{ij-d}^{epi} are the fluid conductances of the mesophyll and epidermis between the ijth node and its neighbor in the direction d. C_{ij}^{mes} and C_{ij}^{epi} are the solute concentrations in the mesophyll and epidermal cells, respectively. For convenience we assume that the water movement within the mesophyll or epidermis is predominantly via plasmodesmata. Hence, $\sigma_{ij-d}^{\text{mes}} = \sigma_{ij-d}^{\text{epi}} = 0$. The fluid conductances of the mesophyll and epidermis is the reciprocal of the distance between the ijth node and its neighbor. Then,

$$\begin{aligned} K_{ij-d}^{\text{mes}} &= \frac{\kappa_{ij}^{\text{mes}}}{l_{ij-d}}, \qquad \text{(S15)} \\ K_{ij-d}^{\text{epi}} &= \frac{\kappa_{ij}^{\text{epi}}}{l_{ij-d}}, \qquad \text{(S16)} \\ &\qquad (i,j) = (1,1), \ldots, (N,M), \end{aligned}$$

The (signed) volume flux of water *from* the xylem *to* the mesophyll, $F_{ij-\text{xm}}^{\text{xyl}}$ $(= F_{ij-\text{xm}}^{\text{mes}})$, is determined by the difference between the hydraulic pressure at the xylem node and the sum of hydraulic pressure and osmotic pressure at the corresponding mesophyll node, multiplied by the conductance of the conduit linking those nodes. This is expressed by the relation:

$$\begin{aligned} F_{ij-\text{xm}}^{\text{xyl}} &= -F_{ij-\text{xm}}^{\text{mes}} \\ &= K_{ij-\text{xm}} \Big(p_{ij}^{\text{xyl}} - (p_{ij}^{\text{mes}} - \sigma_{ij-\text{xm}} RT C_{ij}^{\text{mes}}) \Big) , \qquad \text{(S17)} \\ &\qquad (i,j) = (1,1), \ldots, (N,M), \end{aligned}$$

where $K_{ij-\text{xm}}$ is the conductance between the two nodes and $\sigma_{ij-\text{xm}}$ is a reflection coefficient for this pathway ($\sigma_{ij-\text{xm}} = 1$).

The (signed) volume flux of water *from* the mesophyll *to* the epidermis, $F_{ij-\text{me}}^{\text{mes}}$ $(= -F_{ij-\text{me}}^{\text{epi}})$, is determined by the difference of the total pressure between the mesophyll and the epidermis as here indicated:

$$\begin{aligned} F_{ij-\text{me}}^{\text{mes}} &= -F_{ij-\text{me}}^{\text{epi}}, \\ &= K_{ij-\text{me}} \big((p_{ij}^{\text{mes}} - \sigma_{ij-\text{me}} RT C_{ij}^{\text{mes}}) \\ &\quad -(p_{ij}^{\text{epi}} - \sigma_{ij-\text{me}} RT C_{ij}^{\text{epi}}) \big) , \qquad \text{(S18)} \\ &\qquad (i,j) = (1,1), \ldots, (N,M), \end{aligned}$$

where $K_{ij-\text{me}}$ is the conductance between the two nodes and $\sigma_{ij-\text{me}}$ is a reflection coefficient for this pathway ($\sigma_{ij-\text{me}} = 1$).

As mentioned in Eqs (S3) and (S4), we assume transpiration takes place from both the mesophyll region and the epidermal region. For simplicity, $F^{\text{mes}}_{ij-\text{T}}$ and $F^{\text{epi}}_{ij-\text{T}}$ are determined as being proportional to the total transpiration rate from the stomatal node ij ($F^{\text{s}}_{ij-\text{T}}$). For each node, the total transpiration rate is represented as the product of conductance ($g^{\text{all}}_{ij-\text{w}}$) and leaf-to-boundary layer H_2O mole fraction gradient (D^{s}_{ij}). Therefore, $F^{\text{mes}}_{ij-\text{T}}$ and $F^{\text{epi}}_{ij-\text{T}}$ are given as,

$$\begin{aligned} F^{\text{mes}}_{ij-\text{T}} &= e^{\text{mes}} F^{\text{s}}_{ij-\text{T}} \\ &= -e^{\text{mes}}(a_{ij} g^{\text{all}}_{ij-\text{w}} D^{\text{s}}_{ij}) \\ & \qquad (i,j) = (1,1), \ldots, (N,M). \end{aligned} \tag{S19}$$

$$\begin{aligned} F^{\text{epi}}_{ij-\text{T}} &= e^{\text{epi}} F^{\text{s}}_{ij-\text{T}} \\ &= -e^{\text{epi}}(a_{ij} g^{\text{all}}_{ij-\text{w}} D^{\text{s}}_{ij}) \\ & \qquad (i,j) = (1,1), \ldots, (N,M). \end{aligned} \tag{S20}$$

where a_{ij} is the 2D grid area assigned to that node ij and e^{mes} and e^{epi} are proportional constants ($e^{\text{mes}} + e^{\text{epi}} = 1$).

1.5 Sucrose transport

In direct analogy with the water fluxes, we assume a conservation of sucrose fluxes. Namely, we specify that the sum of all sucrose fluxes into and out from a given node in the eight lateral directions ($S_{ij-1}, \ldots, S_{ij-8}$), plus a contribution from sucrose loading into the sieve tube ($S_{ij-\text{L}}$) should be equal to zero:

$$\sum_{d=1}^{8} S_{ij-d} + S_{ij-\text{L}} = 0. \tag{S21}$$

In the above equation, the sucrose loading into the sieve tube ($S_{ij-\text{L}}$) is calculated as $S_{ij-\text{L}} = \Lambda_{ij} a_{ij}$, where Λ_{ij} is the local sucrose loading rate per unit area and we assume that this rate is related to the photosynthesis rate P_{ij}. Then,

$$\begin{aligned} \Lambda_{ij} &= \eta P_{ij}, \\ & \qquad (i,j) = (1,1), \ldots, (N,M), \end{aligned} \tag{S22}$$

where η is proportion of the loading rate relative to the photosynthesis rate.

Sucrose flow is driven by a combination of convection, which is proportional to the total pressure difference between neighboring phloem nodes, and diffusion, which depends on the sucrose concentration

difference between those same two phloem vein nodes. This sum is expressed by the equation,

$$\begin{aligned} S_{ij-d} &= (1-\sigma^{\text{ph}}_{ij-d})v_\mu F^{\text{ph}}_{ij-d}C^{\text{ph}}_{ij} + D_{\text{su}}A^{\text{ph}}_{ij-d}\frac{C^{\text{ph}}_{ij}-C^{\text{ph}}_{d}}{l_{ij-d}}, \\ &= (1-\sigma^{\text{ph}}_{ij-d})v_\mu F^{\text{ph}}_{ij-d}C^{\text{ph}}_{ij} + G^{\text{ph}}_{ij-d}\left(C^{\text{ph}}_{ij}-C^{\text{ph}}_{d}\right), \\ &(i,j)=(1,1),\dots,(N,M), \end{aligned} \tag{S23}$$

where

$$G^{ph}_{ij-d} = D_{\text{su}}\frac{A^{\text{ph}}_{ij-d}}{l_{ij-d}}. \tag{S24}$$

S_{ij-d} is defined as the mass flux *from* node ij *to* the neighbor node in the d direction, F^{ph}_{ij-d} is the corresponding volume flow of water, σ_{ij-d} is the reflection coefficient (here, $\sigma^{\text{ph}}_{ij-d}=0$), v_μ is the volume of water per mmol ($\mathrm{m}^3\,\mathrm{mmol}^{-1}$), and D_{su} is the free diffusion sucrose diffusivity ($D_{\text{su}} = 5.22\times10^{-10}$ $\mathrm{m}^2\,\mathrm{s}^{-1}$).

1.6 Solute concentration in mesophyll and epidermis

We assumed that the solute molar contents in the mesophyll cells and the epidermal cells do not change according to the cell condition such as the volume and the turgor pressure of the cells for simplicity, which means that the solute concentration is determined by the volume of the water inside the cells (w^{mes}_{ij} and w^{epi}_{ij}):

$$C^{\text{mes}}_{ij} = C^{\text{mes}}_0\frac{w^{\text{mes}}_0}{w^{\text{mes}}_{ij}}, \tag{S25}$$

$$C^{\text{epi}}_{ij} = C^{\text{epi}}_0\frac{w^{\text{epi}}_0}{w^{\text{epi}}_{ij}}, \tag{S26}$$

$$(i,j)=(1,1),\dots,(N,M),$$

where C^{mes}_0 and C^{epi}_0 are the solute concentrations at the maximum volume of water in the cells (w^{mes}_0 and w^{epi}_0). If we assume a linear relationship between the change in ratio of the water volumes in the cells and the resulting changes in the turgor pressure, we can describe the water volume at the pressure p^{mes}_{ij} or p^{epi}_{ij} as:

$$w^{\text{mes}}_{ij} = w^{\text{mes}}_0\frac{p^{\text{mes}}_{ij}-p^{\text{mes}}_{\text{max}}+\epsilon^{\text{mes}}}{\epsilon^{\text{mes}}}, \tag{S27}$$

$$w^{\text{epi}}_{ij} = w^{\text{epi}}_0\frac{p^{\text{epi}}_{ij}-p^{\text{epi}}_{\text{max}}+\epsilon^{\text{epi}}}{\epsilon^{\text{epi}}}, \tag{S28}$$

$$(i,j)=(1,1),\dots,(N,M),$$

where $p^{\text{mes}}_{\text{max}}$ and $p^{\text{epi}}_{\text{max}}$ are the maximum turgor pressure at the maximum volume of water in the cells. ϵ^{mes} and ϵ^{epi} are the bulk modulus of elasticity of the cell, which can be defined by:

$$\epsilon = \frac{\Delta p}{\Delta v / v_0}, \tag{S29}$$

where v_0 is the maximum volume of the cell and Δv indicates the volume change when the turgor pressure of the cell is changed with Δp (Jones, 2014). Therefore, we can rewrite Eqs (S25) and (S26) as,

$$\begin{aligned} C^{\text{mes}}_{ij} &= C^{\text{mes}}_0 \frac{\epsilon^{\text{mes}}}{p^{\text{mes}}_{ij} - p^{\text{mes}}_{\text{max}} + \epsilon^{\text{mes}}}, && \text{(S30)} \\ C^{\text{epi}}_{ij} &= C^{\text{epi}}_0 \frac{\epsilon^{\text{epi}}}{p^{\text{epi}}_{ij} - p^{\text{epi}}_{\text{max}} + \epsilon^{\text{epi}}}, && \text{(S31)} \\ & (i,j) = (1,1), \ldots, (N,M). \end{aligned}$$

If we assume that $p^{\text{mes}}_{\text{max}}$ or $p^{\text{epi}}_{\text{max}}$ is the turgor pressure when the water potential around the cell is zero, we can represent the above equation as,

$$\begin{aligned} C^{\text{mes}}_{ij} &= \frac{p^{\text{mes}}_{\text{max}}}{RT} \frac{\epsilon^{\text{mes}}}{p^{\text{mes}}_{ij} - p^{\text{mes}}_{\text{max}} + \epsilon^{\text{mes}}}, && \text{(S32)} \\ C^{\text{epi}}_{ij} &= \frac{p^{\text{epi}}_{\text{max}}}{RT} \frac{\epsilon^{\text{epi}}}{p^{\text{epi}}_{ij} - p^{\text{epi}}_{\text{max}} + \epsilon^{\text{epi}}}, && \text{(S33)} \\ & (i,j) = (1,1), \ldots, (N,M). \end{aligned}$$

Therefore, the parameters we should define for the calculation are only $p^{\text{mes}}_{\text{max}}$, $p^{\text{epi}}_{\text{max}}$, ϵ^{mes}, and ϵ^{epi}.

1.7 Stomatal conductance: Buckley et al. (2003)

For the model of stomatal conductance, one of the three models is used. We shall employ either the stomatal model by Buckley et al. (2003), or that due to Leuning (1995). The model by Buckley et al. (2003) is one of the most complicated models, in which mechanical processes including the stomatal opening via an increase in the turgor pressure of the guard cells is considered. In their model, it is assumed that stomatal conductance (g^{s}) is proportional to stomatal aperture, and the stomatal aperture is determined by the turgor pressures of the guard cells (p^{gu}) and epidermal cells (p^{epi}):

$$g^{\text{s}} = \chi(p^{\text{gu}} - \hat{m} p^{\text{epi}}), \tag{S34}$$

where χ is proportionality constants and $\hat{m}$ is the mechanical advantage of the epidermis (Buckley et al., 2003) which is usually more than 1 expressing the advantage of the effect of the turgor pressure of the epidermis relative to that of guard cell (Franks et al., 1998). In this study, for simplicity, we assumed the total pressure (the turgor pressure and the osmotic pressure) of a guard cell is the same as adjacent epidermal cells:

$$(p^{\text{gu}} - p^{\text{epi}}) = \sigma^{\text{gu-epi}} RT \Delta C^{\text{gu-epi}}. \tag{S35}$$

As a consequence, Eq (S34) can be rewritten as.

$$g_{ij}^{\text{s}} = \chi(p_{ij}^{\text{epi}} + \sigma_{ij}^{\text{gu-epi}} RTC_{ij}^{\text{gu}} - \sigma_{ij}^{\text{gu-epi}} RTC_{ij}^{\text{epi}} - \hat{m}p_{ij}^{\text{epi}}), \quad (i,j) = (1,1),\ldots,(N,M), \tag{S36}$$

where, $\sigma_{ij}^{\text{gu-epi}}$ is the reflection coefficient, and we set $\sigma_{ij}^{\text{gu-epi}} = 1$. In the model of Buckley et al. (2003), it is also assumed that the osmotic pressure in guard cells is mainly controlled by the concentration of Adenosine TriphosPhate (ATP) in guard cells, represented by the symbol τ. This assumption is based on the notion that ATP in guard cells is needed to drive proton pumping of potassium and the pumping rate is limited by ATP supply (Tominaga et al., 2001). The authors also proposed that the potential osmotic pressure in guard cells is affected by the turgor pressure in the adjacent epidermal cells. These two factors are linearly linked in their model, as shown here:

$$\Pi^{\text{apo-gu}} = -\sigma^{\text{apo-gu}} RT\Delta C^{\text{apo-gu}} = \beta\tau p^{\text{epi}}, \tag{S37}$$

where $\Pi^{\text{apo-gu}}$ is the osmotic pressure between the guard cell and the apoplastic region near the stomatal complex and β is the proportional constant (Buckley et al., 2003). If we assume that the solute concentration in the apoplastic region is nearly zero and $\sigma^{\text{apo-gu}} = 1$, we can represent Eq (S37) as,

$$g_{ij}^{\text{s}} = \chi(p_{ij}^{\text{epi}} + \beta\tau_{ij} p_{ij}^{\text{epi}} - \sigma_{ij}^{\text{gu-epi}} RTC_{ij}^{\text{epi}} - \hat{m}p_{ij}^{\text{epi}}). \quad (i,j) = (1,1),\ldots,(N,M). \tag{S38}$$

The supply rate of ATP is related to the rate of photosynthesis, and Buckley et al. (2003) associate the ATP concentration in the guard cells with the rate of RuBP carboxylation W_{c}. In turn, the rate of carboxylation that can be sustained by the current rate of electron transport W_{j}, based on the model by Farquhar and Wong (1984), is given by,

$$\tau_{ij-\text{c}} = \rho - c_{\text{p}} \frac{W_{ij-\text{c}}}{W_{ij-\text{j}}}, \tag{S39}$$

$$\tau_{ij-\text{j}} = (\rho - c_{\text{p}}) \frac{\dfrac{V_{\text{r}}}{V_{\text{m}}} - 1}{\dfrac{W_{ij-\text{c}} V_{\text{r}}}{W_{ij-\text{j}} V_{\text{m}}} - 1}, \tag{S40}$$

$$\tau_{ij} = \tau_0 + \begin{cases} \tau_{ij-\text{c}} (\text{if} \quad W_{ij-\text{c}} < W_{ij-\text{j}}) \\ \tau_{ij-\text{j}} (\text{else}) \end{cases}, \quad (i,j) = (1,1),\ldots,(N,M), \tag{S41}$$

where ρ is the total concentration of adenylates (τ + [ADP]), c_{p} is concentration of photophosphorylation sites, τ_0 is basal ATP level provided by other processes, and V_{r} and V_{m} is the maximum carboxylation rate limited by potential RuBP pool size and limited by Rubisco, respectively (Buckley et al., 2003). As

described above, the mechanical processes controlling stomatal aperture are explicitly included in the model.

1.8 Stomatal conductance: Franks et al. (1998) (non-linear Buckley et al. model)

In the nonlinear version of Buckley et al.'s stomatal conductance model, Equation (S34) is replaced by

$$g^{\mathrm{s}}_{ij} = \frac{\chi}{c_{\mathrm{F}}} a = \chi \left[f_1\left(p^{\mathrm{gu}}_{ij}\right) - \frac{p^{\mathrm{layer:4}}_{ij}}{p^{\mathrm{ep}}_{\mathrm{F,max}}} \left(f_1\left(p^{\mathrm{gu}}_{ij}\right) - f_2\left(p^{\mathrm{gu}}_{ij}\right)\right)\right], \tag{S42}$$

where f_1 and f_2 are two nonlinear functions of guard cell turgor pressure. c_{F} and $p^{\mathrm{ep}}_{\mathrm{F,max}}$ are parameters. These have been established, experimentally, by Franks et al. (1998) to have forms resembling the following functions:

$$f_1(p^{\mathrm{gu}}) = f_1^{\infty}\left(1 - e^{-p^{\mathrm{gu}}/p^{\mathrm{gu}}_{1,0}}\right) \quad ; \quad f_2(p^{\mathrm{gu}}) = \frac{f_2^{\infty}}{2}\left(\tanh(p^{\mathrm{gu}} - p^{\mathrm{gu}}_{2,0}) + 1\right), \tag{S43}$$

where f_1^{∞} and f_2^{∞} are some constants representing limiting values of f_1 and f_2 at high turgor pressures, and the two $p^{\mathrm{gu}}_{1,0}$ and $p^{\mathrm{gu}}_{2,0}$, parameters are constant turgor pressure scalings. In the present paper we have chosen to follow the lead of Franks et al. (1998) and use the so-called sigmoidal functional form

$$f_{1(2)}(p^{\mathrm{gu}}) = \frac{f^{\infty}_{1(2)}}{1 + e^{-\phi^{\mathrm{gu}}_{1(2),1}\cdot p^{\mathrm{gu}} + \phi^{\mathrm{gu}}_{1(2),2}}}, \tag{S44}$$

for both f_1 and f_2, but with different, fitting parameters f_1^{∞}, f_2^{∞}, $\phi^{\mathrm{gu}}_{1,1}$, $\phi^{\mathrm{gu}}_{2,1}$, $\phi^{\mathrm{gu}}_{1,2}$ and $\phi^{\mathrm{gu}}_{2,2}$. These constants are determined by fitting such mathematical functions to the measured data sets (Figure 4 in Franks et al. (1998))

The subsequent details of the Buckley et al. (2003) model remain the same in this nonlinear version as in the original version.

1.9 Stomatal conductance: Leuning (1995)

In the model that was proposed by Leuning (1995), the relationship between the stomatal conductance, the photosynthesis rate, the index of humidity, and CO_2 concentration is described as below:

$$\begin{aligned} g^{\mathrm{s}}_{ij-\mathrm{c}} &= g^{\mathrm{s}}_0 + b_{\mathrm{l}} \frac{P_{ij}}{(\gamma^{\mathrm{s}} - \Gamma'_{ij})(1 + D^{\mathrm{Pa}}_{ij}/D_0)}, \\ & \qquad (i,j) = (1,1), \ldots, (N,M), \end{aligned} \tag{S45}$$

where D_0 and b_{l} are empirical coefficients, γ^{s} is the CO_2 concentration at the leaf surface, Γ'_{ij} is the photorespiratory compensation point, D^{Pa}_{ij} is the vapor pressure deficit with unit of Pa, P_{ij} is the photosynthesis rate, and g^{s}_0 is the stomatal conductance when P_{ij} is zero. In this model, the variables are nonlinearly dependent on each other. For instance, stomatal conductance is affected by the photosynthesis rate, while photosynthesis itself is affected by the stomatal conductance.

Since we numerically calculate the turgor pressure of an epidermal cell, we can calculate the solute concentration in epidermal cells from Equation S33. The osmotic pressures and hydraulic pressures in epidermal cells can then be determined. As we assume hydraulic pressure in a guard cell equals that in an epidermal cell, we can also calculate the turgor pressure of guard cells even with the model of Leuning

(1995), through application of Equation S37. Therefore, although there is no interaction between the stomatal aperture and the turgor pressure of the guard cell in Equation S45, we still detain distribution patterns of guard cell turgor pressure even in the Leuning model (see Figure 6 in the main text). In other words, although turgor pressure of guard cells is not directly connected to stomatal conductance in the model of Eq. S45, the turgor pressure is indirectly affected by stomatal conductance calculated with Equation S45 because the stomatal conductance affects the transpiration rate and the transpiration rate determines the hydraulic pressure of the leaf. To be precise, in our whole leaf Leuning model, only the equation for calculating stomatal conductance derives from the Leuning stomatal conductance model. Therefore, the results of the guard cell turgor pressure, our Leuning implementation are actually a mixture of Leuning and Buckley et al. models, referred to simply as Leuning model results in the paper.

1.10 Intercellular CO_2 concentration

We assume intercellular CO_2 concentration and we apply the conservation constant of zero net CO_2 flow out of the target node:

$$\sum_{d=1}^{8} J_{ij-d} + J_{ij-\mathrm{S}} + J_{ij-\mathrm{A}} + J_{ij-\mathrm{H}} = 0, \quad (i,j) = (1,1), \ldots, (N,M), \tag{S46}$$

where J_{ij-d} is the flux in one of the eight directions, $J_{ij-\mathrm{S}}$ is the CO_2 absorption from the stomatal aperture, $J_{ij-\mathrm{A}}$ is CO_2 consumption by photosynthesis, and $J_{ij-\mathrm{H}}$ is the CO_2 production by the respiration. In this equation, we do not define the exact location (layer) at which the CO_2 dynamics simulated. Instead, we assume that the dynamics described above represent the average flows within and among node(s) that should occur mainly in mesophyll and epidermis. For the flow among nodes, we assume the diffusion of CO_2 via plasmodesmata. Therefore,

$$J_{ij-d} = B_{ij-d}(\gamma^{\mathrm{i}}_{ij} - \gamma^{\mathrm{i}}_{d}), \quad (i,j) = (1,1), \ldots, (N,M), \tag{S47}$$

where B_{ij-d} is the conductance between nodes in which the diffusivity of CO_2 and the distance between nodes are considered. γ^{i}_{ij} is the intercellular CO_2 concentration at node ij and γ^{i}_{d} is the intercellular CO_2 concentration at the node of the relevant direction. $J_{ij-\mathrm{S}}$ is described as below.

$$J_{ij-\mathrm{S}} = -a_{ij} g^{\mathrm{all}}_{ij-\mathrm{c}}(\gamma^{\mathrm{i}}_{ij} - \gamma^{\mathrm{s}}), \quad (i,j) = (1,1), \ldots, (N,M), \tag{S48}$$

where $g^{\mathrm{all}}_{ij-\mathrm{c}}$ is the CO_2 conductance between intercellular space and leaf surface. $J_{ij-\mathrm{A}}$ and $J_{ij-\mathrm{H}}$ are described as below.

$$J_{ij-\mathrm{A}} + J_{ij-\mathrm{H}} = -a_{ij}P_{ij-0} + a_{ij}H_{ij} = -a_{ij}P_{ij}, \quad (i,j) = (1,1), \ldots, (N,M), \tag{S49}$$

where P_{ij-0} is the photosynthesis rate in which the respiration is not considered, H_{ij} is the respiration rate, and P_{ij} is the net photosynthesis rate.

1.11 Photosynthesis

As the mathematical description of photosynthesis, the model by Farquhar et al. (1980) is applied. In that model, the net photosynthesis rate P_{ij} is determined by the Rubisco-limited carboxylation rate, W_{c}, and the RuBP-(Ribulose-1,5-bisphosphate)-limited carboxylation rate, W_j:

$$P_{ij} = (1 - \frac{\Gamma_{ij}}{\theta^{\mathrm{i}}_{ij-\mathrm{c}}}) \cdot \min\{W_{ij-\mathrm{c}}, W_{ij-\mathrm{j}}\} - H_{ij}, \quad (i,j) = (1,1), \ldots, (N,M). \tag{S50}$$

where Γ_{ij} is the photorespiratory compensation point, $\theta^{\mathrm{i}}_{ij-\mathrm{c}}$ is the intercellular CO_2 partial pressure, and H_{ij} is the respiration rate (Farquhar et al., 1980; Buckley et al., 2003).

In the model of Farquhar et al. (1980), the Rubisco-limited rate is represented the Michaelis-Menten equation reaching the plateau at V_{m} and the RuBP-limited rate is represented by the function of the light-limited potential electron transport rate (J):

$$W_{ij-\mathrm{c}} = \frac{V_{\mathrm{m}}\theta^{\mathrm{i}}_{ij-\mathrm{c}}}{\theta^{\mathrm{i}}_{ij-\mathrm{c}} + \omega_{\mathrm{c}}(1 + \theta^{\mathrm{i}}_{ij-\mathrm{o}}/\omega_{\mathrm{o}})}, \tag{S51}$$

$$W_{ij-\mathrm{j}} = \frac{Q_{ij}\theta^{\mathrm{i}}_{ij-\mathrm{c}}}{4(\theta^{\mathrm{i}}_{ij-\mathrm{c}} + 2\Gamma_{ij})}, \quad (i,j) = (1,1), \ldots, (N,M), \tag{S52}$$

where ω_{c} and ω_{o} are the Michaelis constant for carboxylation and oxygenation, respectively, and $\theta^{\mathrm{i}}_{ij-\mathrm{o}}$ is O_2 partial pressure (Farquhar et al., 1980; Buckley et al., 2003). The value of Q is represented by the function of the incident irradiance (I), in which the value reaches plateau at Q_{m}:

$$Q = \frac{\lambda' I + Q_{\mathrm{m}} - \sqrt{(\lambda' I + Q_{\mathrm{m}})^2 - 4\lambda' Q_{\mathrm{m}}\Theta'}}{2\Theta'}, \tag{S53}$$

where λ' is the product of leaf absorptivity to PAR and the effective quantum yield Buckley et al. (2003) and Θ' is the parameter that determines the curvature of the function.

1.12 Boundary conditions

We set the length of the petiole as 0.03 m with the xylem conductance of $\kappa^{\mathrm{xyl}}_{\mathrm{peti}}$ and the phloem conductance of $\kappa^{\mathrm{ph}}_{\mathrm{peti}}$. Between the petiole and the root, we assumed a simple "stem" that has the xylem conductance of $\kappa^{\mathrm{xyl}}_{\mathrm{stem}}$ and the phloem conductance of $\kappa^{\mathrm{ph}}_{\mathrm{stem}}$ with a length of 3.00 m. At the root, we arbitrarily set to - 0.50 MPa for the hydraulic pressure in the xylem, while the pressure in the phloem was set to 0.10 MPa. With regard to sucrose transport, we adopt a zero Neumann boundary condition at the root, which is equivalent to stating that there is no flow of sucrose from the root to the outside of the root. The choices of these types of boundary conditions, as well as the actual values set, are purely for pragmatic reasons as we wish to explore the effects of other variables on water and solute transport.

1.13 Parameter calibration

For unite the reference value of the output of the models, the parameter g^{s}_0, D_0, c_{F} for Leuning (1995) model and Franks et al. (1998) model were calibrated to fit the transpiration rate of the Buckley et al. (2003)

model under middle condition. The calculated average transpiration rate of the leaf of the Buckley et al. model was -1.30 $\mathrm{mmol\,m^{-2}\,s^{-1}}$, and calibrated values for the Leuning model and Franks et al. model were -1.29 and -1.29 $\mathrm{mmol\,m^{-2}\,s^{-1}}$, respectively.

REFERENCES

Batchelor, G. K. (1967). *An introduction to fluid dynamics* (Cambridge University Press)

Buckley, T. N., Mott, K. A., and Farquhar, G. D. (2003). A hydromechanical and biochemical model of stomatal conductance. *Plant, Cell & Environment* 26, 1767–1785

Cochard, H., Nardini, A., and Coll, L. (2004). Hydraulic architecture of leaf blades: where is the main resistance? *Plant, Cell & Environment* 27, 1257–1267

Daudet, F.-A., Lacointe, A., Gaudillere, J., and Cruiziat, P. (2002). Generalized münch coupling between sugar and water fluxes for modelling carbon allocation as affected by water status. *Journal of Theoretical Biology* 214, 481–498

Farquhar, G. D., von Caemmerer, S. v., and Berry, J. A. (1980). A biochemical model of photosynthetic co 2 assimilation in leaves of c 3 species. *Planta* 149, 78–90

Farquhar, G. D. and Wong, S. C. (1984). An empirical model of stomatal conductance. *Australian Journal of Plant Physiology* 11, 191–210

Foster, K. J. and Miklavcic, S. J. (2014). On the competitive uptake and transport of ions through differentiated root tissues. *Journal of theoretical biology* 340, 1–10

Foster, K. J. and Miklavcic, S. J. (2016). Modeling root zone effects on preferred pathways for the passive transport of ions and water in plant roots. *Frontiers in plant science* 7, 1–14

Franks, P. J., Cowan, I. R., and Farquhar, G. D. (1998). A study of stomatal mechanics using the cell pressure probe. *Plant, Cell & Environment* 21, 94–100

Jones, G. H. (2014). *Plants and Microclimate: A Quantitative Approach to Environmental Plant Physiology* (Cambridge University Press)

Katchalsky, A. and Curran, P. F. (1965). *Nonequilibrium thermodynamics in biophysics* (Harvard University Press)

Kramer, P. J. and Boyer, J. S. (1995). *Water relations of plants and soils* (Academic press)

Leuning, R. (1995). A critical appraisal of a combined stomatal-photosynthesis model for c3 plants. *Plant, Cell & Environment* 18, 339–355

Sakurai, G. and Miklavcic, S. J. (2021). On the efficacy of water transport in leaves. a coupled xylem-phloem model of water and solute transport. *Frontiers in plant science* 12, 17

Sellers, P., Randall, D., Collatz, G., Berry, J., Field, C., Dazlich, D., et al. (1996). A revised land surface parameterization (sib2) for atmospheric gcms. part i: Model formulation. *Journal of climate* 9, 676–705

Tominaga, M., Kinoshita, T., and Shimazaki, K.-i. (2001). Guard-cell chloroplasts provide atp required for h+ pumping in the plasma membrane and stomatal opening. *Plant and Cell Physiology* 42, 795–802

2 SUMMARY TABLES OF VARIABLES AND PARAMETER VALUES

Table S1. Summary of variables 1 (at node ij, subscripts not shown)

Symbol	Description	Units
F_d^{ph}	Phloem water flux (lateral direction)	$\mathrm{mmol\,s^{-1}}$
F_d^{xyl}	Xylem water flux (lateral direction)	$\mathrm{mmol\,s^{-1}}$
F_d^{mes}	Mesophyll water flux (lateral direction)	$\mathrm{mmol\,s^{-1}}$
F_d^{epi}	Epidermal water flux (lateral direction)	$\mathrm{mmol\,s^{-1}}$
$F_{\mathrm{px}}^{\mathrm{ph}}$	Flux from phloem to xylem	$\mathrm{mmol\,s^{-1}}$
$F_{\mathrm{px}}^{\mathrm{xyl}}$	Flux from xylem to phloem	$\mathrm{mmol\,s^{-1}}$
$F_{\mathrm{xm}}^{\mathrm{xyl}}$	Flux from xylem to mesophyll	$\mathrm{mmol\,s^{-1}}$
$F_{\mathrm{xm}}^{\mathrm{mes}}$	Flux from mesophyll to xylem	$\mathrm{mmol\,s^{-1}}$
$F_{\mathrm{me}}^{\mathrm{mes}}$	Flux from mesophyll to epidermis	$\mathrm{mmol\,s^{-1}}$
$F_{\mathrm{me}}^{\mathrm{epi}}$	Flux from epidermis to mesophyll	$\mathrm{mmol\,s^{-1}}$
$F_{\mathrm{T}}^{\mathrm{mes}}$	Transpiration from mesophyll	$\mathrm{mmol\,s^{-1}}$
$F_{\mathrm{T}}^{\mathrm{epi}}$	Transpiration from epidermis	$\mathrm{mmol\,s^{-1}}$
p^{xyl}	Xylem hydraulic pressure	MPa
p^{ph}	Phloem turgor pressure	MPa
p^{mes}	Mesophyll turgor pressure	MPa
p^{epi}	Epidermal turgor pressure	MPa
p^{gu}	Turgor pressure of guard cell	MPa
C^{ph}	Phloem sucrose concentration	$\mathrm{mol\,m^{-3}}$
C^{mes}	Mesophyll solute concentration	$\mathrm{mol\,m^{-3}}$
C^{epi}	Epidermal solute concentration	$\mathrm{mol\,m^{-3}}$
S_d	Phloem sucrose flux (lateral direction)	$\mathrm{mol\,s^{-1}}$
S_{L}	Phloem sucrose loading	$\mathrm{mol\,s^{-1}}$
J_d	CO_2 flux within inter-cellular space in the leaf (lateral direction)	$\mathrm{\mu mol\,s^{-1}}$
J_S	CO_2 absorption from the stomatal aperture	$\mathrm{\mu mol\,s^{-1}}$
J_A	CO_2 consumption by photosynthesis	$\mathrm{\mu mol\,s^{-1}}$
J_H	CO_2 production by the respiration	$\mathrm{\mu mol\,s^{-1}}$

Table S2. Summary of variables 2 (at node ij, subscripts not shown)

Symbol	Description	Units
P	Photosynthesis rate	$\mu mol\, s^{-1}\, m^{-2}$
Λ	Local sucrose loading rate	$mol\, s^{-1}\, m^{-2}$
D^{s}	Difference in water vapor mole fraction between the intercellular spaces and the air	$mmol\, mol^{-1}$
τ	Concentration of ATP	$mmol\, m^{-2}$
W_{c}	Carboxylation rate limited by CO_2 and Rubisco, but not by RuBP	$\mu mol\, s^{-1}\, m^{-2}$
W_j	Carboxylation rate limited by RuBP and CO_2, but not by Rubisco	$\mu mol\, s^{-1}\, m^{-2}$
g_w^{all}	Conductance of water vapor between the intercellular spaces and the air	$mol\, m^{-2}\, s^{-1}$
g^{s}	Stomatal conductance	$mol\, m^{-2}\, s^{-1}$
g_{c}^{s}	Stomatal conductance (CO_2)	$mol\, m^{-2}\, s^{-1}$
g_{w}^{s}	Stomatal conductance (water vapor)	$mol\, m^{-2}\, s^{-1}$
D^{kPa}	Vapor pressure difference (kPa)	kPa
D^{Pa}	Vapor pressure difference (Pa)	Pa
γ^{i}	intercellular CO_2 concentration	$\mu mol\, mol^{-1}$
θ_c^{i}	intercellular CO_2 partial pressure	Pa

Table S3. Summary of parameters 3 (at node ij, subscripts not shown)

Symbol	Description	Units
η	Proportion of phloem loading rate to photosynthesis rate	$\mathrm{mol}\,\mu\mathrm{mol}^{-1}$
ζ	Proportion of cross-sectional area of phloem conduit to that of xylem conduit	-
K^{xyl}	Xylem hydraulic conductance	$\mathrm{mmol\,s^{-1}\,MPa^{-1}}$
K^{ph}	Phloem hydraulic conductance	$\mathrm{mmol\,s^{-1}\,MPa^{-1}}$
K^{mes}	Mesophyll hydraulic conductance	$\mathrm{mmol\,s^{-1}\,MPa^{-1}}$
K^{epi}	Epidermal hydraulic conductance	$\mathrm{mmol\,s^{-1}\,MPa^{-1}}$
K_{px}	Phloem/xylem connection hydraulic conductance	$\mathrm{mmol\,s^{-1}\,MPa^{-1}}$
K_{xm}	Xylem/mesophyll connection hydraulic conductance	$\mathrm{mmol\,s^{-1}\,MPa^{-1}}$
K_{me}	Mesophyll/epidermis connection hydraulic conductance	$\mathrm{mmol\,s^{-1}\,MPa^{-1}}$
κ^{xyl}	Xylem hydraulic conductance (unit length)	$\mathrm{mmol\,s^{-1}\,MPa^{-1}\,m}$
κ^{ph}	Phloem hydraulic conductance (unit length)	$\mathrm{mmol\,s^{-1}\,MPa^{-1}\,m}$
κ^{mes}	Mesophyll hydraulic conductance (unit length)	$\mathrm{mmol\,s^{-1}\,MPa^{-1}\,m}$
κ^{epi}	Epidermal hydraulic conductance (unit length)	$\mathrm{mmol\,s^{-1}\,MPa^{-1}\,m}$
σ	reflection coefficient	-
e^{mes}	Proportion of transpiration from mesophyll	-
e^{epi}	Proportion of transpiration from epidermis	-
r	Radius of xylem vein cross-section	$\mathrm{m^2}$
A^{ph}	Phloem vein cross-sectional area	$\mathrm{m^2}$
A^{xyl}	Xylem vein cross-sectional area	$\mathrm{m^2}$
a	2D area per grid point	$\mathrm{m^2}$
l	Distance between consecutive nodes	m
T	Temperature of the leaf	K
$p^{\text{mes}}_{\text{max}}$	Maximum turgor pressure at the maximum volume of water in the mesophyll cell	MPa
$p^{\text{epi}}_{\text{max}}$	Maximum turgor pressure at the maximum volume of water in the epidermal cell	MPa
ϵ^{mes}	Elasticity of the mesophyll cell	MPa
ϵ^{epi}	Elasticity of the epidermal cell	MPa

Table S4. Summary of parameters 4 (at node ij, subscripts not shown)

Symbol	Description	Units
χ	Turgor-to-conductance scaling factor	$\mathrm{mol\,m^{-2}\,s^{-1}\,MPa^{-1}}$
$\hat{m}$	Mechanical advantage of the epidermis	-
β	Hydromechanical/biochemical response parameter	$\mathrm{mmol^{-1}\,m^{2}}$
ρ	Total concentration of adenylates (t + [ADP])	$\mathrm{mmol\,m^{-2}}$
c_{p}	Concentration of photophosphorylation sites	$\mathrm{mmol\,m^{-2}}$
τ_0	Basal ATP level provided by other processes	$\mathrm{mmol\,m^{-2}}$
V_{r}	Carboxylation rate limited by potential RuBP pool size only	$\mathrm{\mu mol\,s^{-1}\,m^{-2}}$
V_{m}	Carboxylation rate limited by Rubisco only	$\mathrm{\mu mol\,s^{-1}\,m^{-2}}$
g_0^{s}	Stomatal conductance when P is zero	$\mathrm{mol\,m^{-2}\,s^{-1}}$
b_1	Empirical parameter	-
D_0	Empirical parameter	Pa
Γ'	Photorespiratory compensation point (ppm)	ppm
Γ	Photorespiratory compensation point (Pa)	Pa
H	Respiration rate	$\mathrm{\mu mol\,s^{-1}\,m^{-2}}$
Q_{m}	Light-saturated potential electron transport	$\mathrm{\mu mol\,s^{-1}\,m^{-2}}$
λ	Curvature parameter	-
Θ	Curvature parameter	-
λ'	Product of absorbance and effective quantum yield	-
Θ'	Curvature parameter	-
ω_{c}	Michaelis constant for RuBP carboxylation	Pa
ω_{c}	Michaelis constant for RuBP oxygenation	Pa
$\theta^{\mathrm{i}}_{ij-\mathrm{o}}$	O_2 partial pressure	Pa
B_d	intercellular CO_2 conductance between nodes	$\mathrm{mol\,s^{-1}}$
f_1^{∞}	Curvature parameter	$\mathrm{\mu m}$
f_2^{∞}	Curvature parameter	$\mathrm{\mu m}$
$\phi^{\mathrm{gu}}_{1,1}$	Curvature parameter	$\mathrm{MPa^{-1}}$
$\phi^{\mathrm{gu}}_{2,1}$	Curvature parameter	$\mathrm{MPa^{-1}}$
$\phi^{\mathrm{gu}}_{1,2}$	Curvature parameter	$\mathrm{MPa^{-1}}$
$\phi^{\mathrm{gu}}_{2,2}$	Curvature parameter	$\mathrm{MPa^{-1}}$
$p^{\mathrm{ep}}_{\mathrm{F,max}}$	Curvature parameter	MPa
c_{F}	Turgor-to-aperture scaling factor	$\mathrm{\mu m\,MPa^{-1}}$

Table S5. Parameter values 1 (fixed)

Symbol	Value	Reference
T	298.15	Arbitrary
e^{mes}	0.80	Arbitrary
e^{epi}	0.20	Arbitrary
η	0.50×10^{-6}	Arbitrary
κ^{xyl}	1.00×10^{-2} (1st order vein)	Cochard et al. (2004)
	5.00×10^{-4} (2nd order vein)	
	6.00×10^{-5} (3rd order vein)	
	4.00×10^{-5} (4th order vein)	
	4.00×10^{-6} (5th order vein)	
κ^{ph}	$=\zeta\kappa^{\text{xyl}}$	Daudet et al. (2002)
κ^{mes}	1.00×10^{-6}	Arbitrary
κ^{epi}	1.00×10^{-6}	Arbitrary
K_{px}	0.50	Daudet et al. (2002)
K_{xm}	5.00	Arbitrary
K_{me}	5.00	Arbitrary
r	7.75×10^{-6} (1st order vein)	Cochard et al. (2004)
	5.65×10^{-6} (2nd order vein)	
	3.75×10^{-6} (3rd order vein)	
	3.60×10^{-6} (4th order vein)	
	2.17×10^{-6} (5th order vein)	
ζ	0.03	Daudet et al. (2002)
$p_{\text{max}}^{\text{mes}}$	2.00	Arbitrary
$p_{\text{max}}^{\text{epi}}$	2.00	Arbitrary
ϵ^{mes}	20.00	Arbitrary
ϵ^{epi}	20.00	Arbitrary

Table S6. Parameter values 2 (fixed)

Symbol	Value	Reference
χ	0.105	Buckley et al. (2003)
$\hat{m}$	1.98	Buckley et al. (2003)
β	1.17	Buckley et al. (2003)
ρ	$12.6V_{\mathrm{m}}\times10^{-3}$	Buckley et al. (2003)
c_{p}	$2.5\ V_{\mathrm{m}}\times10^{-3}$	Buckley et al. (2003)
V_{r}	$2.27V_{\mathrm{m}}$	Buckley et al. (2003)
c_{p}	88.60	Buckley et al. (2003)
g_0^{s}	2.30×10^{-2}	Calibrated
b_{l}	2.00	Arbitrary
D_0	1000.00	Calibrated
Γ	$0.105\ \frac{\omega_{\mathrm{c}}\theta^{\mathrm{i}}_{ij-\mathrm{o}}}{\omega_{\mathrm{o}}}$	Buckley et al. (2003)
H	$0.015V_{\mathrm{m}}$	Sellers et al. (1996)
V_{m}	88.60	Buckley et al. (2003)
Q_{m}	$2.02\ V_{\mathrm{m}}$	Buckley et al. (2003)
λ'	0.195	Buckley et al. (2003)
Θ'	0.95	Buckley et al. (2003)
ω_{c}	40.40	Buckley et al. (2003)
ω_{o}	2.48×10^{3}	Buckley et al. (2003)
$\theta^{\mathrm{i}}_{ij-\mathrm{o}}$	2.10×10^{3}	Buckley et al. (2003)
f_1^{∞}	18.64	Calibrated
f_2^{∞}	9.36	Calibrated
$\phi^{\mathrm{gu}}_{1,1}$	1.95	Calibrated
$\phi^{\mathrm{gu}}_{2,1}$	2.84	Calibrated
$\phi^{\mathrm{gu}}_{1,2}$	1.10	Calibrated
$\phi^{\mathrm{gu}}_{2,2}$	7.28	Calibrated
$p^{\mathrm{ep}}_{\mathrm{F,max}}$	0.92	Calibrated
c_{F}	6.50	Calibrated

Table S7. Environmental parameter changed

Symbol	Description	Values	Units
γ^{s}	CO_2 concentration at the leaf surface	100, 400, 800	ppm
I	Incident photosynthetically active irradiance	200, 500, 1000	$\mu\mathrm{mol\,s^{-1}\,m^{-2}}$
R_{h}	Relative humidity	10, 50, 90	$\%$

Table S8. Other parameters needed for the calculation

Description	Values	Units
Sucrose permeability	5.22×10^{-10}	$\mathrm{m^2\,s^{-1}}$
CO_2 diffusion coefficient in cell	1.92×10^{-9}	$\mathrm{m^2\,s^{-1}}$
Atmospheric pressure	101325.00	Pa
Water volume per mmol	18.00×10^{-9}	$\mathrm{m^3\,mmol^{-1}}$
