# Supplementary material for: A Whole Leaf Comparative Study of Stomatal Conductance Models
Source: Front Plant Sci. 2022 Apr 11;13:766975. doi: 10.3389/fpls.2022.766975 (PMC9036488; doi:10.3389/fpls.2022.766975)
Supplement: Supplementary file 2 [file Data_Sheet_2.PDF]

Supplementary Information II: A whole leaf comparative study of stomatal conductance models

1 COMPLEMENTARY RESULTS

In this section we complement the results presented in the main article with ancillary figures here showing results of additional calculations that support the arguments and conclusions given in the paper.

1.1 The effect of vein tapering

In Figures S1-S3 we show results of the effect of vein tapering compared with a non-tapered vein system. The detailed figures show the effect on photosynthesis and transpiration occurring over the leaf, while Figure S3 provides a summary of the effect covering several variables.

The effects of tapering are demonstrated by Figures S1 and S2 showing the steady state activity of photosynthesis and transpiration taking place across the leaf. In these calculations we have implemented the linear hydromechanical model of stomatal conductance proposed by Buckley et al. (2003). In Figure S1 the comparison shows the qualitative effect of tapering, a result that is also reflected in the responses displayed by transpiration, stomatal conductance and xylem hydraulic pressure. In Figure S3, we provide, in the form of box and whisker plots, a quantitative summary of the effects of tapering on the distributions of these four quantities. In the case of transpiration, we see that there is a greater magnitude of transpiration occurring over a larger region of the leaf close to the petiole (relative to the same leaf with uniform conductivity). This effect may be explained as being a consequence of the tendency of water to follow paths of least resistance. With the reduced conductivities (or increased resistances) of 1st and 2nd order veins far from the petiole, all else being equal, the water potential in an enlarged region near the petiole is decreased (*i.e.*, becomes more negative) which drives more water out of this region via transpiration. Qualitatively speaking, the effect of tapering on transpiration is the same irrespective of relative humidity. Naturally, quantitatively speaking, the transpiration is greatly dependent on the degree of humidity external to the leaf, here decreasing an order of magnitude from a mean of approximately 2 mmol $m^{-1}s^{-1}$ in dry air to approximately 0.2 mmol $m^{-1}s^{-1}$ under high humidity. (Under still higher humidity conditions we find that water intrudes into the leaf.)

The high conductivity of a low order vein, the main vein or the second order veins, of angiosperms arises as a consequence of the greater volume of water transported through the many veinlets making up the vascular bundles. A subset of this collection successively diverges from the bundle to form a higher order branch. From the point of branching, the overall conductance of the parent vein then effectively decreases as less volume of water is carried on for the same water potential difference. The divergences first occur in all low order veins beginning with the main vein, and progresses with the higher order veins. In this way the effective conductivity of a nominal vein undergoes a step-wise decrease, as does the vascular bundle's overall diameter. This is what we have referred to as tapering.

As we did not include the feature of tapering in our previous calculations (Sakurai and Miklavcic, 2021), we have paid due diligence here to a consideration of this effect in a typical (arbitrary) hydraulic scenario. Cochard et al. (2004) conducted a study of the different ways in which vein tapering could be implemented in a hydraulic model of a leaf's vascular system. Comparing a uniform gradient (*i.e.*, linearly increasing)

hydraulic resistance with a nonlinear gradient, and one exhibiting discrete increases in vein resistance (in line with the above description); they found little difference in the ratio of transpiration to leaf water potential across the leaf. Based on this finding, we have chosen to implement the simplest tapering model: that of a uniform gradient of decreasing hydraulic conductivity in our first and second order veins, so as to determine the effects of these in our extended model. The linear gradient model was constructed to have the same average conductance (over the length of a given vein) as that of the same vein of uniform conductivity. All higher order veins (3rd - 6th) were taken as possessing uniform (low) hydraulic conductivity.

The comparison of tapered veins to non-tapered veins might suggest consequences for the degree to which the greater area of the leaf can be effectively hydrated in leaves whose veins suffer more rapid decreases in hydraulic conductivity, or increases in resistance. It is also interesting to reflect on what this might mean for water movement in leaves that are undergoing progressive senescence, with the senescent front encroaching from the leaf periphery toward the leaf interior.

Local transpiration and photosynthesis are oppositely correlated with relative humidity through the hydromechanical stomatal conductance model of Buckley et al. (2003). Despite this, tapering has a qualitatively similar effect on photosynthesis activity as it does on transpiration (with tapering photosynthesis production also increases on average). Similar to transpiration, there is little or no qualitative dependence of photosynthesis activity on relative humidity. However, in terms of magnitude there is of course a quantitative dependence on external conditions.

In one respect, there is no change with the introduction of tapering, that is in terms of optimum angle of second order veins. Although we have a more complex representation of a leaf's hydraulic system, the conclusions of Sakurai and Miklavcic (2021) remain unchanged even though the actual magnitudes of transpiration differ due to the reduced vein conductivities imposed by tapering.

Overall, the reader may agree that the response to tapering is unidirectional, in the sense that all quantities are increased on average. It is also unsurprising that, being consistent with the qualitative findings in Figure S1, the spreads of the distributions have been reduced. A number of other cases of different external conditions have also been studied and similar comparative outcomes have been found (results not shown).

1.2 Additional results: dependence on relative humidity, light intensity, temperature and leaf shape

In Figures S4 and S5 we complement the results provided in the main paper with further data using the three stomatal conductance models of Leuning (1995), and the linear and nonlinear versions of the Buckley et al. (2003) model. The figures cover ancillary results on guard cell turgor pressure as a function of the external conditions of relative humidity and leaf irradiance, respectively. The values of relative humidity are 10%, 50% and 90%, while the illumination intensities considered were defined as low (200 μmol m^{-2} s^{-1}), medium (500 μmol m^{-2} s^{-1}), and high irradiance (1000 μmol m^{-2} s^{-1}). These results are in correspondence with the information on photosynthesis and transpiration shown in Figures 7-8 and 9-10, respectively, of the paper.

The simulations varying CO_2 gas concentration (adopting denotations and values of low (100 ppm), medium (400 ppm) and high (800 ppm) concentration), relative humidity and light intensity were then repeated at the lower temperature of 10° C. The additional results, complementing Figures 11 and 12 of the paper, are here shown in Figures S6-S12.

Finally, the simulations at 25° C were again repeated this time for different leaf shapes: long/narrow leaves with an aspect ratio of $l : w = 1 : 0.28$, and short/wide leaves with an aspect ratio of $l : w = 1 : 0.63$.

The results shown here in Figures S13-S28 complete the data set, a subset of which is shown in Figures 13 and 14 of the paper.

1.3 A technical point of the Buckley et al. stomatal conductance model

To address a more detailed technical point, we show in Figure S29 the effect of humidity on the difference in carboxylation rates, $W_c - W_j$ across the leaf. This difference is featured in the Buckley et al. (2003) stomatal conductance model, Eqs 41 and 50 of Supplementary Information I (also Eq. (6) in the main text). The figure shows how, at low humidity, discontinuities in the gradients of photosynthesis rate, transpiration rate and guard cell turgor pressure arise along the main vein (or along any line from petiole out to the leaf periphery) as the photosynthesis rate switches from being Rubisco-limited to being RuBP-limited, or vice versa depending on which of the carboxylation rates is greatest.

REFERENCES

Buckley, T. N., Mott, K. A., and Farquhar, G. D. (2003). A hydromechanical and biochemical model of stomatal conductance. *Plant, Cell & Environment* 26, 1767–1785

Cochard, H., Nardini, A., and Coll, L. (2004). Hydraulic architecture of leaf blades: where is the main resistance? *Plant, Cell & Environment* 27, 1257–1267

Leuning, R. (1995). A critical appraisal of a combined stomatal-photosynthesis model for c3 plants. *Plant, Cell & Environment* 18, 339–355

Sakurai, G. and Miklavcic, S. J. (2021). On the efficacy of water transport in leaves. a coupled xylem-phloem model of water and solute transport. *Frontiers in plant science* 12, 17

2 FIGURES

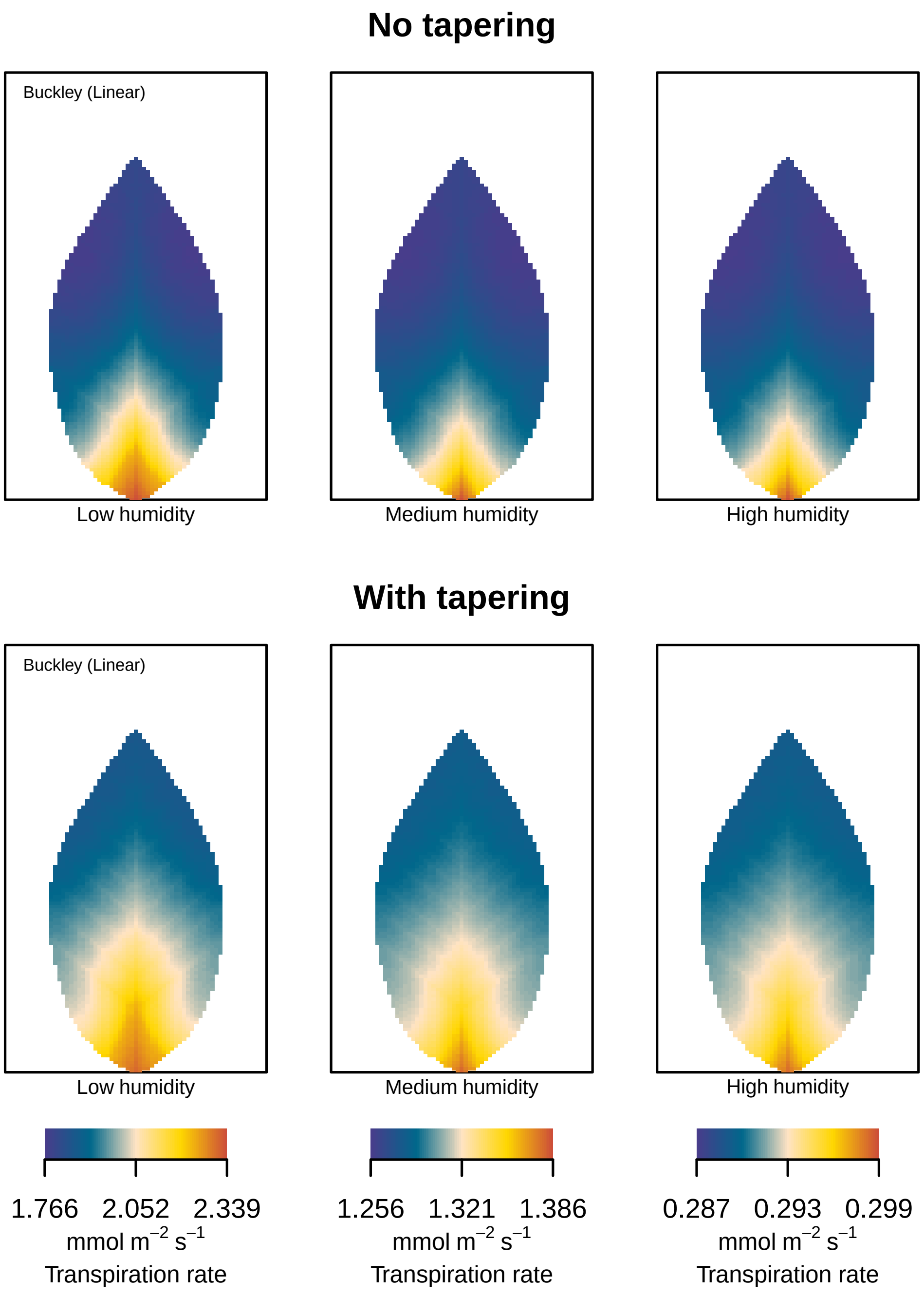

Figure S1. A comparison of the model's predictions of the transpiration rate ($-F_{\mathrm{T}}$), distributed over the leaf, under the assumption of tapering (linearly decreasing conductivity in 1st and 2nd order veins) and no tapering (uniform conductivity along every vein). The comparison also shows the dependence on external condition of relative humidity: low (10%), medium (50%) and high humidity (90%). Note that the scales appearing at the bottom of each column refer to the colors scheme in the panels of those respective columns, and *not* between columns.

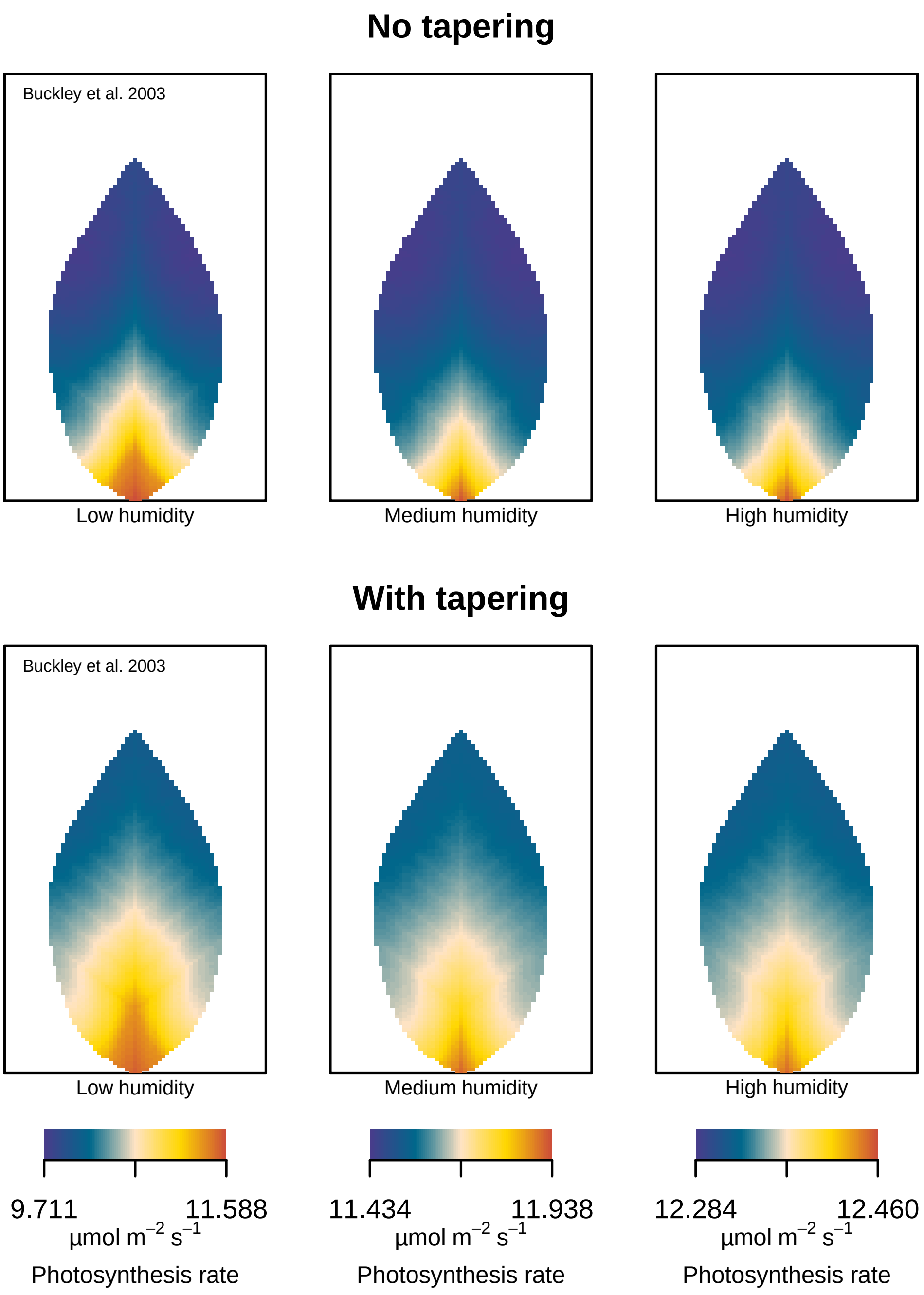

Figure S2. A comparison of the model's predictions of photosynthesis activity distributed over the leaf under the assumption of tapering (linearly decreasing conductivity in 1st and 2nd order veins) and no tapering (uniform conductivity along every vein). The comparison also shows the dependence on external condition of relative humidity: low (10%), medium (50%) and high humidity (90%). Note that the scales appearing at the bottom of each column refer to the color scheme in the panels of those respective columns, and not between columns.

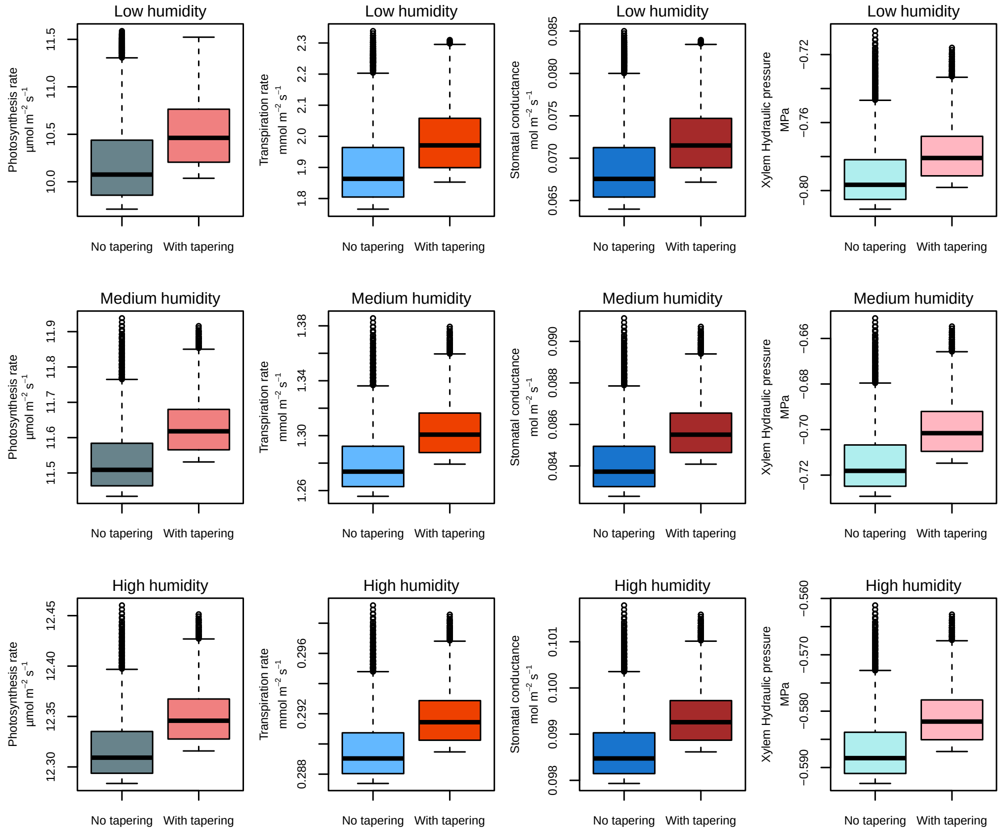

Figure S3. Summary "box and whisker" plots of the effect of tapering on a number of leaf properties. From left to right we show aggregate data of the variation of photosynthesis rate, transpiration rate, stomatal conductance and xylem hydraulic pressure over the leaf area. As usual the median value over the leaf is given as the thick black line in the centre of each box; the first (Q1) and third (Q3) quartiles of the distribution are indicated by the lower and upper ends of each box, respectively. The lowest data point value (which may be negative) is indicated by the lower-most thin horizontal line. The upper-most horizontal line indicates the standard 1.5 times the interquartile (Q1-Q3) range. The symbols represent extreme values beyond the latter indicator. In each of the 12 panels, the left box and whisker refers to the distribution found assuming no tapering, while the right box and whisker relates to the distribution found with vein tapering. The data assumes application of the Buckley et al. (2003) stomatal conductance model. The effect of tapering assumes a linearly decreasing conductivity in 1st and 2nd order veins, and no tapering (uniform conductivity) in other veins. The three rows show from top to bottom the response to changes in external relative humidity: low (10%), medium (50%) and high humidity (90%).

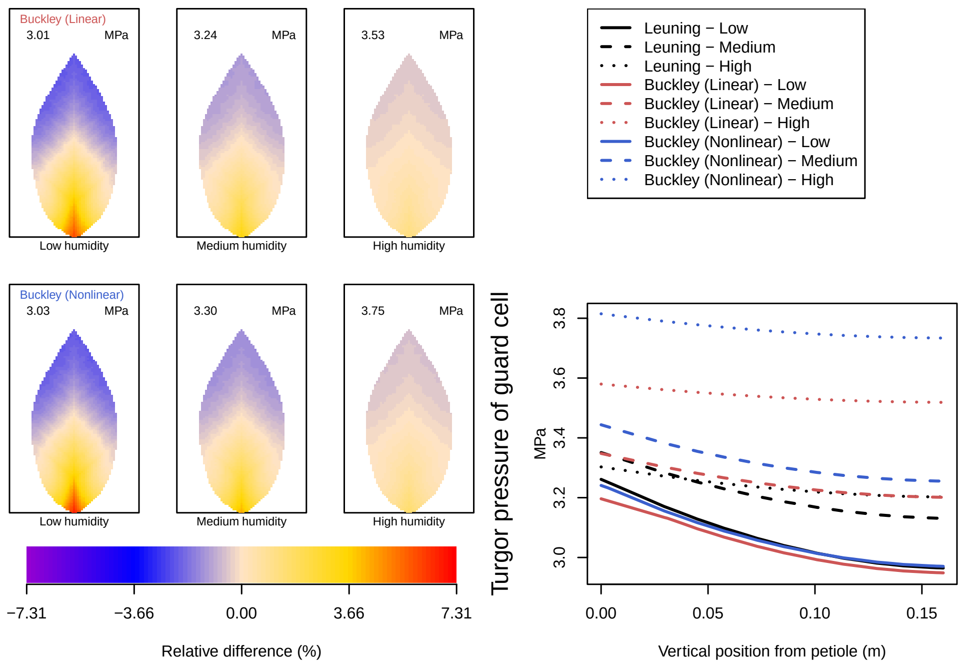

Figure S4. A comparison of predictions of guard cell turgor pressure distributed over the leaf according to the three stomatal conductance models of Leuning (1995) (black lines in right hand figure), linear Buckley et al. (2003) (top row of panels, and red lines in the right hand figure), and nonlinear Buckley et al. (2003) (bottom row of panels, and blue lines in right hand figure). The 2D maps shown are deviations from leaf-area averages. The leaf-area average values of turgor pressure are given at the top of each panel. The three columns (left-to-right) and line styles (solid, dashed, dotted) show the respective dependencies on **relative humidity**: low (10 %) ; medium (50 %); and high humidity (90 %). Other model parameter values can be found in Supplementary Information I.

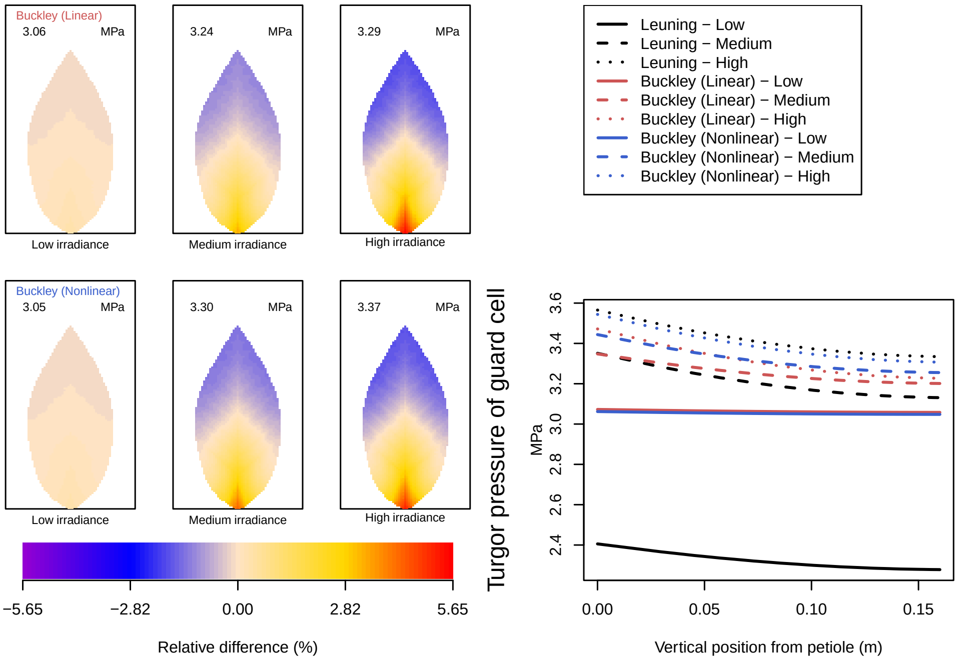

Figure S5. A comparison of predictions of guard cell turgor pressure distributed over the leaf according to the three stomatal conductance models of Leuning (1995) (black lines in right hand figure), linear Buckley et al. (2003) (top row of panels, and red lines in the right hand figure), and nonlinear Buckley et al. (2003) (bottom row of panels, and blue lines in right hand figure). The 2D maps shown are deviations from leaf-area averages. The leaf-area average values of turgor pressure are given at the top of each panel. The three columns (left-to-right) and line styles (solid, dashed, dotted) show the respective dependencies on **light intensity**: low (200 μmol m^{-2} s^{-1}); medium (500 μmol m^{-2} s^{-1}); and high irradiance (1000 μmol m^{-2} s^{-1}). Other model parameter values can be found in Supplementary Information I.

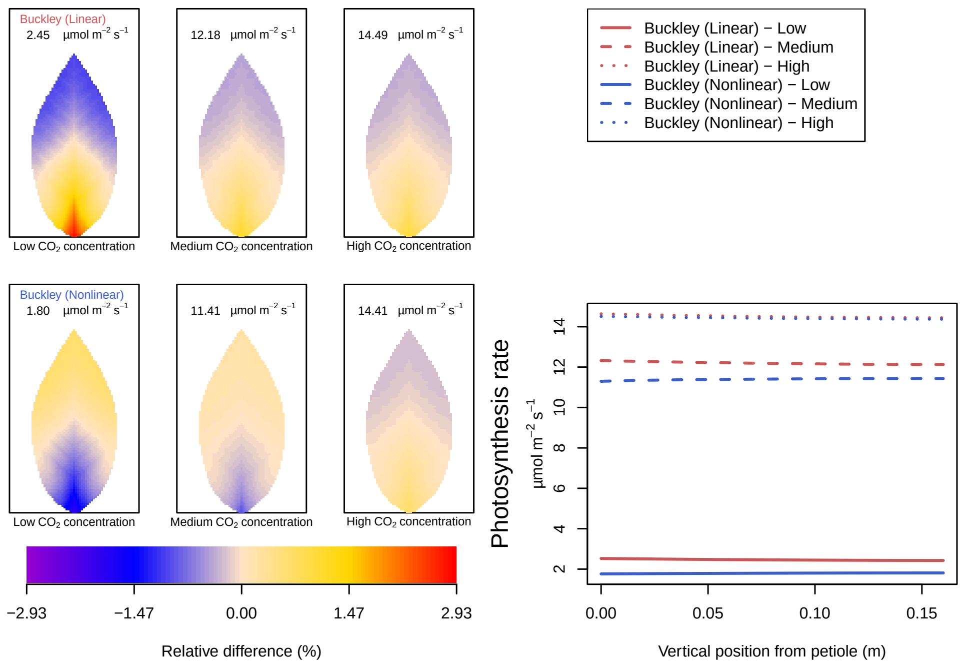

Figure S6. A comparison of model predictions of photosynthetic activity distributed over the leaf for different values of **external CO_2 concentration**. Details as in Figure 4 of the paper, except **for a temperature of 10° C (283.15 K)**. In this case the uniform/leaf-area average predictions using the Leuning (1995) stomatal model are 2.72, 13.29 and 17.37 μmol m^{-2} s^{-1} under low, medium and high CO_2 concentrations, respectively (the heat maps are not shown).

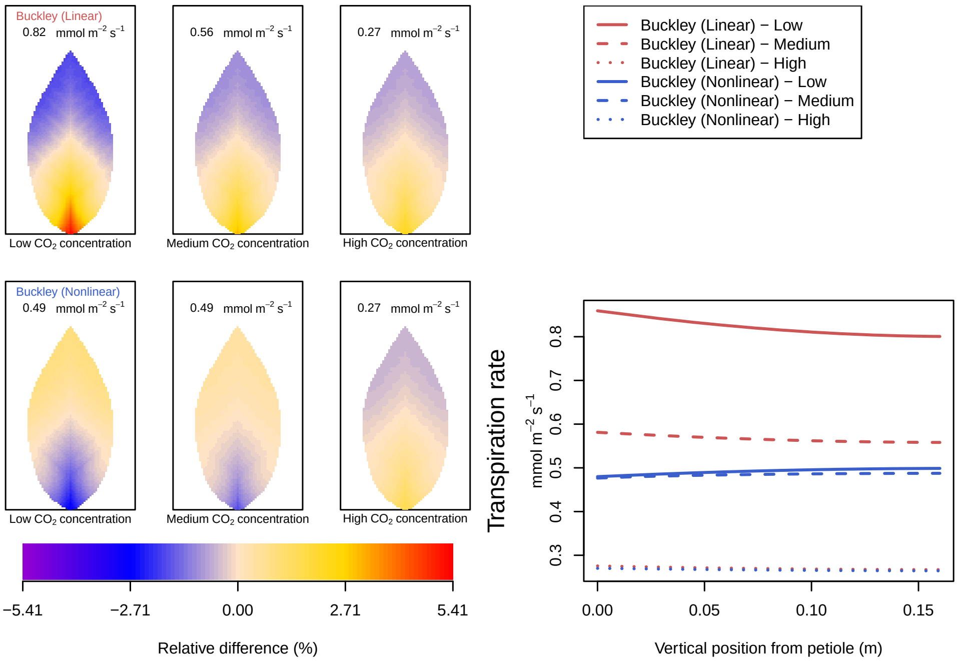

Figure S7. A comparison of model predictions of transpiration rates ($-F_{\mathrm{T}}$)distributed over the leaf for different values of **external CO_2 concentration**. Details as in Figure 5 of the paper, except **for a temperature of 10° C (283.15 K)**. In this case the uniform/leaf-area average predictions using the Leuning (1995) stomatal model are 0.75, 0.69 and 0.53 mmol m^{-2} s^{-1} under low, medium and high CO_2 concentrations, respectively (the heat maps are not shown).

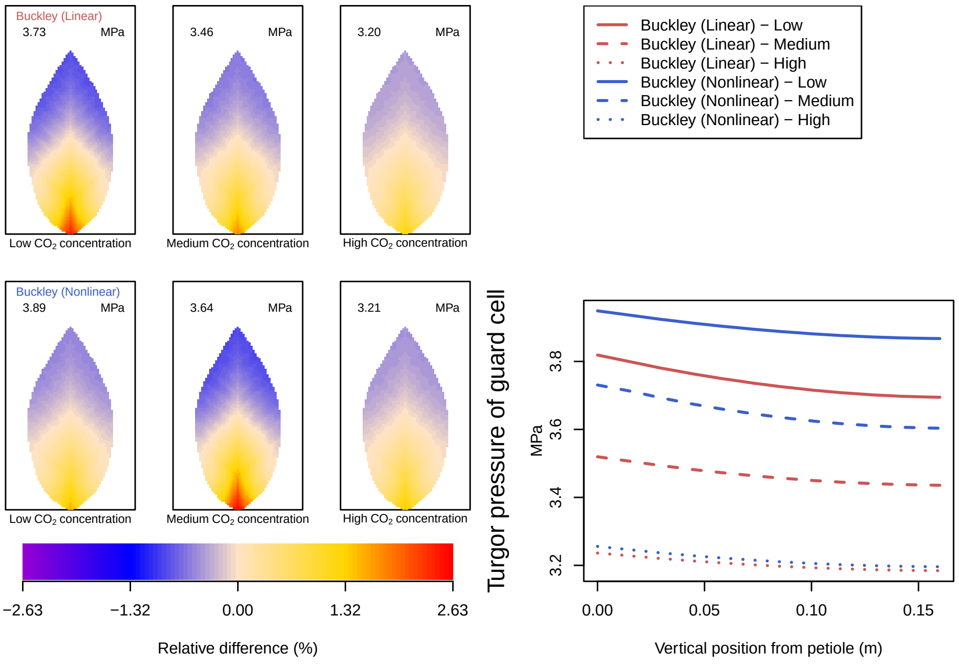

Figure S8. A comparison of model predictions of guard cell turgor pressure distributed over the leaf for different values of **external CO_2 concentration**. Details as in Figure 6 of the paper, except **for a temperature of 10° C (283.15 K)**.

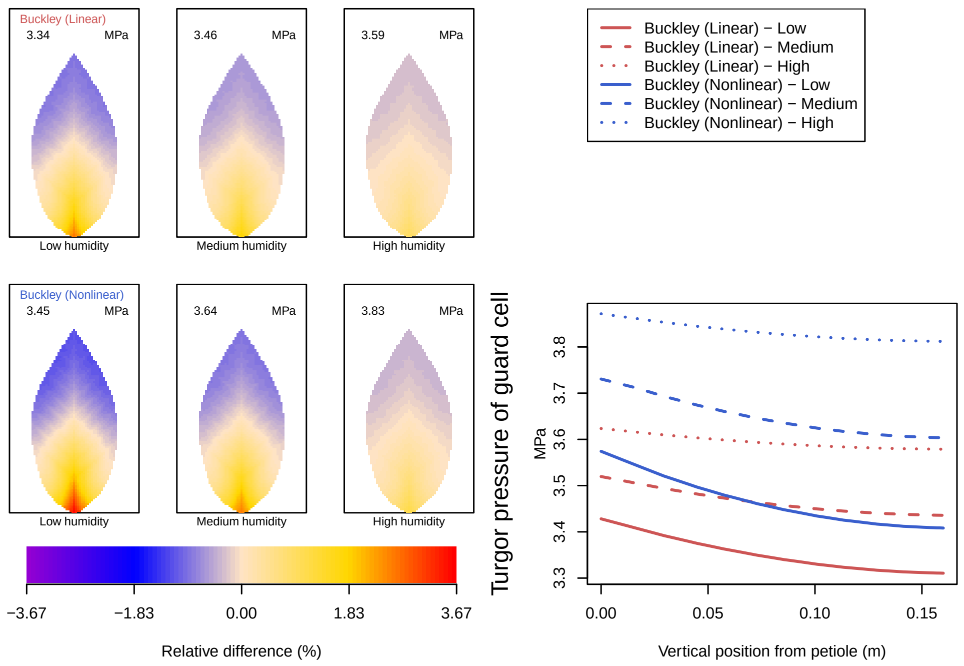

Figure S9. A comparison of model predictions of guard cell turgor pressure distributed over the leaf for different values of **relative humidity**. Details as in Figure S4, except **for a temperature of 10° C (283.15 K)**.

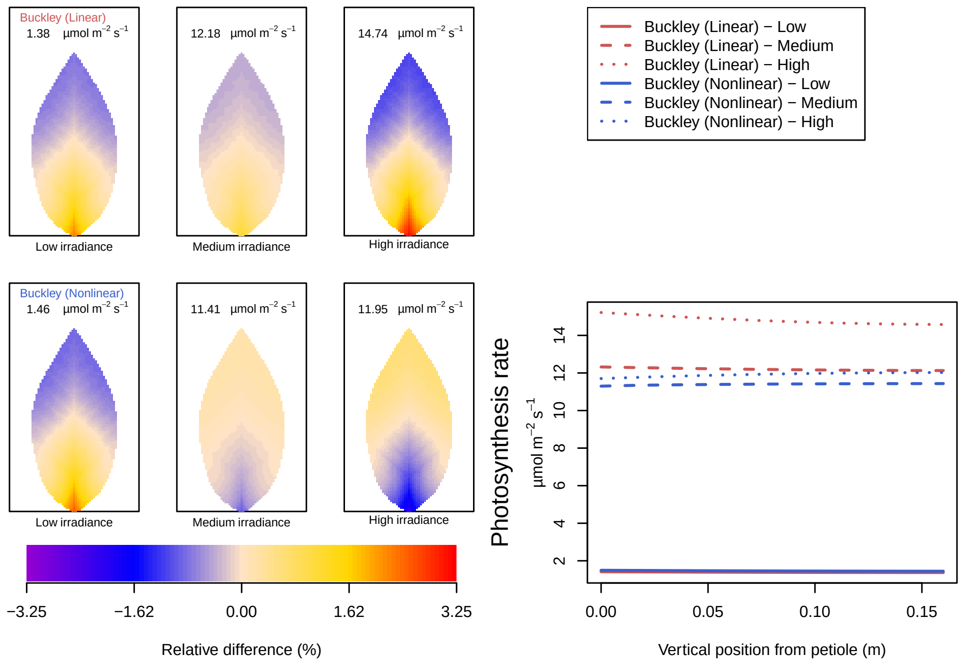

Figure S10. A comparison of model predictions of photosynthetic activity distributed over the leaf for different values of **light intensity**. Details as in Figure 9 of the paper, except **for a temperature of 10° C (283.15 K)**. In this case the uniform/leaf-area average predictions using the Leuning (1995) stomatal model are 5.43, 13.29 and 14.94 μmol m^{-2} s^{-1} under low, medium and high light intensity, respectively (the heat maps are not shown) (the heat maps are not shown).

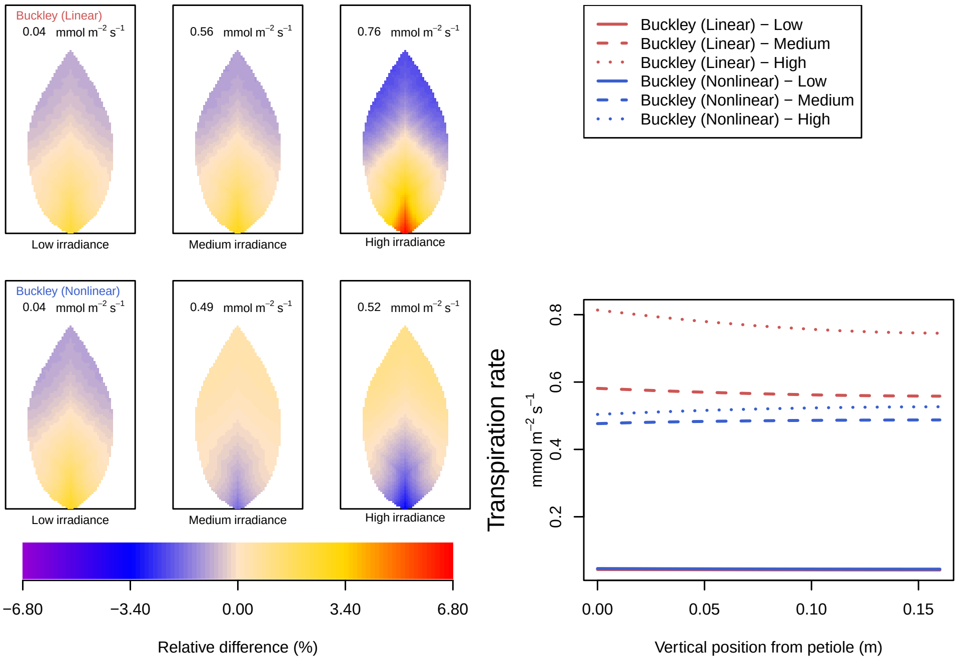

Figure S11. A comparison of model predictions of transpiration rates ($-F_{\mathrm{T}}$) distributed over the leaf for different values of **light intensity**. Details as in Figure 10 of the paper, except **for a temperature of 10° C (283.15 K)**. In this case the uniform/leaf-area average predictions using the Leuning (1995) stomatal model are 0.46, 0.69 and 0.74 mmol m^{-2} s^{-1} under low, medium and high light intensity, respectively (the heat maps are not shown).

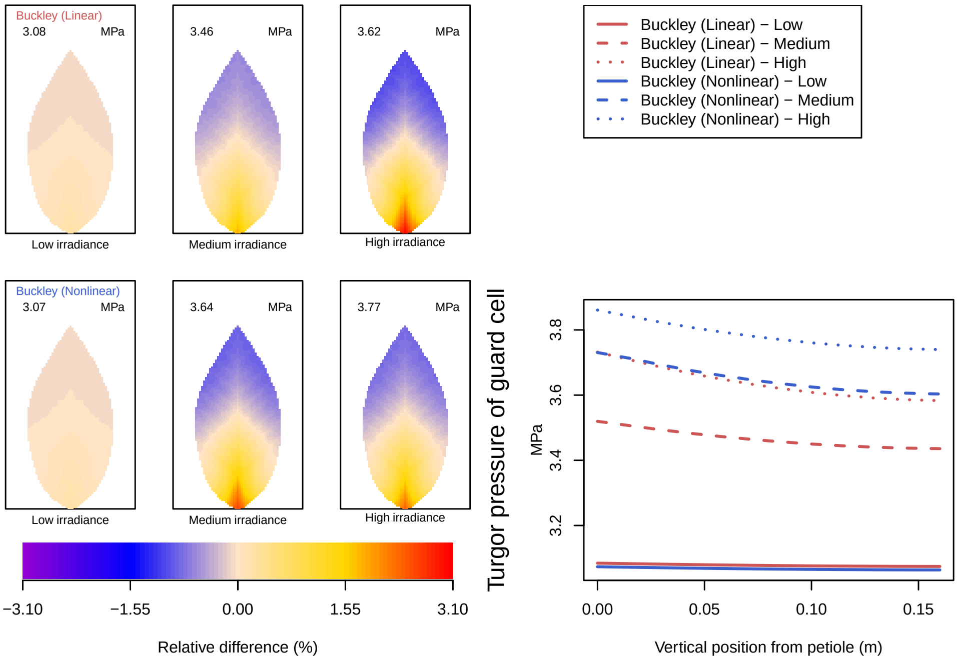

Figure S12. A comparison of model predictions of guard cell turgor pressure distributed over the leaf for different values of **light intensity**. Details as in Figure S5, except **for a temperature of 10° C (283.15 K)**.

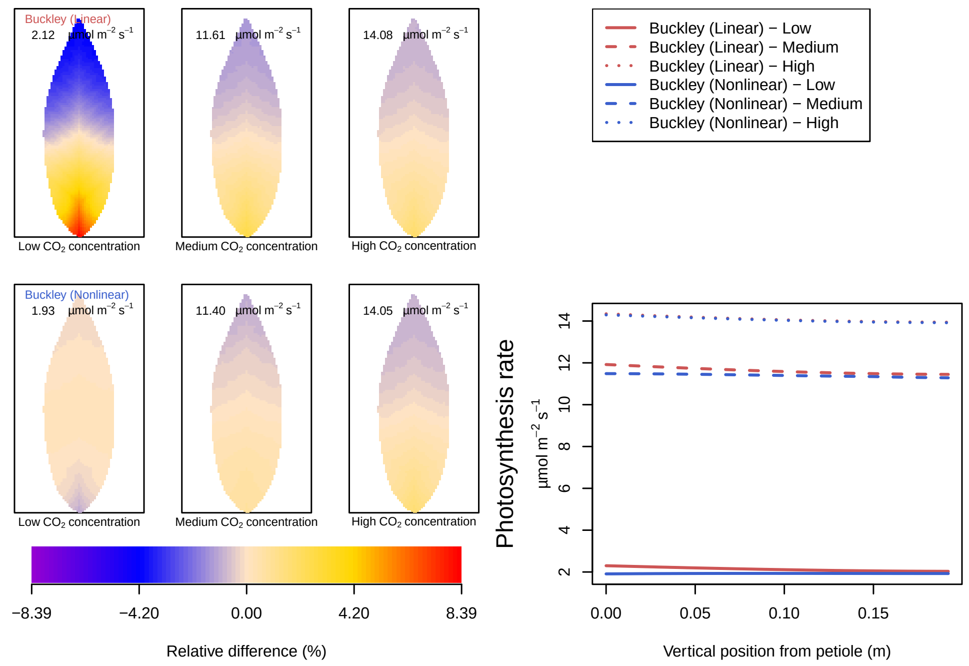

Figure S13. A comparison of model predictions of photosynthesis rate distributed over the leaf for different values of CO_2 concentration. Details as in Figure 4 of the paper, except for a longer/narrower leaf of aspect ratio $l : w = 1 : 0.28$. In this case the uniform/leaf-area average predictions using the Leuning (1995) stomatal model are 2.16, 11.81 and 16.73 μmol m^{-2} s^{-1} under low, medium and high CO_2 concentrations, respectively (the heat maps are not shown).

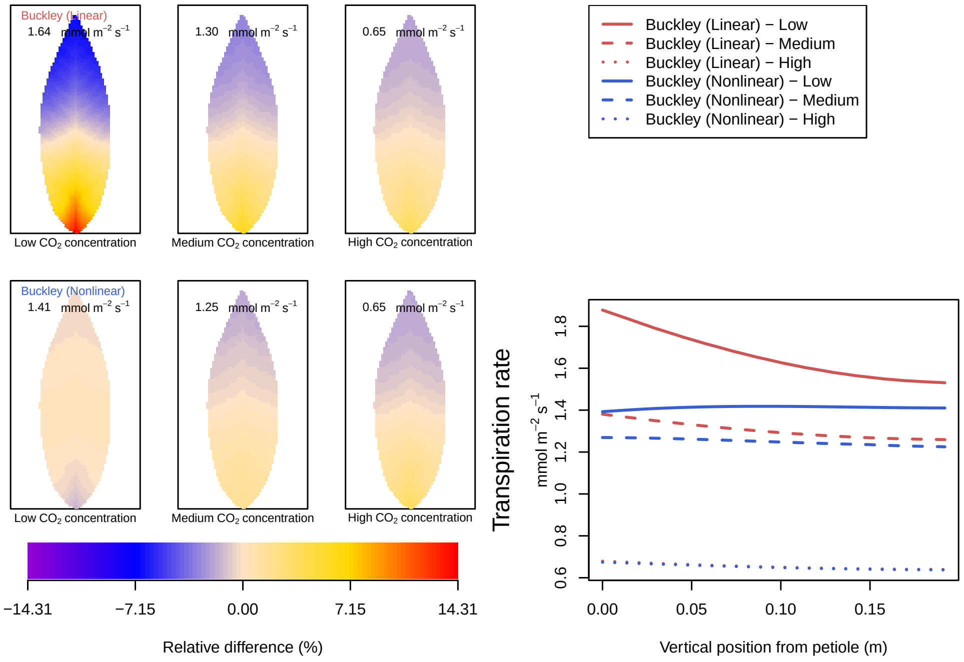

Figure S14. A comparison of model predictions of transpiration rate ($-F_{\mathrm{T}}$) distributed over the leaf for different values of CO_2 concentration. Details as in Figure 5 of the paper, except for a longer/narrower leaf of aspect ratio $l : w = 1 : 0.28$. In this case the uniform/leaf-area average predictions using the Leuning (1995) stomatal model are 1.35, 1.29 and 1.09 mmol m^{-2} s^{-1} under low, medium and high CO_2 concentrations, respectively (the heat maps are not shown).

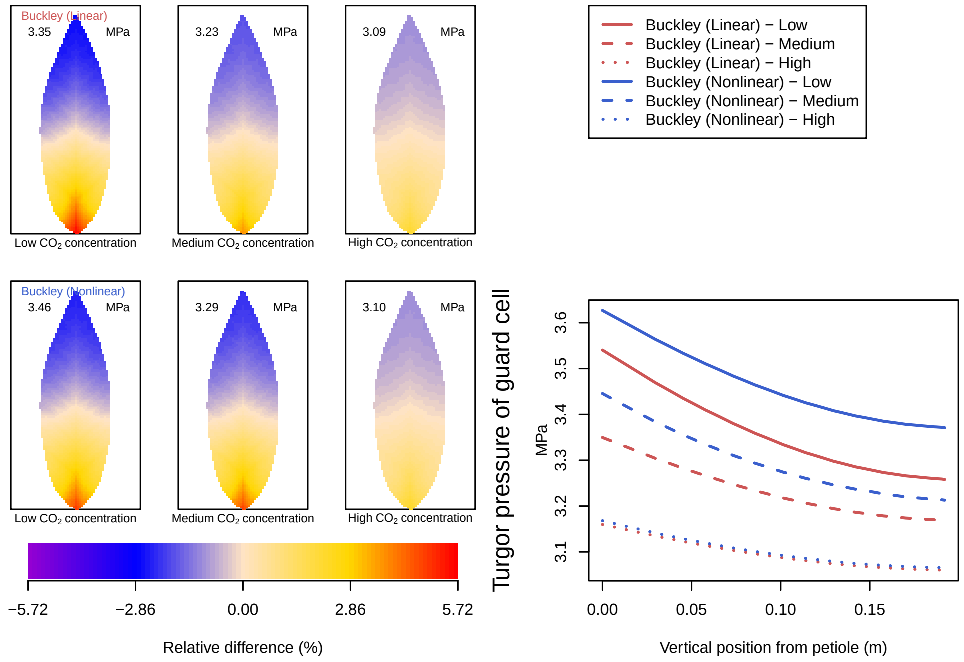

Figure S15. A comparison of model predictions of guard cell turgor pressure distributed over the leaf for different values of CO_2 concentration. Details as in Figure 6 of the paper, except for a longer/narrower leaf of aspect ratio $l : w = 1 : 0.28$.

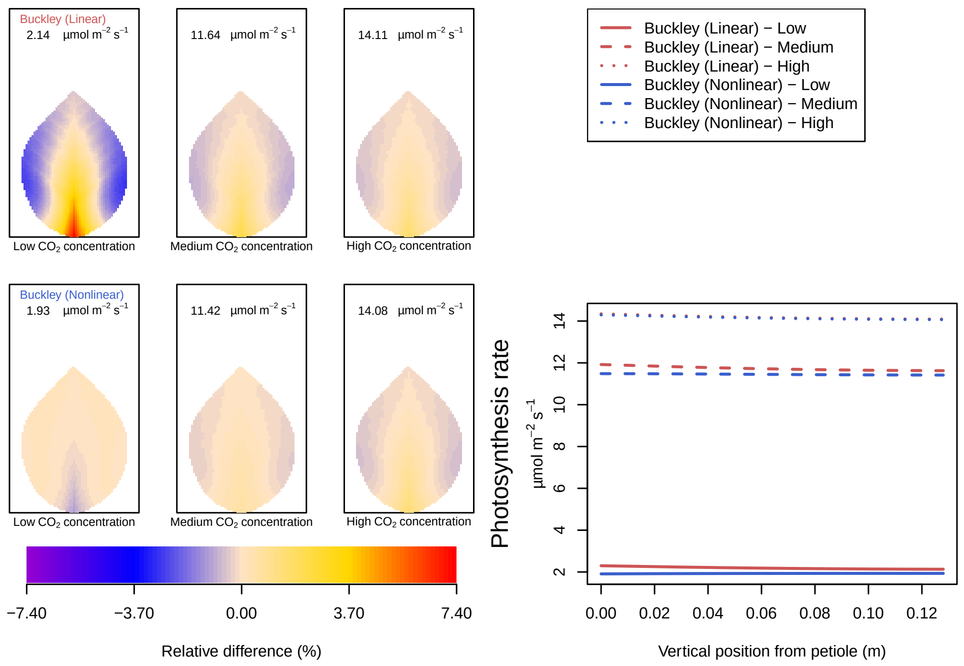

Figure S16. A comparison of model predictions of photosynthesis rate distributed over the leaf for different values of CO_2 concentration. Details as in Figure 4 of the paper, except for a shorter/wider leaf of aspect ratio $l : w = 1 : 0.63$. In this case the uniform/leaf-area average predictions using the Leuning (1995) stomatal model are 2.16, 11.81 and 16.73 μmol m^{-2} s^{-1} under low, medium and high CO_2 concentrations, respectively (the heat maps are not shown).

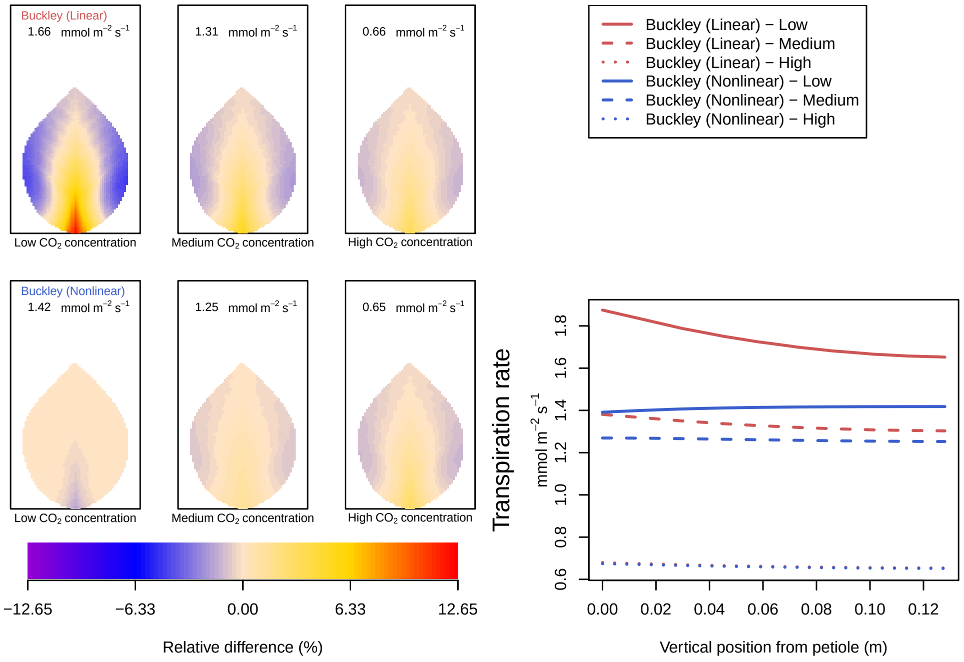

Figure S17. A comparison of model predictions of transpiration rate ($-F_{\mathrm{T}}$) distributed over the leaf for different values of CO_2 concentration. Details as in Figure 5 of the paper, except for a shorter/wider leaf of aspect ratio $l : w = 1 : 0.63$. In this case the uniform/leaf-area average predictions using the Leuning (1995) stomatal model are 1.35, 1.29 and 1.09 mmol m^{-2} s^{-1} under low, medium and high CO_2 concentrations, respectively (the heat maps are not shown).

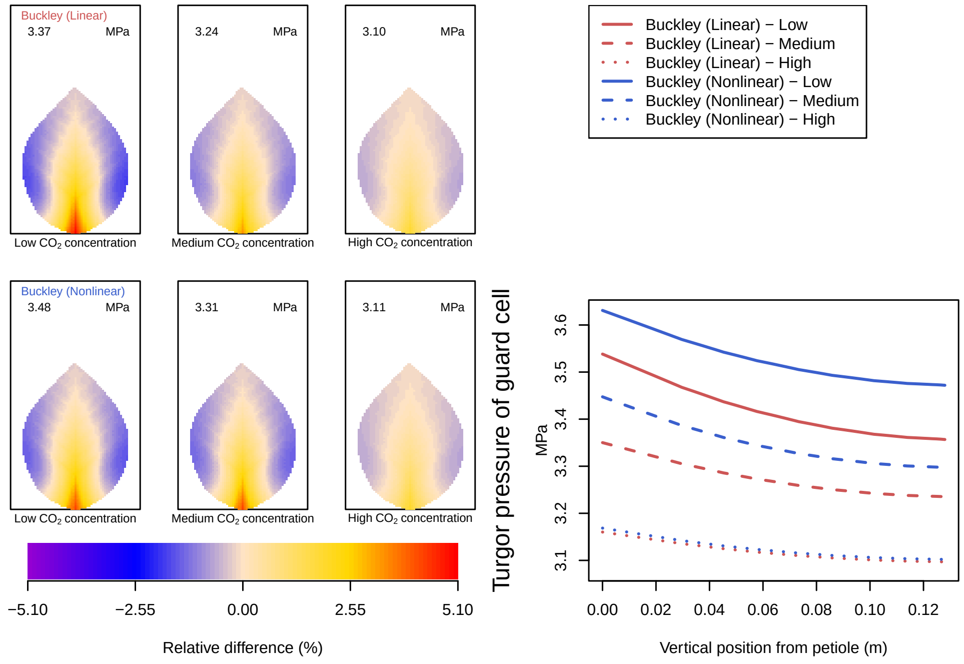

Figure S18. A comparison of model predictions of turgor pressure of guard cells distributed over the leaf for different values of CO_2 concentration. Details as in Figure 6 of the paper, except for a shorter/wider leaf of aspect ratio $l : w = 1 : 0.63$.

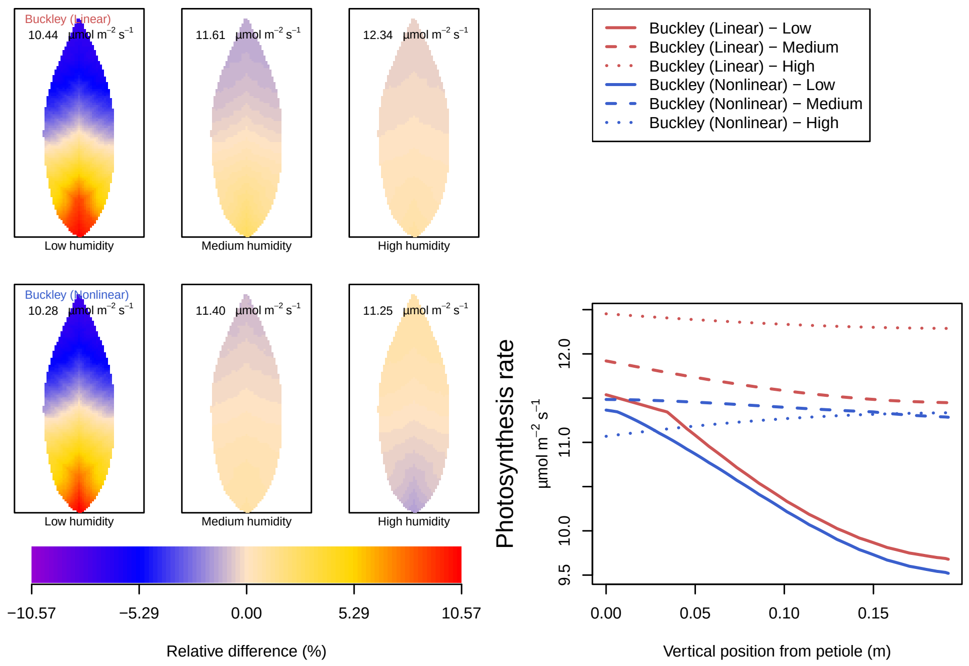

Figure S19. A comparison of model predictions of photosynthetic activity distributed over the leaf for different values of humidity. Details as in Figure 7, except for a narrower/longer leaf of aspect ratio $l : w = 1 : 0.28$.

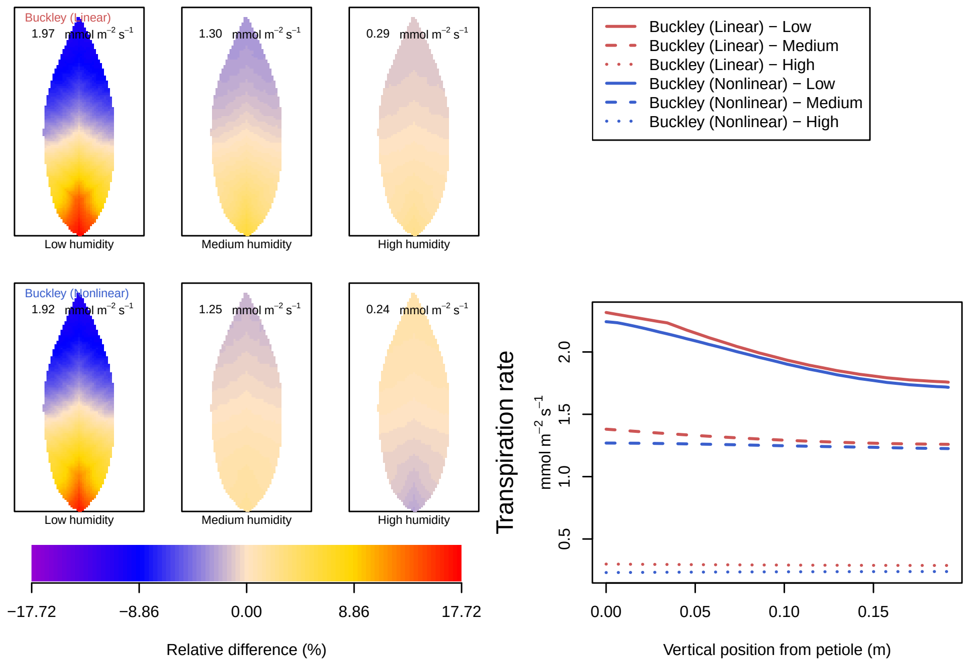

Figure S20. A comparison of model predictions of transpiration rate ($-F_{\mathrm{T}}$) distributed over the leaf for different values of humidity. Details as in Figure 8 of the paper, except for a longer/narrower leaf of aspect ratio $l : w = 1 : 0.28$. In this case the uniform/leaf-area average predictions using the Leuning (1995) stomatal model are 1.87, 1.29 and 0.40 mmol m^{-2} s^{-1} under low, medium and high humidity, respectively (the heat maps are not shown).

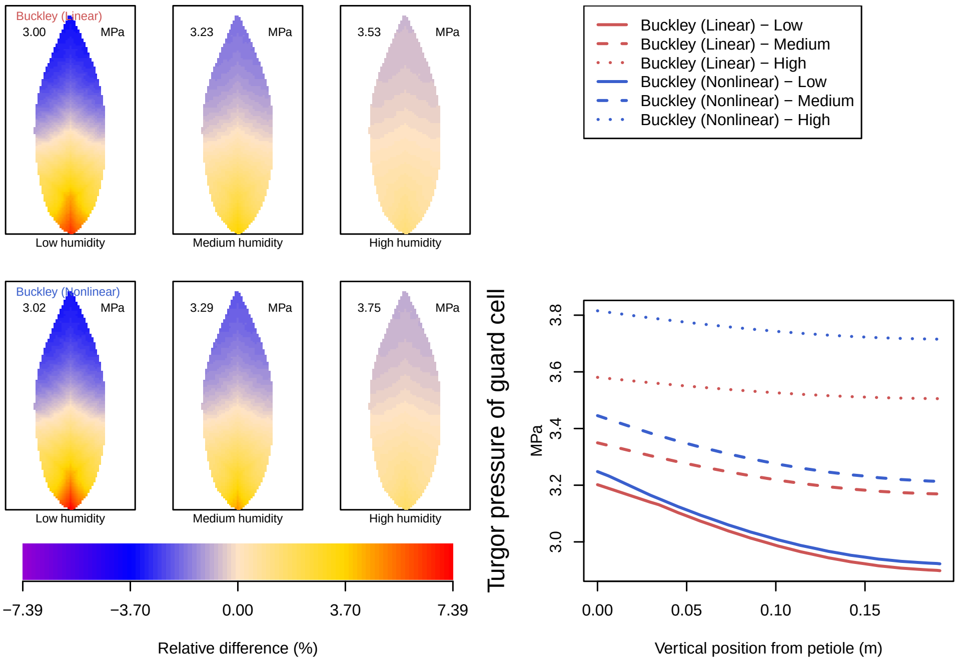

Figure S21. A comparison of model predictions of guard cell turgor pressure distributed over the leaf for different values of humidity. Details as in Figure S4, except for a longer/narrower leaf of aspect ratio $l : w = 1 : 0.28$.

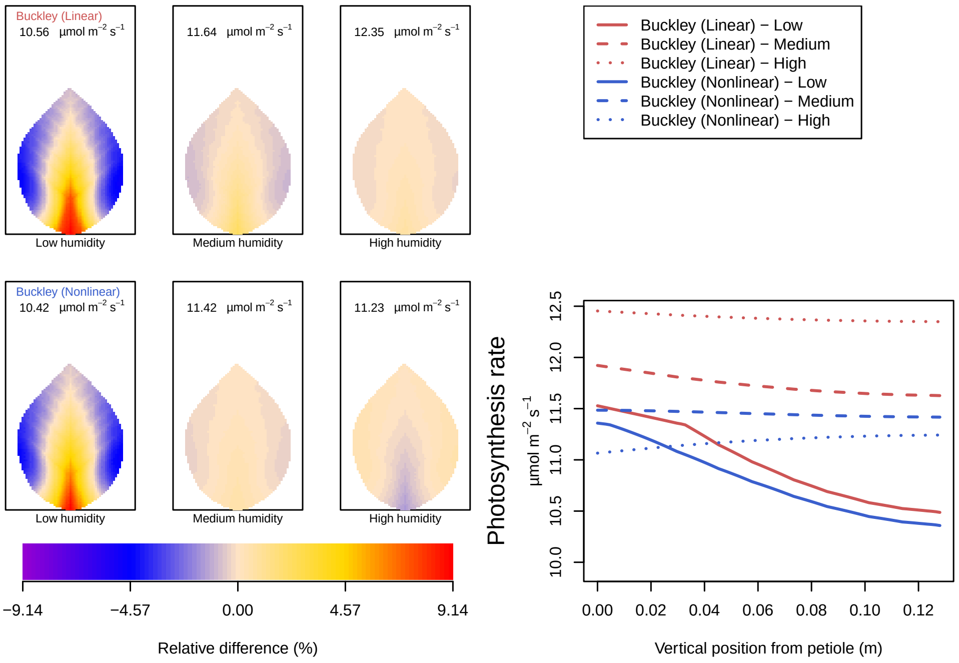

Figure S22. A comparison of model predictions of photosynthetic activity distributed over the leaf for different values of humidity. Details as in Figure 7, except for a wider/shorter leaf of aspect ratio $l : w = 1 : 0.63$.

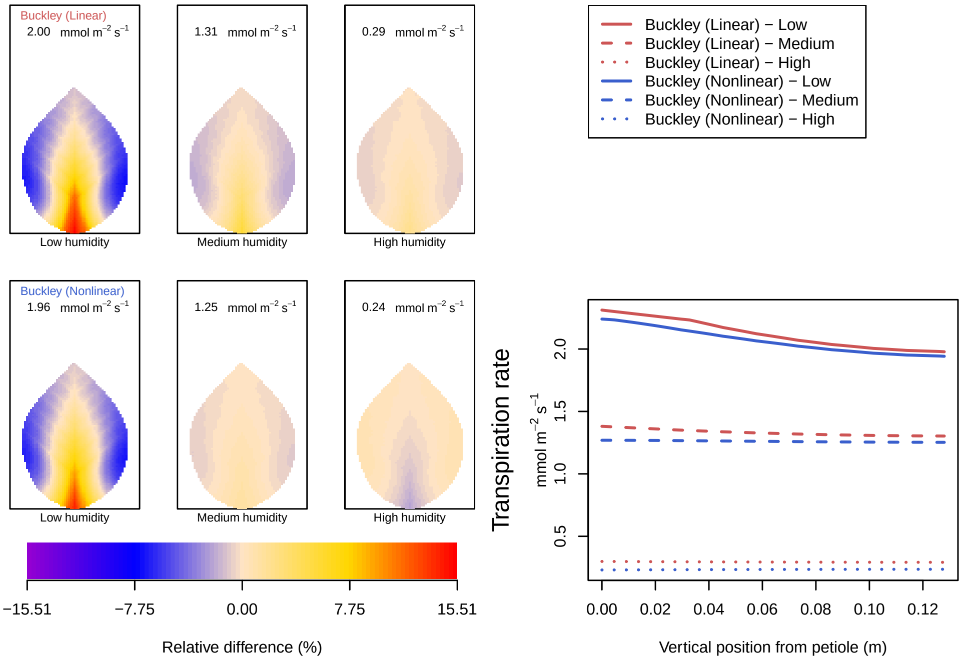

Figure S23. A comparison of model predictions of transpiration rate ($-F_{\mathrm{T}}$) distributed over the leaf for different values of humidity. Details as in Figure 8 of the paper, except for a shorter/wider leaf of aspect ratio $l : w = 1 : 0.63$. In this case the uniform/leaf-area average predictions using the Leuning (1995) stomatal model are 1.87, 1.29 and 0.40 mmol m^{-2} s^{-1} under low, medium and high humidity, respectively (the heat maps are not shown).

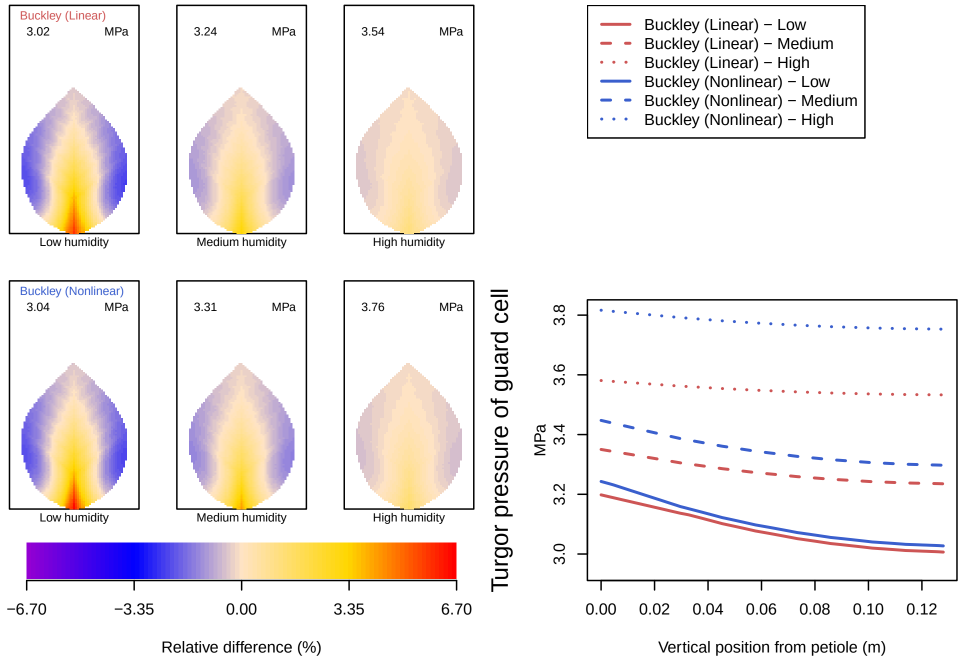

Figure S24. A comparison of model predictions of turgor pressure of guard cells distributed over the leaf for different values of humidity. Details as in Figure S4, except for a shorter/wider leaf of aspect ratio $l : w = 1 : 0.63$.

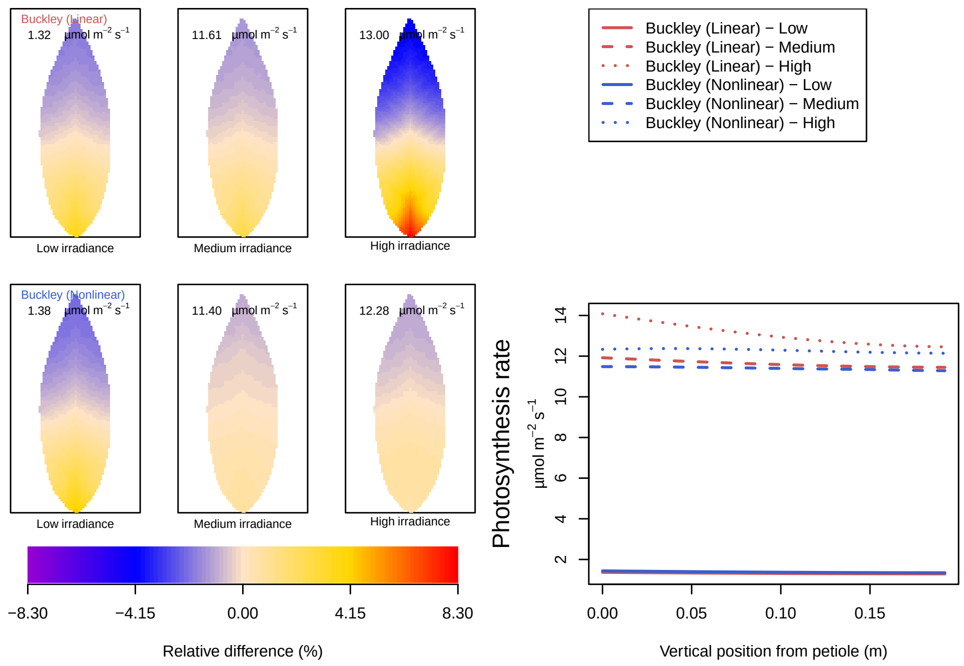

Figure S25. A comparison of model predictions of photosynthesis rate distributed over the leaf for different values of light intensity. Details as in Figure 9 of the paper, except for a longer/narrower leaf of aspect ratio $l : w = 1 : 0.28$. In this case the uniform/leaf-area average predictions using the Leuning (1995) stomatal model are 5.25, 11.81 and 12.05 μmol m^{-2} s^{-1} under low, medium and high light intensity, respectively (the heat maps are not shown).

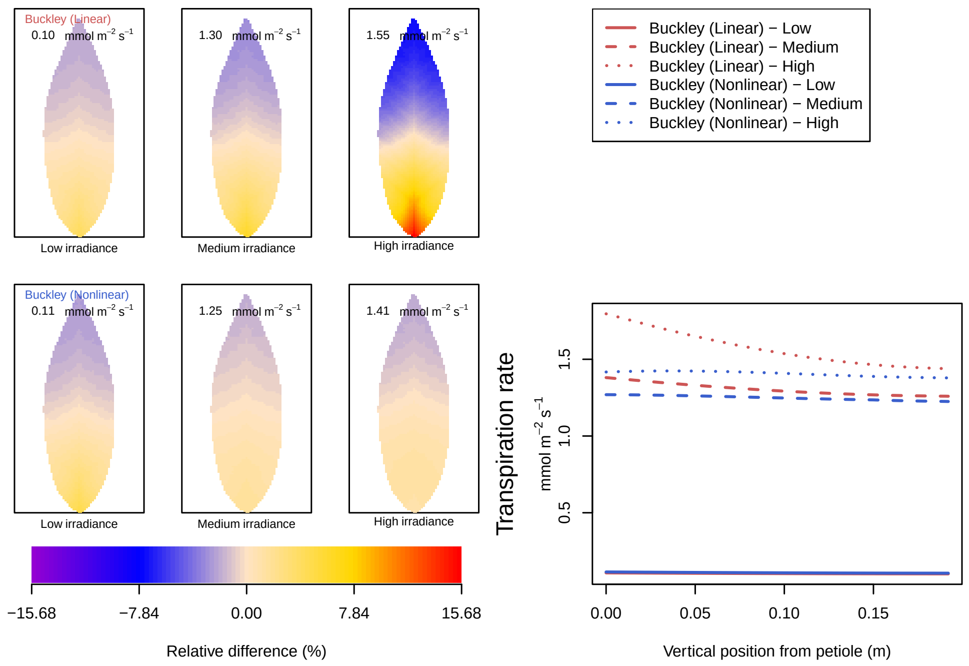

Figure S26. A comparison of model predictions of transpiration rate ($-F_\mathrm{T}$) distributed over the leaf for different values of light intensity. Details as in Figure 10 of the paper, except for a longer/narrower leaf of aspect ratio $l : w = 1 : 0.28$. In this case the uniform/leaf-area average predictions using the Leuning (1995) stomatal model are 0.98, 1.29 and 1.30 mmol m^{-2} s^{-1} under low, medium and high light intensity, respectively (the heat maps are not shown).

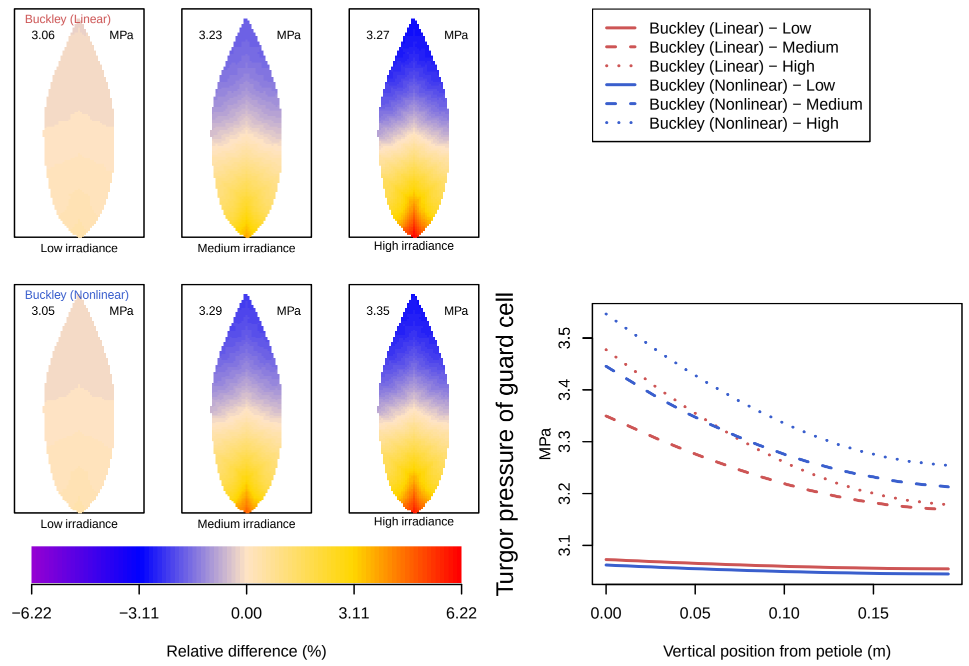

Figure S27. A comparison of model predictions of guard cell turgor pressure distributed over the leaf for different values of light intensity. Details as in Figure S5, except for a longer/narrower leaf of aspect ratio $l : w = 1 : 0.28$.

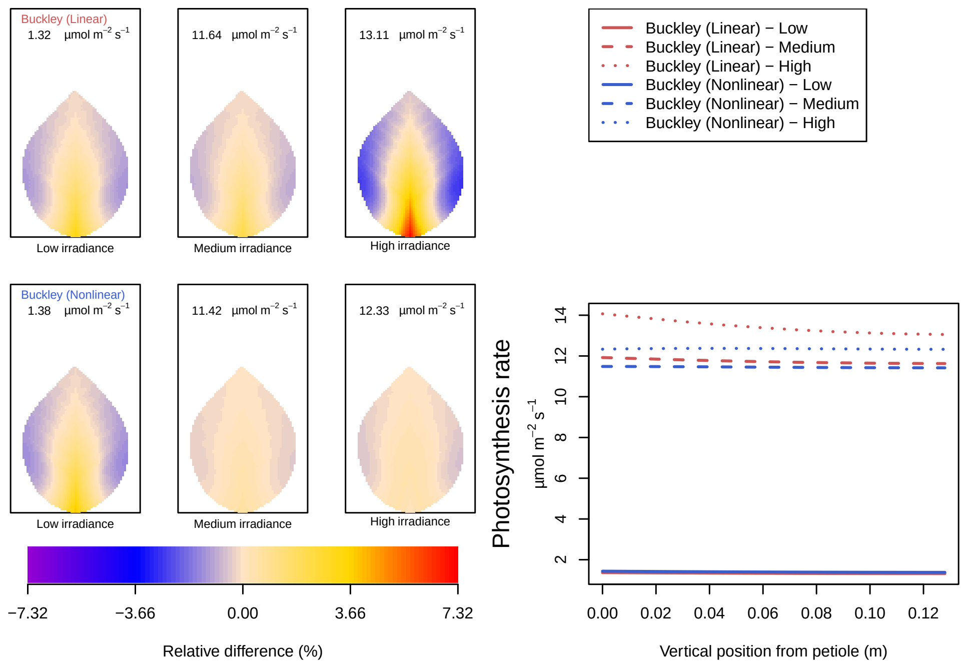

Figure S28. A comparison of model predictions of photosynthesis rate distributed over the leaf for different values of light intensity. Details as in Figure 9 of the paper, except for a shorter/wider leaf of aspect ratio $l : w = 1 : 0.63$. In this case the uniform/leaf-area average predictions using the Leuning (1995) stomatal model are 5.25, 11.81 and 12.05 μmol m^{-2} s^{-1} under low, medium and high light intensity, respectively (the heat maps are not shown).

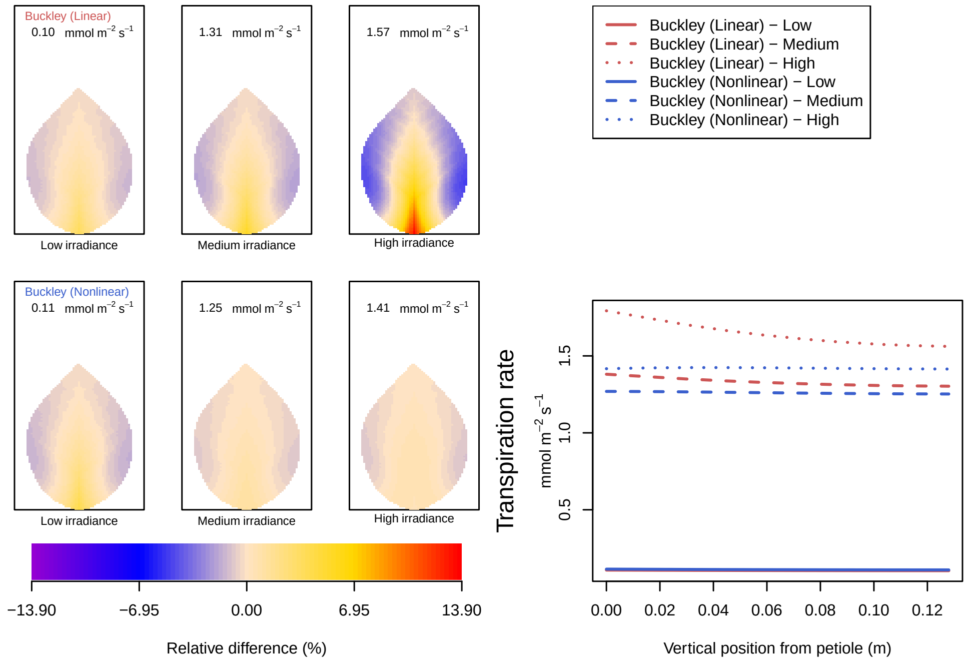

Figure S29. A comparison of model predictions of transpiration rate ($-F_{\mathrm{T}}$) distributed over the leaf for different values of light intensity. Details as in Figure 10 of the paper, except for a shorter/wider leaf of aspect ratio $l : w = 1 : 0.63$. In this case the uniform/leaf-area average predictions using the Leuning (1995) stomatal model are 0.98, 1.29 and 1.30 mmol m^{-2} s^{-1} under low, medium and high light intensity, respectively (the heat maps are not shown).

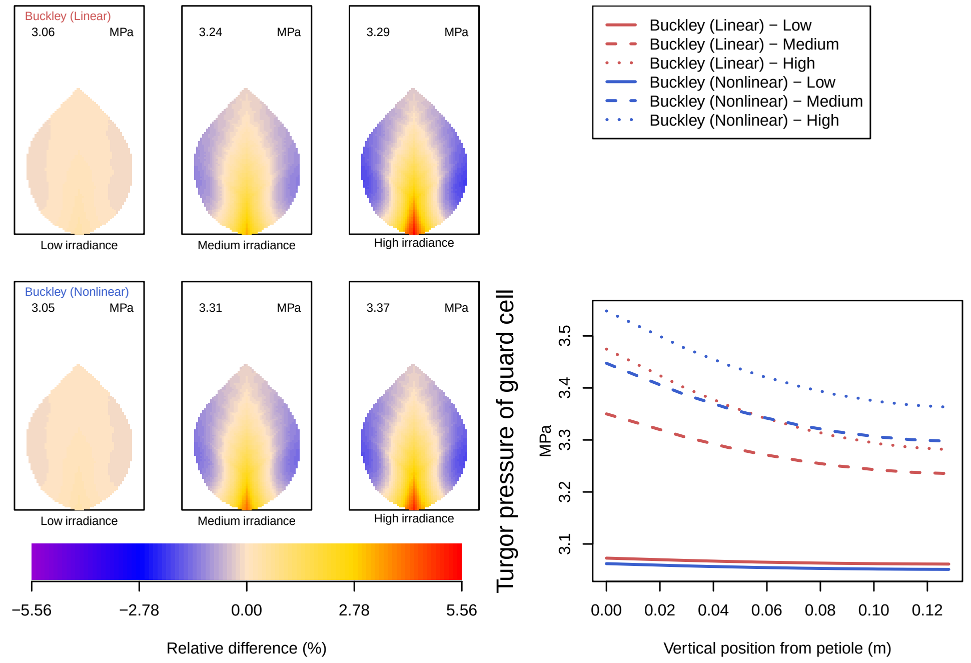

Figure S30. A comparison of model predictions of turgor pressure of guard cells distributed over the leaf for different values of light intensity. Details as in Figure S5, except for a shorter/wider leaf of aspect ratio $l : w = 1 : 0.63$.

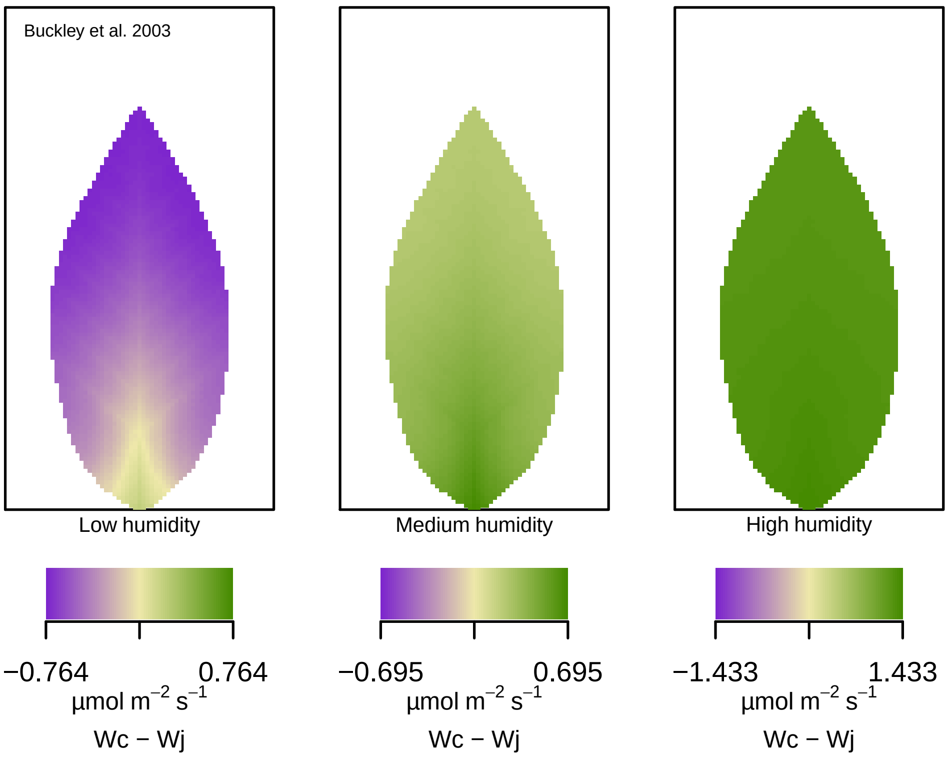

Figure S31. An intermediate technical result found using the Buckley et al. (2003) model of stomatal conductance. The figure shows the variation of carboxylation rates $W_c - W_j$ across the leaf and its dependence on external relative humidity. The three panels of results, from left-to-right, are found under low (10%), medium (50%) and high humidity (90%).

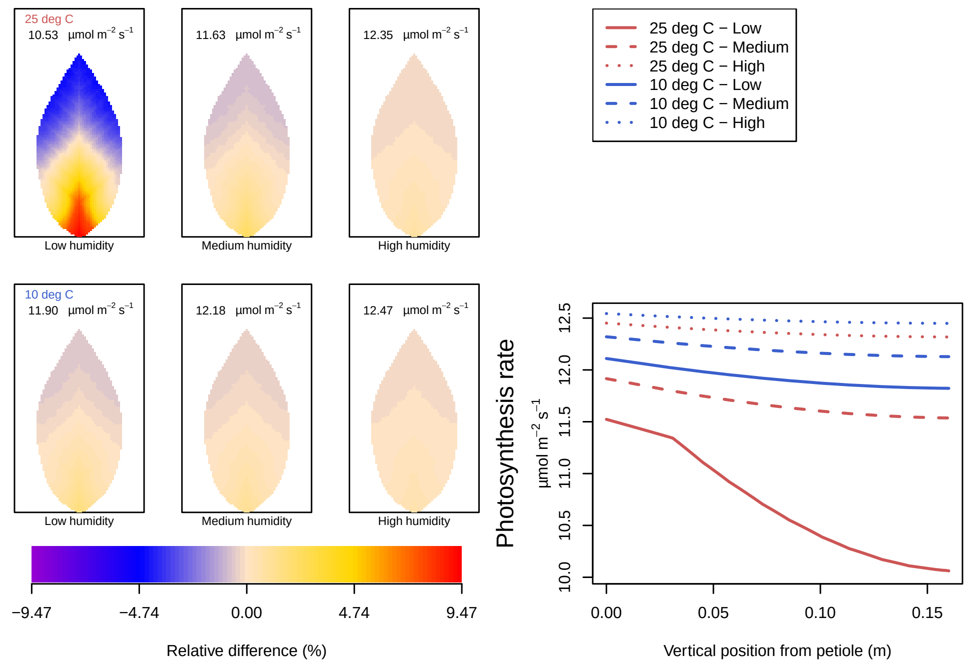

Figure S32. A comparison of model predictions of photosynthesis rate distributed over the leaf for different values of humidity with **linear Buckley et al. model**. Details as in Figure 7 of the paper, except for comparing normal temperature (10° C) and low temperature (25° C).

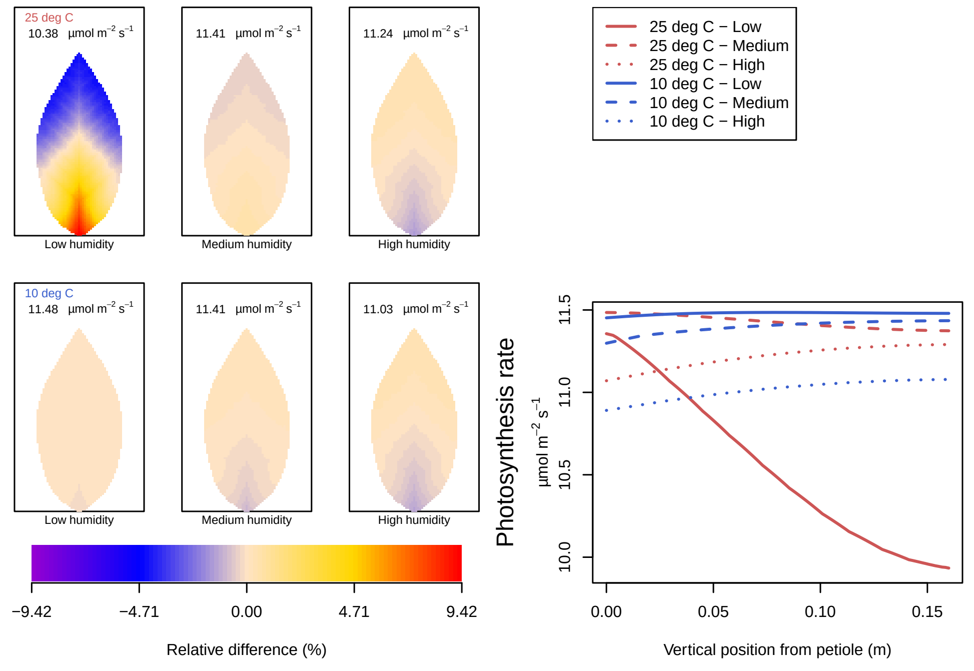

Figure S33. A comparison of model predictions of photosynthesis rate distributed over the leaf for different values of humidity with **nonlinear Buckley et al. model**. Details as in Figure 7 of the paper, except for comparing normal temperature (10° C) and low temperature (25° C).

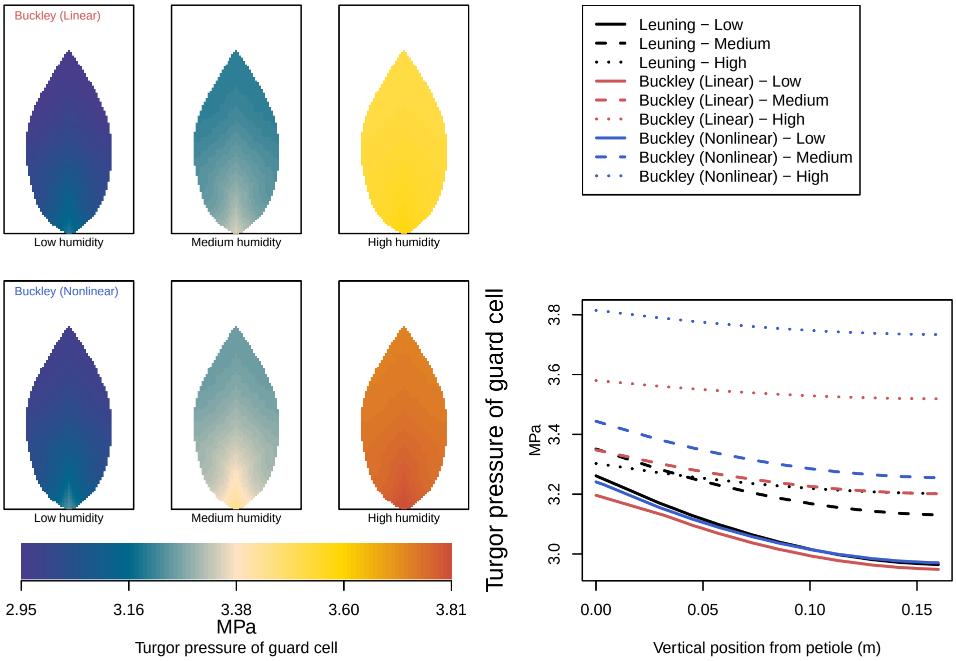

Figure S34. A comparison of predictions of guard cell turgor pressure distributed over the leaf according to the three stomatal conductance models of Leuning (1995) (black lines in right hand figure), linear Buckley et al. (2003) (top row of panels, and red lines in the right hand figure), and nonlinear Buckley et al. (2003) (bottom row of panels, and blue lines in right hand figure). The 2D maps shown are **the absolute value of the turgor pressure**. The three columns (left-to-right) and line styles (solid, dashed, dotted) show the respective dependencies on relative humidity: low (10 %) ; medium (50 %); and high humidity (90 %). Other model parameter values can be found in Supplementary Information I.

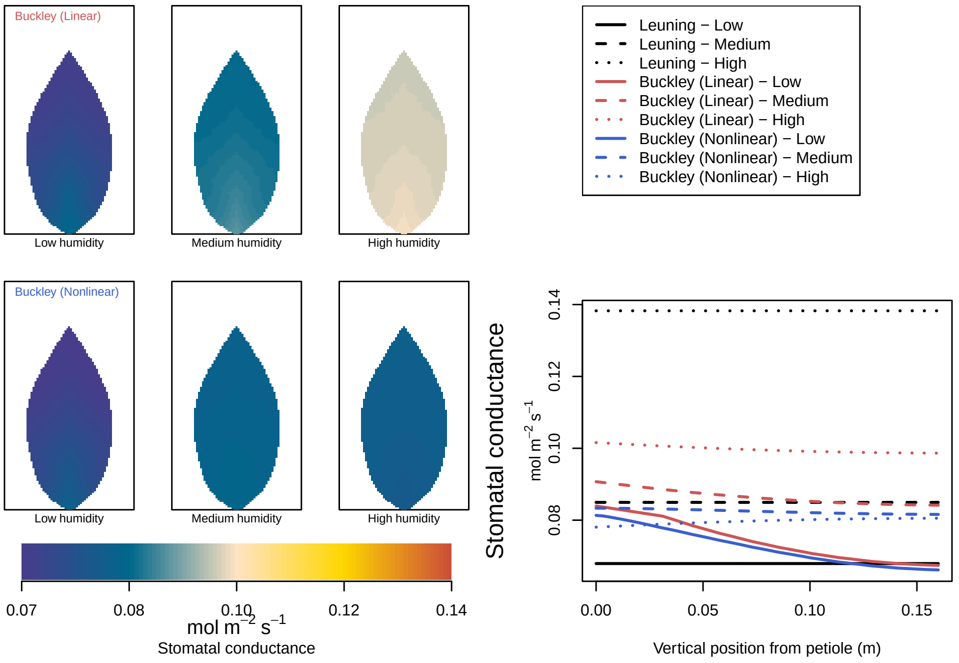

Figure S35. A comparison of predictions of stomatal conductance distributed over the leaf according to the three stomatal conductance models of Leuning (1995) (black lines in right hand figure), linear Buckley et al. (2003) (top row of panels, and red lines in the right hand figure), and nonlinear Buckley et al. (2003) (bottom row of panels, and blue lines in right hand figure). The 2D maps shown are **the absolute value of the stomatal conductance**. The three columns (left-to-right) and line styles (solid, dashed, dotted) show the respective dependencies on relative humidity: low (10 %) ; medium (50 %); and high humidity (90 %). Other model parameter values can be found in Supplementary Information I.

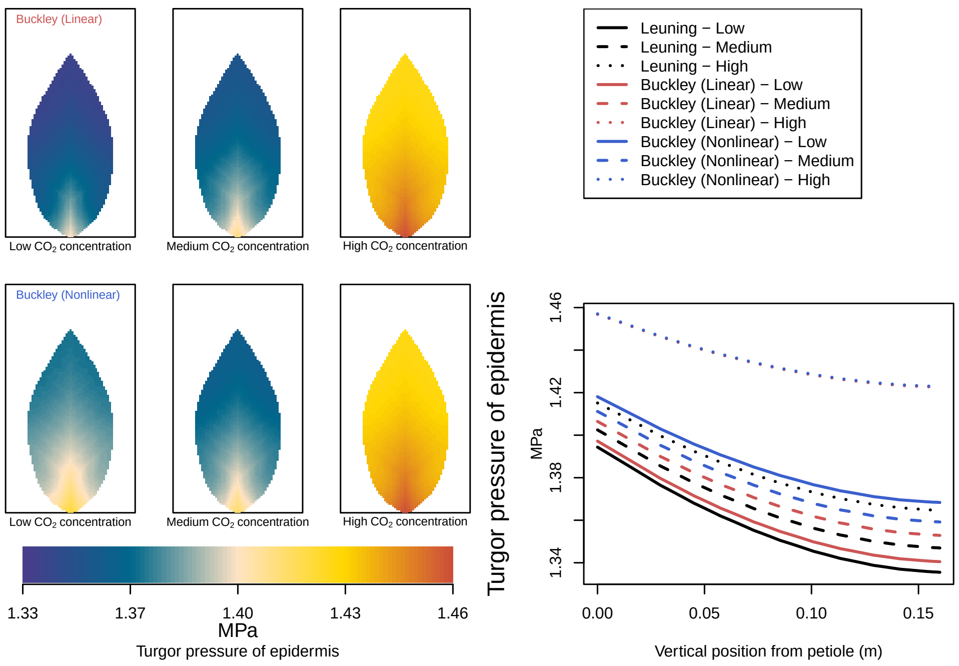

Figure S36. A comparison of predictions of epidermal turgor pressure distributed over the leaf according to the three stomatal conductance models of Leuning (1995) (black lines in right hand figure), linear Buckley et al. (2003) (top row of panels, and red lines in the right hand figure), and nonlinear Buckley et al. (2003) (bottom row of panels, and blue lines in right hand figure). The 2D maps shown are **the absolute value of the epidermal turgor pressure**. The three columns (left-to-right) and line styles (solid, dashed, dotted) show the respective dependencies on **external gas concentration of CO_2**: low (100 ppm), medium (400 ppm) and high concentration (800 ppm). Other model parameter values can be found in Supplementary Information I.
